# Autocrine Effects of Brain Endothelial Cell-Produced Human Apolipoprotein E on Metabolism and Inflammation *in vitro*

**DOI:** 10.3389/fcell.2021.668296

**Published:** 2021-06-10

**Authors:** Felecia M. Marottoli, Troy N. Trevino, Xue Geng, Zarema Arbieva, Pinal Kanabar, Mark Maienschein-Cline, James C. Lee, Sarah E. Lutz, Leon M. Tai

**Affiliations:** ^1^Department of Anatomy and Cell Biology, University of Illinois at Chicago, Chicago, IL, United States; ^2^Department of Bioengineering, University of Illinois at Chicago, Chicago, IL, United States; ^3^Genome Research Core, Research Resources Center, University of Illinois at Chicago, Chicago, IL, United States; ^4^Research Informatics Core, Research Resources Center, University of Illinois at Chicago, Chicago, IL, United States

**Keywords:** apolipoprotein E, brain endothelial cells, metabolism, inflammation, SR9009

## Abstract

Reports of *APOE4*-associated neurovascular dysfunction during aging and in neurodegenerative disorders has led to ongoing research to identify underlying mechanisms. In this study, we focused on whether the *APOE* genotype of brain endothelial cells modulates their own phenotype. We utilized a modified primary mouse brain endothelial cell isolation protocol that enabled us to perform experiments without subculture. Through initial characterization we found, that compared to *APOE3*, *APOE4* brain endothelial cells produce less apolipoprotein E (apoE) and have altered metabolic and inflammatory gene expression profiles. Further analysis revealed *APOE4* brain endothelial cultures have higher preference for oxidative phosphorylation over glycolysis and, accordingly, higher markers of mitochondrial activity. Mitochondrial activity generates reactive oxygen species, and, with *APOE4*, there were higher mitochondrial superoxide levels, lower levels of antioxidants related to heme and glutathione and higher markers/outcomes of oxidative damage to proteins and lipids. In parallel, or resulting from reactive oxygen species, there was greater inflammation in *APOE4* brain endothelial cells including higher chemokine levels and immune cell adhesion under basal conditions and after low-dose lipopolysaccharide (LPS) treatment. In addition, paracellular permeability was higher in *APOE4* brain endothelial cells in basal conditions and after high-dose LPS treatment. Finally, we found that a nuclear receptor Rev-Erb agonist, SR9009, improved functional metabolic markers, lowered inflammation and modulated paracellular permeability at baseline and following LPS treatment in *APOE4* brain endothelial cells. Together, our data suggest that autocrine signaling of apoE in brain endothelial cells represents a novel cellular mechanism for how *APOE* regulates neurovascular function.

## Introduction

Human *APOE* genotype plays an important role in the homeostasis of the central nervous system and has long been linked to neurodegenerative disorders (Huang, 2006; Mahley et al., 2007; Yamazaki et al., 2019; Chen et al., 2020; Lanfranco et al., 2020; Lewandowski et al., 2020; Li et al., 2020). For example, *APOE4* is associated with greater cognitive decline in aging, poorer outcomes following stroke and traumatic brain injury, and is a major genetic risk factor for Alzheimer’s disease compared to *APOE3* (reviewed in Mahley et al., 2007; Liu et al., 2013; Flowers and Rebeck, 2020). One way *APOE* genotype could impact neuronal function is through neurovascular disruption, which is found with *APOE4* during aging, in Alzheimer’s disease and in respective mouse models (Salloway et al., 2002; Zipser et al., 2007; Poels et al., 2010; Halliday et al., 2013; Zlokovic, 2013; Halliday et al., 2015; Tai et al., 2016; Thomas et al., 2016; Marottoli et al., 2017; Tai et al., 2017; Thomas et al., 2017). Since specialized brain endothelial cells are central for the unique properties of the neurovasculature, it is important to identify mechanisms through which *APOE* alters brain endothelial cell function.

Current research has focused on how the *APOE* genotype of astrocytes and pericytes (Bell et al., 2012; Yamazaki et al., 2020b) impact brain endothelial cells; less explored and more controversial, is whether the *APOE* genotype of brain endothelial cells is important. Indeed, limited *in vitro* data conflict on whether endothelial cells produce apolipoprotein E (apoE) to any significant level and if there are any *APOE* genotype-specific functional differences (Rieker et al., 2019; Yamazaki et al., 2020b). In our opinion, a case can be made for the concept that brain endothelial cell-produced apoE contributes to regulation of neurovascular function. In general, evidence that cells beyond astrocytes and hepatocytes produce apoE to impact cellular function is expanding (Basu et al., 1982; Werb et al., 1986; Mahley et al., 2007; Fernandez et al., 2019; Najm et al., 2019; Yamazaki et al., 2020a, b). In these other cell types, apoE modulates basal and reparative processes, particularly metabolism and inflammation, which may be important for brain endothelial cells given their specialized function and location. As a simplification, brain endothelial cells control bi-directional movement of essential and unwanted molecules to and from the brain, are functionally linked to neuronal activity, integrate signals with multiple cell types and regulate inflammation. Brain endothelial cells are also continually exposed to acute and chronic fluctuations in circulating molecules from the interstitial fluid and plasma in physiological and pathological states. Brain endothelial cells may utilize fundamental metabolic and inflammatory functions of apoE for local homeostasis. Thus, for such an important protein as apoE, and in a cell as specialized as brain endothelial cells, there is a distinct advantage of autocrine signaling. Resolving the question of whether apoE functions in an autocrine manner in brain endothelial cells could provide a novel cellular mechanism for how *APOE* regulates neurovascular function both in physiological and pathological states.

The objective of the present study was to determine the role of human *APOE* genotype in modulating the phenotype of brain endothelial cells *in vitro*. To this end, we isolated primary mouse brain endothelial cells from *APOE3-* and *APOE4*-targeted replacement mice and assessed genotype-specific differences in apoE levels, transcriptomic profiles and cellular functions, including metabolism and inflammation, using biochemical and immunocytochemical assays.

## Materials and Methods

Supplementary File 1 is a comprehensive “Materials and Methods” section containing protocols for brain endothelial cell isolation, *in situ* hybridization, immunocytochemistry, western blot analysis, RNA sequencing, metabolomics, atomic force microscopy and leukocyte adhesion assays. In addition, in Supplementary Table 1 we detail specific growth surfaces, sample preparation, modifications to commercially available assay kit protocols and quantification methods for each figure panel. Supplementary File 1 also contains a results table and five figures (one of which is full western blot images). Supplementary File 2 is an excel file containing all raw data and statistical analysis tables. Thus, here we provide a brief description of the brain endothelial isolation protocol, and a summary of the assays.

### Primary Brain Endothelial Cell Cultures

All experiments were approved by the Institutional Animal Care and Use Committee at the University of Illinois at Chicago. Primary cortical mouse brain endothelial cells were isolated from male and female human *APOE3*- and *APOE4*-targeted replacement mice (Taconic, 1548 and 1549, respectively). Due to our focus on *APOE* genotype we isolated cells from juvenile mice to limit the influence of sex hormones. Briefly, cerebral cortices dissected from 28-day-old mice were diced, centrifuged (1,000 × *g*, 5 min, 4°C), resuspended in papain (20 U/ml)/DNase (2,000 U/ml) and lightly triturated through a 19G needle. Brain homogenates were then incubated for exactly 15 min at 37°C, triturated with a 21G needle, mixed thoroughly with 2 ml of 25% BSA per cortex, vortexed and centrifuged (4,000 × *g*, 5 min, 4°C) to separate out the myelin. The supernatant was collected, vortexed, and centrifuged a second time (4,000 × *g*, 5 min, 4°C). Pellets from both centrifugations were combined in complete growth media (EBM-2 containing EGM^TM^ -2 MV Microvascular Endothelial SingleQuots^TM^ and 5.5 U/ml heparin), passed through a 100 μm cell strainer and pelleted (1,000 × *g*, 5 min, 4°C). Resuspended cells were plated at approximately 1 cortex (i.e., 2 hemispheres) to 3.466 cm^2^ on a growth matrix of fibronectin, collagen and laminin, and placed at 37°C with 5% CO_2_. 4 to 6 h after plating, red blood cells and debris were very gently washed away and attached cells incubated overnight at 37°C in heparin-supplemented complete growth media. The following morning, cells were washed once more and then incubated in 8 μg/ml puromycin for 48 h to negatively select for brain endothelial cells. After puromycin was removed, brain endothelial cells were washed and grown to confluence in complete growth media.

Experiments followed the general timeline of isolation on day 0, puromycin added on day 1, puromycin removed on day 3, media changed on day 5 (±5 μM SR9009), lipopolysaccharide (LPS from *E. coli* O8:K27 (S-form)) spiked into the media on day 6 as required, and experiments conducted on day 7.

### Summary of Assays Used for Evaluation of Brain Endothelial Cell Phenotypes

**FIGURE 1:**
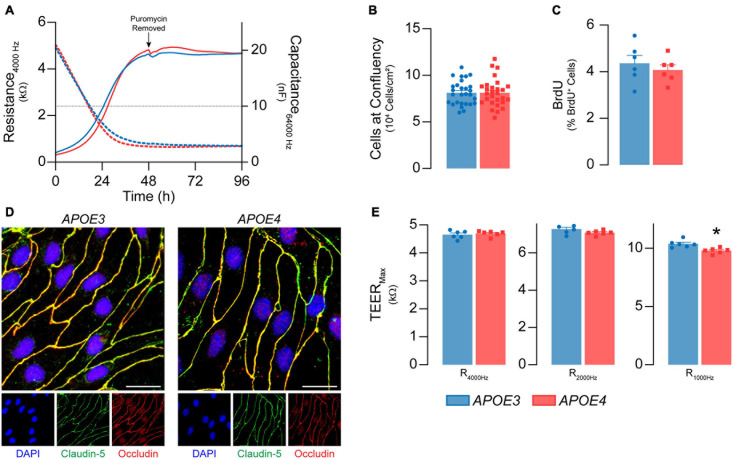
Primary mouse brain endothelial cell characterization at passage 0. **(A)** Following isolation, brain endothelial cells establish a confluent monolayer. Capacitance (dashed lines) measured at 64 kHz reflects cell attachment and spreading and decreases as cell coverage increases; capacitance plateaus below 10 nF (black dotted line) once a monolayer is established (ECIS^®^ ZΘApplied Biosystems). *APOE3* and *APOE4* brain endothelial cell cultures establish a monolayer by 48 h, with no genotype differences. At confluence, there are no differences in **(B)** cell density (*n* = 28) or **(C)** BrdU^+^ nuclei (<5%) between *APOE3* and *APOE4* brain endothelial cells. **(D)** *APOE3* and *APOE4* brain endothelial cells express the tight junction protein markers claudin-5 (green) and occludin (red), and exhibit classical endothelial cell morphology (narrow, elongated, tightly packed cells) when assessed by immunocytochemistry, scale bar = 20 μm. **(E)** When impedance is measured at lower frequencies (i.e., 4000, 2000, and 1000 Hz), more current passes between the cells and is therefore a measure of paracellular permeability referred to as transendothelial electrical resistance (TEER). At all frequencies, TEER (solid lines in **A**) progressively increases over the course of 48 h and is then maintained for at least 4 days for both *APOE* genotypes. The frequency that represents paracellular permeability to the greatest extent is cell type-dependent and can be estimated empirically (Stolwijk et al., 2015). 1000 Hz represents the optimal frequency, as the ratio of cell-covered electrode:cell-free electrode is higher (∼22) compared to 2000 Hz (∼20) and 4000 Hz (14) for both *APOE3* and *APOE4* brain endothelial cells. At 1000 Hz TEER values are lower in *APOE4* brain endothelial cells compared to *APOE3*. Data is expressed as mean ± S.E.M. *p < 0.05 by Student’s *t*-test with *n* = 6 (unless otherwise specified above).

**FIGURE 2:**
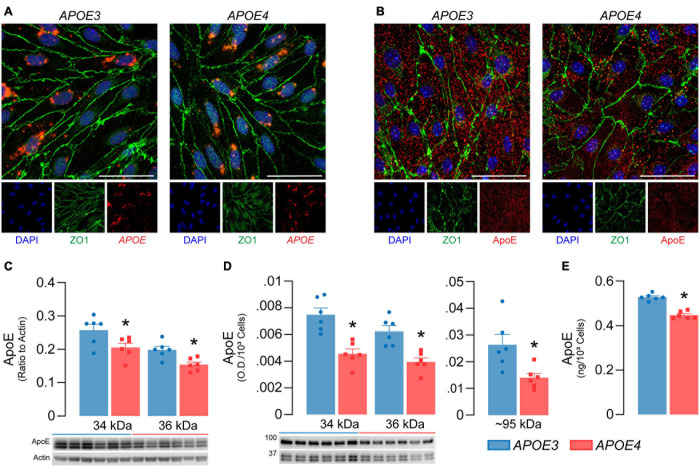
*APOE4* brain endothelial cells produce less apoE compared to *APOE3*. *APOE3* and *APOE4* brain endothelial cells express **(A)** the *APOE* transcript (red) and **(B)** produce apoE protein (red) when assessed by *in situ* hybridization and immunocytochemistry, respectively. Cells were counterstained for the brain endothelial cell marker ZO1 (green) and DAPI (blue); representative confocal Z-stack images were captured at 52X (scale bar = 20 μm). Both **(C)** cell-associated (lysate) and **(D)** secreted (conditioned media) apoE levels are lower with *APOE4* when assessed by western blot analysis. Cell-associated apoE was normalized to actin as a loading control and secreted apoE was loaded as equal volumes and normalized to cell count. **(E)** ApoE levels in the media are ∼15% lower with *APOE4* when quantified by ELISA and normalized to cell count. Data is expressed as mean ± S.E.M. *p < 0.05 by Student’s *t*-test, *n* = 6.

**FIGURE 3:**
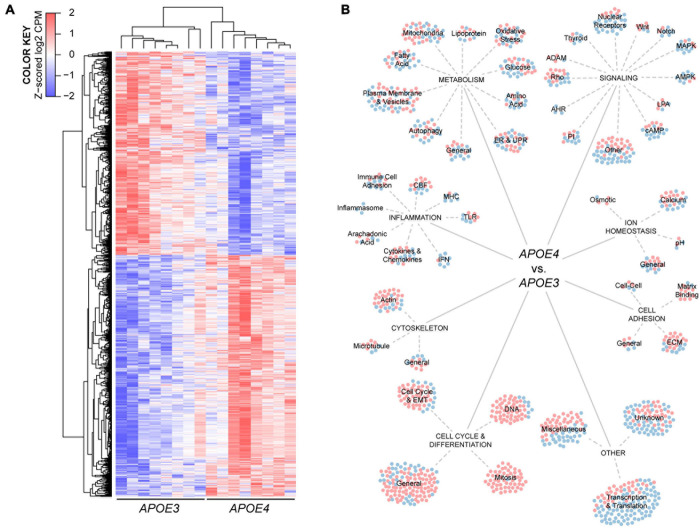
Human *APOE* genotype modulates the transcriptional phenotype of brain endothelial cells. **(A)** Hierarchical cluster analysis of 1304 differentially expressed genes in *APOE4* brain endothelial cells determined by RNA-sequencing analysis, *n* = 8. **(B)** Manual categorization of the differentially expressed genes reveals changes related to metabolism, inflammation, signaling, ion homeostasis, cell adhesion, cytoskeleton, cell cycle as well as other more general functions. Each circle represents a differentially expressed gene, where red indicates higher expression and blue indicates lower expression with *APOE4* compared to *APOE3*.

**FIGURE 4:**
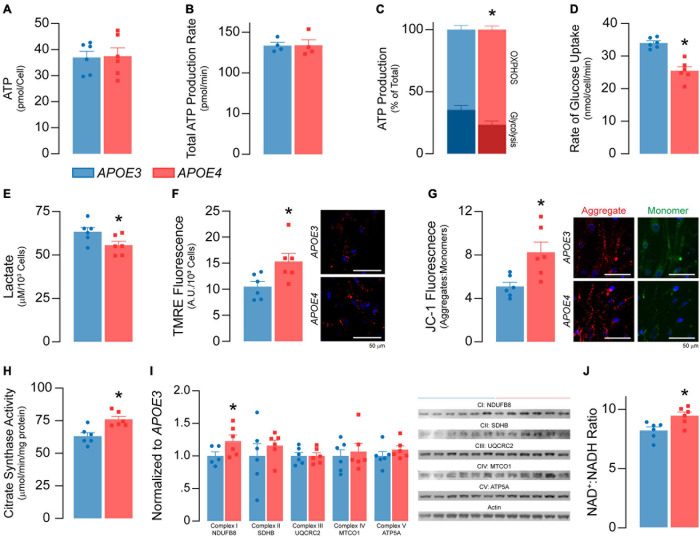
*APOE4* brain endothelial cells have lower glycolysis and higher oxidative phosphorylation and mitochondrial activity compared to *APOE3*. **(A)** ATP levels and **(B)** the rate of total ATP production (*n* = 4) are similar in *APOE3* and *APOE4* brain endothelial cells. Glycolysis and mitochondrial oxidative phosphorylation (OXPHOS) both contribute to ATP production. Comparing the bioenergetic profiles of *APOE3* and *APOE4* brain endothelial cells revealed **(C)** ATP production from OXPHOS is higher and glycolysis is lower with *APOE4* as measured by Seahorse XF Real-Time ATP Rate Assay (*n* = 4). Consistent with lower glycolytic activity, *APOE4* brain endothelial cells also have **(D)** a lower rate of glucose uptake and **(E)** lower levels of lactate in the media compared to *APOE3*. Mitochondrial activity is central to OXPHOS, which may be higher with *APOE4*. We measured mitochondrial activity using complementary approaches. Active mitochondria have a greater net negative charge that can be measured by the accumulation of tetramethylrhodamine ethyl ester (TMRE) and aggregation of JC-1 (monomer, green; aggregate, red) dyes. In *APOE4* brain endothelial cells there is **(F)** higher TMRE staining (∼46%) and **(G)** aggregate:monomer ratio of JC-1 (∼62%). **(H)** Citrate synthase activity, which is important for the first step of the Krebs cycle, is also higher with *APOE4* (∼20%), as are transcripts of electron transport chain components (e.g., mt-Nd1, mt-Nd2, mt-Nd4, mt-Nd5, mt-Cytb, mt-Co1 Supplementary Table 2), which **(I)** we validated at the protein level. **(J)** Nicotinaminde adenine dinucleotide (NAD) is a co-factor present in two forms in a cell; NAD^+^ (oxidized) and NADH (reduced). NADH is utilized in the electron transport chain to donate electrons for ATP generation and as a co-factor for enzymatic activity. The ratio of NAD^+^:NADH is higher with *APOE4*, data that may imply lower NADH levels to supply the cell with energy, or that high levels have been oxidized to sustain higher mitochondrial function. Data is expressed as mean ± S.E.M. *p < 0.05 by Student’s *t*-test with *n* = 6 (unless otherwise specified above).

**FIGURE 5:**
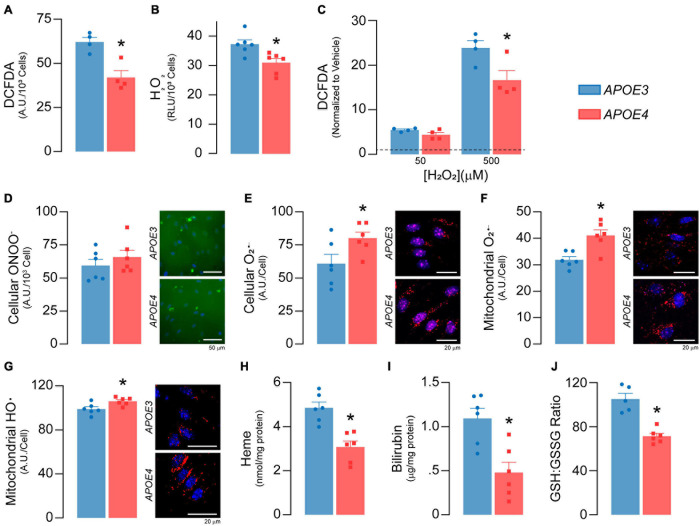
*APOE* genotype modulates reactive oxygen species and antioxidant levels. **(A)** Total reactive oxygen species (ROS) levels were measured with 2′,7′-dichlorofluorescein diacetate (DCFDA; *n* = 4). DCFDA diffuses into the cell where it is deacetylated to a non-fluorescent compound by cellular esterases. Reactive oxygen species then oxidize the deacetylated DCFDA to form 2′, 7′-dichlorofluorescein, which fluoresces green. *APOE4* brain endothelial cells exhibit lower total cellular ROS as evidenced by ∼32% lower DCFDA fluorescence. Of the different types of ROS, H_2_O_2_ is the most abundant and accounts for the majority of the DCFDA signal and **(B)** specific levels of H_2_O_2_ are lower in *APOE4* brain endothelial cells. After the addition of exogenous H_2_O_2_, **(C)** levels of total reactive oxygen species (DCFDA) are still higher with *APOE3* (*n* = 4), suggesting an upregulation of pathways to limit H_2_O_2_ levels in *APOE4* brain endothelial cells, such as peroxisome or other enzymatic activity. For other ROS, while there are **(D)** no changes in peroxynitrite levels with *APOE4*, **(E)** there are ∼31% higher cellular O_2_^–^ levels. These data are consistent with the idea that higher mitochondrial metabolism can result in the accumulation of O_2_^–^. Indeed, specifically for mitochondrial ROS, there are **(F)** ∼29% higher O_2_^–^ (MitoSOX Red O_2_^–^ Indicator which accumulates in mitochondria and is oxidized by O_2_^–^) and **(G)** ∼7% higher hydroxyl radical (OH580 probe which is oxidized by hydroxyl radicals) levels in *APOE4* brain endothelial cells. The effects and levels of reactive oxygen species are in part regulated by antioxidant systems. Heme is an essential iron-containing compound with pleiotropic functions from respiration, oxygen transport and xenobiotic modification to modulation of reactive oxygen species levels. Heme is produced from glycine and succinyl CoA in a series of reactions that starts in the mitochondria, continues in the cytoplasm, and is then completed in the mitochondria. Heme can be degraded into bilirubin, which is an antioxidant and anti-inflammatory molecule. *APOE4* brain endothelial cells have lower levels of **(H)** heme and **(I)** bilirubin, and **(J)** a lower ratio of GSH:GSSG (indicating more oxidative stress; *APOE3 n* = 5, *APOE4 n* = 6). Data is expressed as mean ± S.E.M. *p < 0.05 by Student’s *t*-test with *n* = 6 (unless otherwise specified above).

**FIGURE 6:**
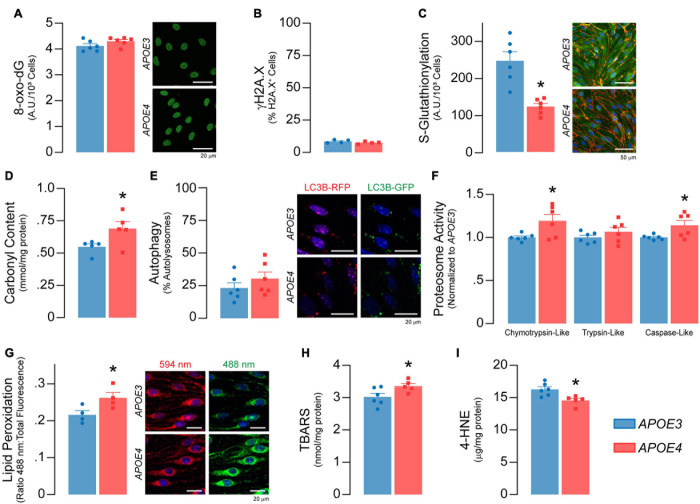
Higher levels of oxidative damage to proteins and lipids in *APOE4* brain endothelial cells. There are no *APOE* genotype effects on markers of DNA damage, including **(A)** levels the oxidized deoxyguanosine derivative 8-oxo-2’-deoxyguanosine and **(B)** the double-stranded DNA breaks marker γH2A.X (*n* = 4). **(C)** Protein glutathionylation limits irreversible oxidative damage to cysteine thiol groups on proteins. *APOE4* brain endothelial cells have ∼50% lower S-glutathionylated protein levels, that could be due to the lower GSH:GSSG ratio induced by reactive oxygen species (Figure 5J) and/or lower levels glutathione transferases (Supplementary Table 2; Gstt3, Gstm2, Gstp1, Gstm1, Gstt2). **(D)** The lower protection of proteins with *APOE4* could lead to irreversible oxidation of amino acid side chains referred to as carbonylation. Protein carbonylation is ∼25% higher with *APOE4* as measured by 2,4-dinitrophenylhydrazine (DNPH) binding (*n* = 5). In addition, while there was no difference in **(E)** autophagy, **(F)** chymotrypsin-like and caspase-like proteosome activity are higher with *APOE4*, potentially to degrade damaged proteins. **(G)** Lipids are sensitive to the effects of reactive oxygen species through peroxidation, which we measured using a ratiometric fluorescent indicator (BODIPY 581/591 C11) that changes from red to green upon lipid peroxidation. In *APOE4* brain endothelial cells, lipid peroxidation is higher, as evidenced by ∼20% higher ratio of green:total fluorescence (*n* = 4). In addition, **(H)** levels of lipid peroxidation products, called TBA reactive substances, are ∼11% higher (*APOE3 n* = 6, *APOE4 n* = 5), although **(I)** 4*-*hydroxynonenal levels are ∼11% lower as assessed by ELISA (*APOE3 n* = 6, *APOE4 n* = 5). Data is expressed as mean ± S.E.M. *p < 0.05 by Student’s *t*-test with *n* = 6 (unless otherwise specified above).

**FIGURE 7:**
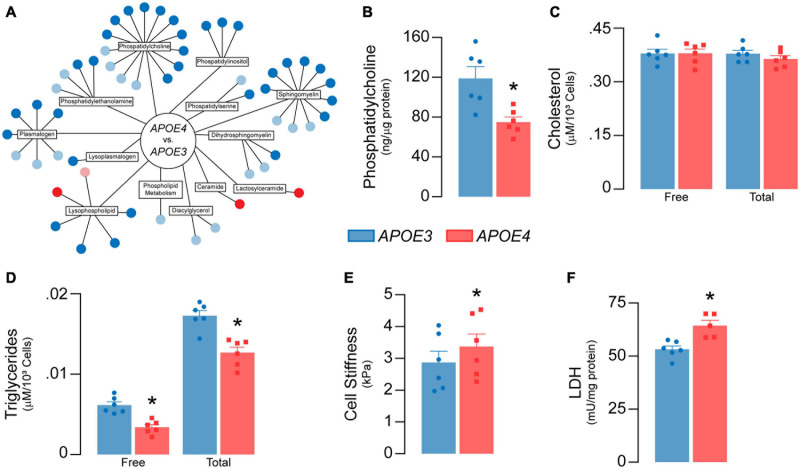
*APOE* genotype modulates lipid metabolism in brain endothelial cells. **(A)** Lipidomic analysis reveals 43 significant differences (dark blue = lower; dark red = higher) and 21 trending differences (light blue = lower; pink = higher) in *APOE4* brain endothelial cells. Each circle represents a unique metabolite. Most of the metabolites are lower with *APOE4* and include phosphatidylcholines (1-myristoyl-2-arachidonyl-GPC (14:0/20:4), phosphatidylethanolamines (1-palmitoyl-2-arachidonyl-GPE (16:0/20:4))), phosphatidylinositols, lysophospholipids (1-oleoyl-GPC (18:1)), and plasmalogens (1-(1-enyl-palmitoyl)-2-arachidonyl-GPC (P16:0/20:4)), with the exception of two ceramides (N-behenoyl-sphingadienine (d18:2/22:0) & actosyl-N-nervonoyl-sphingosine (d18:1/24:1)) that are higher. **(B)** Consistent with lipidomic analysis, phosphatidylcholine levels are lower with *APOE4* when assessed by ELISA. **(C)** Although there are no differences in cellular cholesterol levels, **(D)** cellular triglyceride levels are lower in *APOE4* brain endothelial cells. **(E)** Changes in membrane composition can alter mechanical properties of a cell. In the plasma membrane, this can manifest as changes in stiffness, which can contribute to cell stiffness when measured by atomic force microscopy (Askarova et al., 2013). In this technique, a cantilever tip approaches the plasma membrane, makes contact, and then indents the cell surface; the force required to make the indentation corresponds to cell stiffness. We found that cells stiffness is higher in *APOE4* brain endothelial cells when measured by atomic force microscopy (analyzed by paired *t*-test). **(F)** Lactate dehydrogenase (LDH) is a cytosolic enzyme found in cell culture media when the plasma membrane is damaged, as found with cytotoxicity. Despite the lack of toxicity, levels of lactate dehydrogenase in the media are higher with *APOE4*. Data is expressed as mean ± S.E.M. *p < 0.05 by Student’s *t*-test with *n* = 6 (unless otherwise specified above).

**FIGURE 8:**
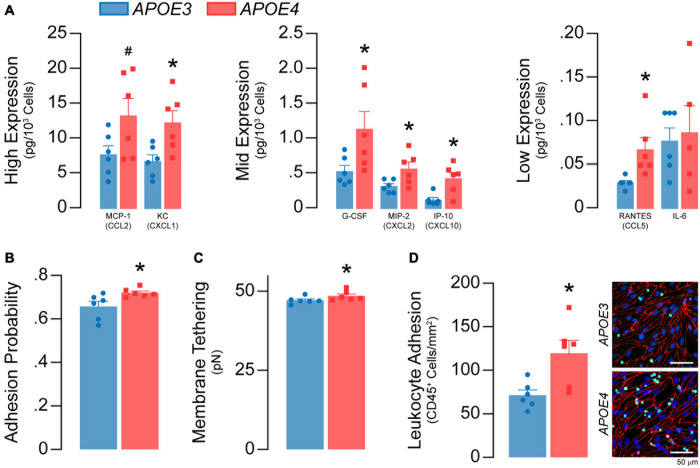
*APOE4* brain endothelial cells have a greater inflammatory phenotype under basal conditions. **(A)** The media of *APOE4* brain endothelial cells has higher levels of MCP-1/CCL2, KC/CXCL1, G-CSF, MIP-2/CXCL2, IP-10/CXCL10, RANTES/CCL5 and IL-6 when assessed by multiplex ELISA. **(B)** Higher inflammation can lead to greater immune cell adhesion that involves selectin-mediated capture. Selectin-mediated binding can be evaluated via atomic force microscopy, using cantilevers coated/biofunctionalized with the selectin ligand sialylic-Lewis^*x*^ (sLe^*x*^). In this assay, the biofunctionalized cantilever tip touches and indents the cell, then retracts; binding of sLe^*x*^ to selectins on the plasma membrane results in a rupture event when the cantilever is retracted (Askarova et al., 2013). Thus, two parameters are obtained; adhesion probability (i.e., whether a rupture occurs and, therefore, selectin binding) and selectin-mediated membrane tethering force (i.e., the force needed to rupture the tether). In *APOE4* brain endothelial cells there is higher sLe^*x*^-mediated adhesion probability and **(C)** membrane tethering force (analyzed by paired *t*-test). Immune cell adhesion can be directly assessed using a leukocyte adhesion assay. In this assay, unstimulated leukocytes (white blood cells) isolated from the spleens of *APOE3*-targeted replacement mice are spiked into the media of brain endothelial cell cultures. After 60 minutes, loosely adherent and unbound cells are removed and the remaining, firmly adherent cells are quantified by immunocytochemistry (CD45) (Lutz et al., 2017). **(D)** Consistent with AFM data, there are more CD45^+^ leukocytes adhered to *APOE4* brain endothelial cells. Data is expressed as mean ± S.E.M. *p < 0.05 by Student’s *t*-test with *n* = 6.

**FIGURE 9:**
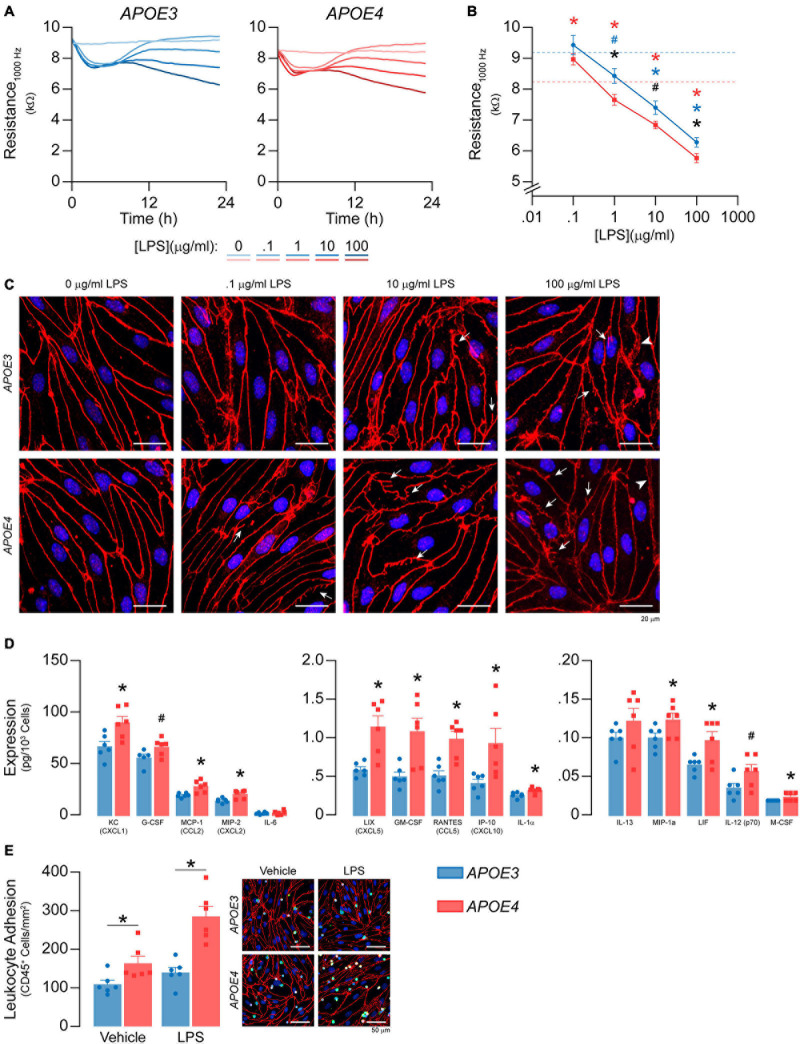
*APOE* modulates inflammation after LPS treatment. **(A,B)** LPS was spiked into the media to a final concentration of 100 ng/ml – 100 μg/ml and TEER was measured over the course of 24 h at 1000Hz. LPS lowers TEER at 1, 10, and 1000 μg/ml in both *APOE3* and *APOE4* brain endothelial cells and at these doses, TEER values are higher with *APOE3* compared to *APOE4* (2-way ANOVA followed by Student’s *t*-test; *APOE3 n* = 5, *APOE4*, *n* = 6). Dashed line represents vehicle control for each genotype (i.e., blue is *APOE3* and red is *APOE4*). **(C)** Qualitatively, high doses of LPS also modulate the brain endothelial cell shape, with morphological changes that include abnormal tight junction protein protrusions (white arrows) and separation of tight junctions from neighboring cells (arrow heads). **(D)** After 24 h of treatment with 100 ng/ml LPS, there are higher levels of KC/CXCL1, G-CSF, MCP-1/CCL2, MIP-2/CXCL2, LIX/CXCL5, GM-CSF, RANTES/CCL5, IP-10/CXCL10, IL-1a, MIP-1a, LIF, IL-12 (p70), and M-CSF. **(E)** To determine whether *APOE* modulated immune cell adhesion after LPS treatment (100 ng/ml, 24 h), we modified the leukocyte adhesion assay. Leukocytes were added with a full media change, rather than spiking, to avoid activation by LPS. Therefore, any *APOE* genotype differences in chemokine and cytokine levels that typically influence immune cell adhesion would be negated during the assay. Nonetheless, there are higher numbers of CD45^+^ leukocytes adhered to *APOE4* brain endothelial cells both with (∼49%) and without (∼104%) LPS treatment. Data is expressed as mean ± S.E.M. *p < 0.05 by two-tailed Student’s *t*-test and ^#^*p* < 0.05 by one-tailed Student’s *t*-test; black is *APOE4* vs. *APOE3*, blue is *APOE3* + LPS vs. *APOE3* Vehicle, red is *APOE4* + LPS vs. *APOE4* Vehicle. *n* = 6 (unless otherwise specified above).

**FIGURE 10:**
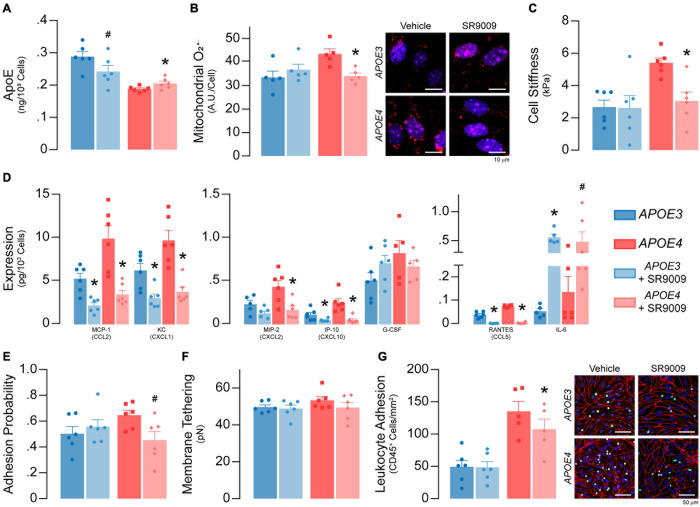
SR9009 treatment impacts metabolism and inflammation in *APOE4* brain endothelial cells. At confluence, brain endothelial cells were treated with 5 μM SR9009 for the 48 h leading up to each assay. **(A)** SR9009 treatment results in lower apoE levels (∼16%) with *APOE3* and higher levels (∼9%) with *APOE4* when assessed by ELISA. **(B)** SR9009-treated *APOE4*, but not *APOE3*, brain endothelial cells have lower mitochondrial superoxide levels compared to the vehicle (*n* = 5). **(C)** Cell stiffness is lower in SR9009-treated *APOE4*, but not in *APOE3*, brain endothelial cells (analyzed by paired *t*-test). **(D)** MCP-1/CCL2, KC/CXCL1, IP-10/CXCL10 and RANTES/CCL5 levels are lower with SR9009 in both *APOE3* and *APOE4* brain endothelial cells when assessed by multiplex ELISA. SR9009 lowers MIP-2 levels in *APOE4* brain endothelial cells and increases IL-6 levels for both *APOE3* and *APOE4* endothelial cells. **(E)** The adhesion probability is lower in *APOE4* brain endothelial cells with SR9009 treatment but **(F)** membrane tethering force is unaffected (analyzed by paired *t*-test). **(G)** Leukocyte adhesion is lower with *APOE4* following treatment with SR9009 (*APOE3 n* = 6, *APOE4 n* = 5). Data is expressed as mean ± S.E.M. *p < 0.05 by two-tailed Student’s *t*-test and ^#^*p* < 0.05 by one-tailed Student’s *t*-test compared to vehicle control with *n* = 6 (unless otherwise specified above).

**FIGURE 11:**
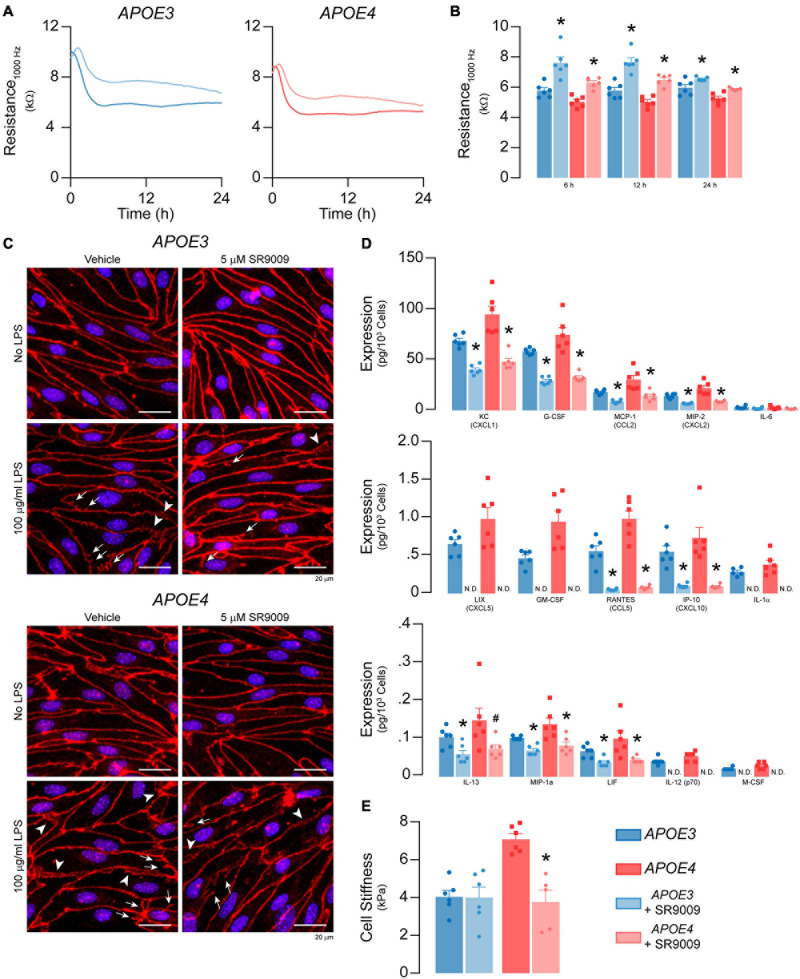
SR9009 mitigates the effects of LPS on metabolism and inflammation in *APOE4* brain endothelial cells. Confluent cells were treated with 5 μM SR9009 for 48 h and LPS was spiked into the media 24 h prior to assays. SR9009 treatment mitigated 100 μg/ml LPS-induced disruption of **(A,B)** paracellular permeability (*APOE3* vehicle *n* = 5, *APOE4* SR9009 *n* = 5, all other groups *n* = 6) and **(C)** changes in tight junction morphology. In cells treated with 100 ng/ml LPS, SR9009 treatment results in **(D)** lower levels of chemokines and cytokines for both *APOE3* and *APOE4* (multiplex ELISA) and **(E)** lower cell stiffness (analyzed by paired *t*-test, *n* = 5 for *APOE4* SR9009, all others *n* = 6), Data is expressed as mean ± S.E.M. *p < 0.05 by two-tailed Student’s *t*-test and ^#^*p* < 0.05 by one-tailed Student’s *t*-test compared to vehicle control with *n* = 6 (unless otherwise specified above).

In Figure 1, cell confluence was measured via capacitance and paracellular permeability by transendothelial electrical resistance (TEER). In addition, total cell counts (DAPI), proliferation (bromodeoxyuridine), and tight junction proteins were assessed by immunocytochemical analysis. ApoE levels were measured using *in situ* hybridization, immunocytochemistry, western blot, ELISA and native gel analysis (Figure 2 and Supplementary Figure 1). RNA-sequencing analysis was performed to evaluate transcriptomic profiles (Figure 3). In Figure 4, ATP levels, ATP production rates, percentage of ATP production due to glycolysis or oxidative phosphorylation (Seahorse ATP Rate Assay), glucose uptake rate and lactate production were measured. Mitochondrial activity was also evaluated using assays for membrane potential (tetramethylrhodamine ethyl ester, JC-1), citrate synthase activity, electron transport chain complex levels (western blot) and the ratio of NAD^+^: NADH. Figure 5 and Supplementary Figure 2 contain readouts for cellular reactive oxygen species (2′,7′–dichlorofluorescein diacetate, hydrogen peroxide, peroxynitrite, cellular superoxide anions), mitochondrial reactive oxygen species and calcium levels (MitoSOX, hydroxyl radical levels, Rhod-2), and mitophagy. Levels of antioxidants (heme and bilirubin levels, reduced:oxidized glutathione ratio) were also measured. Markers and outcomes of oxidative stress to DNA (8-oxo-dG and γH2A.X immunocytochemistry), proteins (glutathionylation, carbonylation measured by DNPH binding, autophagy via BacMan 2.0 RFP-GFP-LC3B transfection, chymotrypsin-, trypsin- and caspase-like proteasome activities), and lipids (peroxidation detected by BODIPY^®^ 581/591 C11, TBARS and 4*-*hydroxynonenal ELISA) were measured in Figure 6. Lipid homeostasis was determined using lipidomics, and by measuring phosphatidylcholine (ELISA), cholesterol and triglycerides levels, cell stiffness (atomic force microscopy) and extracellular lactate dehydrogenase levels (Figure 7 and Supplementary Figure 3). Basal inflammation was evaluated through assays for chemokine and cytokine levels in the media (Miliplex assay), selectin-mediated membrane tethering force and adhesion probability (atomic force microscopy with sialyl-Lewis^*x*^-coated cantilevers), and leukocyte adhesion (Figure 8). Further, after LPS treatment, the effect of *APOE* on TEER, apoE levels, chemokine and cytokine levels (Miliplex assay) and leukocyte adhesion were evaluated (Figure 9 and Supplementary Figure 4). The impact of the Rev-Erb agonist SR9009 (5 μM) on apoE levels (media, ELISA), mitochondrial superoxide levels (MitoSOX), cell stiffness (atomic force microscopy) and inflammation (chemokine and cytokine levels, selectin-mediated membrane tethering force and adhesion probability, and leukocyte adhesion) was determined (Figure 10). In addition, we assessed whether SR9009 modulated apoE levels (media, ELISA), TEER, chemokine and cytokine levels, cell stiffness, selectin-mediated membrane tethering force and adhesion probability, and leukocyte adhesion with LPS treatment (Figure 11 and Supplementary Figure 4).

### Data and Statistical Analysis

For all experiments, data were normalized to either cell count or protein concentration. In addition, for assays that required quantification of media samples, total volume of media was measured at the end of the experiment and incorporated into calculations (media volume × [analyte]/cell number). In every figure, n represents either an individual animal or data from separate isolations of grouped animals (as described in Supplementary Table 1). All data are presented as mean ± S.E.M and were analyzed using Student’s *t*-test or two-way ANOVA followed by the appropriate multiple comparisons test as described in the figure legends (with GraphPad Prism v9). Outliers were excluded by Grubbs’ test with α = 0.05. Supplementary File 2 contains all data used to generate the graphs in this manuscript and statistical comparisons tables. RNA-sequencing data is available from Gene Expression Omnibus; GSE160483.

## Results

### Confluence, Permeability, *ApoE* Production, and Transcriptomic Analysis

Our goal was to evaluate the role of human *APOE* genotype in modulating the phenotype of brain endothelial cells *in vitro*. Brain endothelial cells are highly specialized, and so we designed our study to limit phenotypic changes (e.g., protein expression, functions, loss of specialization) that can occur with cell passage and longer culture times. Therefore, we implemented an experimental protocol that enabled us to complete all experiments with fully confluent primary mouse brain endothelial cells expressing *APOE3* or *APOE4* at passage 0, within 7 days of isolation. Due to the novelty of the topic, our initial set of experiments were designed to evaluate the role of *APOE* in modulating general cellular characteristics, apoE production and the transcriptomic profile of brain endothelial cells.

#### *APOE3* and *APOE4* Brain Endothelial Cells Form Confluent Monolayers of Contact Inhibited Cells

We first determined whether *APOE* genotype modulated the ability of brain endothelial cells to form a confluent monolayer of contact-inhibited cells, as found *in vivo*. *APOE3* and *APOE4* brain endothelial cells both established a monolayer 3 days after isolation (Figure 1A). 7 days post-isolation, cultures of both *APOE* genotypes averaged 8 × 10^4^ cells/cm^2^ (Figure 1B) and displayed minimal bromodeoxyuridine staining (<5%, Figure 1C), consistent with contact-inhibited endothelial cells. A second important feature of brain endothelial cells is the development of a paracellular barrier, due to tight junctions (Figure 1D), which we assessed using transendothelial electrical resistance (TEER). TEER values were ∼7-10% lower in *APOE4* brain endothelial cells (7 days post-isolation, Figure 1E, *p* = 0.0045). These data demonstrate that *APOE* genotype does not modulate proliferation or cell number at confluence, however TEER is slightly lower in *APOE4* brain endothelial cells.

#### *APOE4* Brain Endothelial Cells Produce Less *ApoE*

For the *APOE* genotype of brain endothelial cells to modulate their function, they would have to produce apoE. We found that *APOE3* and *APOE4* brain endothelial cells express *APOE* by *in situ* hybridization (Figure 2A) and confirmed the presence of cell-associated apoE at the protein level by immunocytochemistry (Figure 2B). We next determined if *APOE* modulated apoE protein levels (Figures 2C-E). Cell-associated monomeric apoE was detected as a doublet (34 and 36 kDa) and was lower in *APOE4* brain endothelial cell lysates when measured by western blot analysis (34 kDa, *p* = 0.036; 36 kDa, *p* = 0.0064, Figure 2C). Consistent with cell-associated apoE data, secreted/extracellular monomeric apoE levels (in media) were lower with *APOE4* (34 kDa, *p* = 0.0008; 36 kDa, *p* = 0.0014), as was a multimeric apoE band (∼90-100 kDa, *p* = 0.014) when assessed by western blot analysis (Figure 2D) and extracellular apoE levels were approximately 15% lower with *APOE4* when quantified by ELISA (Figure 2E, *p* < 0.0001). Evidence suggests that cell-derived apoE4 is less lipidated than apoE3 when produced by other cell types, but we found no qualitative differences in the migration of apoE produced by *APOE3* and *APOE4* brain endothelial cells in native gel analysis (Supplementary Figure 1). However, apoE levels were lower in the media from *APOE4* brain endothelial cells when analyzed under native conditions. Collectively, our data demonstrate that the *APOE* genotype modulates the production of apoE by brain endothelial cells (i.e., *APOE3* > *APOE4*).

#### *APOE* Genotype Modulates the Transcriptomic Profile of Brain Endothelial Cells

To identify the potential impact of *APOE* genotype on brain endothelial cell biology, we conducted an unbiased RNA sequencing approach (Figure 3A). There were 1304 differentially expressed genes in *APOE4* brain endothelial cells that fell into both broad (e.g., molecular mechanisms of cancer) and highly specific (e.g., sperm motility) categories using canonical pathway analysis (Supplementary File 2). Our manual comparative analysis using several resources (UniProtKB, GeneCards, Pubmed) suggested that *APOE* modulates metabolism, inflammation, signaling, cell cycle/differentiation, ion homeostasis, cytoskeleton, cell adhesion, as well as other general categories including transcription/translation (Figure 3B, Supplementary Table 2, and Supplementary File 2). We focused our subsequent research on metabolism and inflammation as there were many transcripts in these categories, they are essential cellular processes, are often interconnected, and are found disrupted in diseases with vascular dysfunction.

### Metabolism

*APOE* modulated gene expression profiles for different aspects of metabolism related to energy production, mitochondrial function, reactive oxygen species levels and oxidative stress (Supplementary Table 2); findings that served as a segue to our functional assays.

#### *APOE4* Brain Endothelial Cells Have Lower Glycolysis and Higher Oxidative Phosphorylation and Mitochondrial Activity Compared to *APOE3*

Endothelial cell metabolism is important in homeostatic conditions and vascular disorders (Dranka et al., 2010; Tang et al., 2014; Bierhansl et al., 2017; Pi et al., 2018). Our transcriptomics data (Supplementary Table 2) implied that *APOE* genotype altered the amount of glycolysis compared to oxidative phosphorylation. Therefore, we evaluated the role of *APOE* in modulating ATP levels, glycolysis and oxidative phosphorylation in brain endothelial cells.

We found no differences in total ATP levels between *APOE* genotypes (Figure 4A) or rates of ATP production (Figure 4B). However, the proportion of ATP produced by glycolysis was lower in *APOE4* brain endothelial cells compared to *APOE3* (∼15%, *p* = 0.038, Figure 4C), as were both the rate of glucose uptake (∼25%, *p* = 0.001, Figure 4D) and lactate levels in the media (∼12%, *p* = 0.046, Figure 4E). These data support that, compared to *APOE3*, *APOE4* brain endothelial cells have higher rates of mitochondrial oxidative phosphorylation compared to glycolysis to produce ATP (Figure 4C). Consistent with this idea, markers of mitochondrial activity were higher in *APOE4* brain endothelial cells including lower membrane potential (∼50%, TMRE, *p* = 0.028; JC-1, *p* = 0.013, Figures 4F,G), higher citrate synthase activity (∼20%, *p* = 0.0045, Figure 4H), greater levels of electron transport chain components (transcripts and proteins, Figure 4I and Supplementary Table 2) and a higher NAD^+^: NADH ratio (NAD/NADH-Glo assay in which a reductase reduces proluciferin to luciferin in the presence of NADH, *p* = 0.016, Figure 4J). Overall, our data support that there is higher oxidative phosphorylation and mitochondrial activity in *APOE4* brain endothelial cells.

#### Higher Mitochondrial Superoxide Levels and Lower Antioxidant Levels With *APOE4*

Mitochondrial activity produces reactive oxygen species, which play several important physiological roles including metabolic adaptation, signaling and stress/inflammatory responses. As there was higher mitochondrial activity in *APOE4* brain endothelial cells, we evaluated whether there was a corresponding increase in reactive oxygen species levels. Surprisingly, total cellular reactive oxygen species levels were ∼32% lower (*p* = 0.0051, Figure 5A) with *APOE4*, which was likely driven by low H_2_O_2_ levels (*p* = 0.012, Figure 5B). Levels of total reactive oxygen species were also lower in *APOE4* brain endothelial cells after the addition of exogenous H_2_O_2_ (*p* = 0.036, Figure 5C). These data suggest upregulation of H_2_O_2_ degradation pathways with *APOE4*. For example, peroxisome activity may be higher due greater oxidative phosphorylation, and we found transcript levels of two H_2_O_2_ degradation enzymes (Gpx7, Prdx4) were higher in *APOE4* brain endothelial cells (Supplementary Table 2). For other common reactive oxygen species, although there were no changes in peroxynitrite levels (Figure 5D), cellular superoxide (O_2_^–^) levels were ∼31% higher (*p* = 0.045, Figure 5E) in *APOE4* brain endothelial cells. O_2_^–^ is more proximally linked to mitochondrial activity than other reactive oxygen species and when evaluated directly in mitochondria, there were ∼29% higher O_2_^–^ (*p* = 0.0036, Figure 5F) and ∼7% higher hydroxyl radical (*p* = 0.02, Figure 5G) levels with *APOE4*, without changes in mitochondrial calcium level or mitophagy (Supplementary Figure 2). Therefore, with *APOE4*, higher mitochondrial activity may have resulted in greater levels of O_2_^–^.

Levels of reactive oxygen species are intimately linked to antioxidants; transcripts related to two important antioxidant systems, heme and glutathione, were modulated by *APOE* genotype (Supplementary Table 2) and we measured markers of both. Heme is produced in a series of reactions involving the mitochondria and can be degraded into the antioxidant and anti-inflammatory molecule bilirubin. In *APOE4* brain endothelial cells, there were lower levels of genes that both produce (Ppox, Alas) and degrade heme to bilirubin (Hmox1, Blvrb) and, importantly, lower levels of intracellular heme (*p* = 0.001, Figure 5H) and bilirubin (*p* = 0.0038, Figure 5I). Glutathione (GSH) neutralizes reactive oxygen species and is converted to oxidized/disulfide glutathione (GSSG); low GSH:GSSG indicates higher reactive oxygen species levels. In *APOE4* brain endothelial cells the GSH:GSSG ratio was ∼ 32% lower (*p* = 0.0002, Figure 5J). Relatedly, we also found lower levels of reduced nicotinamide adenine dinucleotide phosphate with *APOE4*, which can reduce GSSG to form GSH (Supplementary File 2). Collectively our data demonstrate an altered balance of reactive oxygen species, characterized by higher mitochondrial O_2_^–^ and hydroxyl radicals, lower heme/bilirubin levels, and a lower GSH:GSSG ratio in *APOE4* brain endothelial cells.

#### Higher Markers of Oxidative Stress in *APOE4* Brain Endothelial Cells

Reactive oxygen species can induce cellular damage to DNA, proteins, and lipids, often termed oxidative stress. As levels of O_2_^–^ were higher in *APOE4* brain endothelial cells, we determined whether markers of oxidative stress were also higher. Although there were no *APOE* genotype effects on markers of DNA damage (Figures 6A,B), there were alterations in markers of oxidative stress to proteins. With *APOE4* we found ∼50% lower protein glutathionylation (*p* = 0.0007, Figure 6C), which is a protective mechanism to limit oxidative damage to cysteine thiol groups on proteins, and ∼25% higher protein carbonylation, which is an irreversible oxidation of amino acid side chains (*p* = 0.045, Figure 6D). Although we did not observe any changes in autophagy (Figure 6E), there were higher levels of a proteasomal 20S subunit (Psmb9, Supplementary Table 2) as well as higher chemotryspin-like (∼19%, *p* = 0.028) and caspase-like (∼14%, *p* = 0.034) proteasome activity (Figure 6F) with *APOE4*. The higher proteasome activity may reflect higher protein clearance due to oxidative stress and/or a physiological upregulation due to different cellular requirements in *APOE4* brain endothelial cells.

Lipids are sensitive to the effects of reactive oxygen species through peroxidation. We found that lipid peroxidation of an exogenously added sensor (BODIPY^®^ 581/591 C11) was higher with *APOE4* (∼ 20%, *p* = 0.046, Figure 6G), as were levels of TBA reactive substances (∼11% higher, *p* = 0.045, Figure 6H), although 4*-*hydroxynonenal levels were ∼11% lower (*p* = 0.011, Figure 6I). Often, changes in cellular lipid biology are reflected in membrane structures (e.g., plasma membrane), and in *APOE4* brain endothelial cells there were changes in transcripts related to membrane dynamics/composition (Supplementary Table 2). In fact, *APOE* genotype modulated metabolites related to membrane phospholipids when evaluated by lipidomic analysis, including lower levels of those related to phosphatidylcholine, phosphatidylethanolamine, and plasmalogens, whereas some ceramides were higher (Figure 7A and Supplementary File 2). Consistent with metabolomic data, phosphatidylcholine levels were ∼37% lower in *APOE4* brain endothelial cells (*p* = 0.0062, Figure 7B) and, while there were no changes in cholesterol levels (Figure 7C), total (∼45%, *p* = 0.0007) and free (∼26%, *p* = 0.0004) triglyceride levels were lower with *APOE4* (Figure 7D, also found in the media; Supplementary Figure 3). Changes in plasma membrane composition can alter mechanical properties of a cell including overall cell stiffness and integrity. We found that *APOE4* brain endothelial cells were ∼17% stiffer when measured by atomic force microscopy (*p* = 0.0002, Figure 7E). In addition, media levels of lactate dehydrogenase (LDH), a cytosolic enzyme found extracellularly when the plasma membrane is damaged, were ∼21% higher with *APOE4* (*p* = 0.0036, Figure 7F). Overall, our data support that there is oxidative damage to proteins (lower glutathionylation and higher carbonylation) and lipids (altered membrane composition, higher cell stiffness and permeability of the plasma membrane to LDH) in *APOE4* brain endothelial cells.

### Inflammation

There is a tight connection between metabolism and inflammation, and brain endothelial cells are the interface between plasma and brain inflammatory signaling. *APOE4* is associated with a different inflammatory response compared to *APOE3* in non-brain endothelial cells such as astrocytes and microglia (reviewed in Tai et al., 2015), and in endothelial-like cells differentiated from IPSCs (Rieker et al., 2019). In *APOE4* brain endothelial cells, there were higher levels of transcripts related to chemokines and pro-inflammatory cytokines, toll-like receptor signaling, immune cell recruitment/activation and MHC molecules, inflammasome signaling, and antiviral responses (leukocyte adhesion/immune cell activation and blood coagulation/clotting; Supplementary Table 2). Based on these transcriptomics data, we conducted further functional assays to determine the extent *APOE* modulates the inflammatory phenotype of brain endothelial cells.

#### *APOE* Modulates Basal Inflammation in Brain Endothelial Cells Characterized by Higher Chemokine Levels and Immune Cell Adhesion With *APOE4*

Soluble chemokines and cytokines are major effector molecules of the inflammatory response and can be produced by brain endothelial cells. Nonetheless, our finding that *APOE* genotype modulates chemokine/cytokine transcripts in brain endothelial cells was still surprising. Thus, we evaluated whether levels of 31 common chemokines and cytokines in the media were different between *APOE3* and *APOE4* brain endothelial cells by multiplex ELISA. Seven chemokines and cytokines were above the limit of detection, 6 of which were higher with *APOE4*: MCP-1/CCL2 (∼71%), KC/CXCL1 (∼81%), G-CSF (∼112%), MIP-2/CXCL2 (∼77%), IP-10/CXCL10 (∼236%), and RANTES/CCL5 (∼135%) (Figure 8A). One consequence of higher inflammation is the attachment of immune cells to brain endothelial cells, which is initiated by selectin-mediated capture. Selectin-mediated binding was higher with *APOE4* when assessed by atomic force microscopy using cantilevers coated/biofunctionalized with the selectin ligand sialylic-Lewis^*x*^ (sLe^*x*^) (Askarova et al., 2013) (*p* = 0.02, Figures 8B,C). Further, ∼68% more exogenously added CD45^+^ leukocytes adhered to *APOE4* brain endothelial cells (*p* = 0.013, Figure 8D) (Lutz et al., 2017). Our data demonstrate that *APOE4* brain endothelial cells have a higher basal inflammatory state characterized by higher chemokine/cytokine levels and immune cell adhesion.

#### *APOE* Modulates Inflammation After LPS Treatment, Characterized by Lower TEER, Higher Chemokine Levels and Immune Cell Adhesion With *APOE4*

*APOE* genotype-specific differences in inflammatory markers are particularly prominent after stimulation with an inflammatory agent (Tai et al., 2015). LPS, a bacterial endotoxin and toll-like receptor 4 agonist, is often utilized to induce inflammation *in vitro*, *in vivo* and even in human studies focused on *APOE* genotype. Therefore, we evaluated whether *APOE* modulated LPS-induced inflammation.

LPS has been reported to induce barrier disruption to brain endothelial cells, which may be modulated by *APOE* genotype. Therefore, we spiked LPS into the media at concentrations previously reported to modulate brain endothelial cell function, 100 ng/ml – 100 μg/ml (Nagyoszi et al., 2010; Kacimi et al., 2011; Li et al., 2012; Zhao et al., 2014; Qin et al., 2015; Serizawa et al., 2015), and measured TEER over the course of 24 h at 1000 Hz (Figures 9A,B). For both *APOE* genotypes LPS lowered TEER in an initial phase (0-6 h) followed by a plateau or a recovery (6-12 h) and, finally, a second phase of TEER decline (12-24 h). LPS induced dose-dependent effects on TEER at 12–24 h. For example, TEER values were ∼10, ∼20, and ∼30% lower compared to vehicle with 1, 10 and 1000 μg/ml LPS at 24 h for *APOE4* brain endothelial cells. Importantly, the higher TEER values we found with *APOE3* compared to *APOE4* at baseline (Figure 1E) were sustained with LPS treatment (Figure 9B). Indeed, when plotted as the log of the LPS dose response curves at 24 h, there is an apparent leftward curve shift with *APOE4* compared to *APOE3*. High doses of LPS also modulated brain endothelial cell shape, with morphological changes that included abnormal tight junction protein protrusions and separation of tight junctions from neighboring brain endothelial cells (Figure 9C). Interestingly, apoE levels were lower in brain endothelial cell cultures treated with 10 and 100 μg/ml LPS for both genotypes (Supplementary Figure 4A).

We next evaluated whether *APOE* modulated chemokine and cytokine levels after treatment with 100 ng/ml LPS (24 h). This dose and time-point were selected to avoid the *in vitro* equivalent of pathological changes in paracellular permeability and so that we could also evaluate immune cell adhesion. After LPS treatment, fifteen chemokines and cytokines were above the limit of detection (Figure 9D), 13 of which were higher with *APOE4* by ∼ 20-30% (G-CSF, IL-1α, MIP-1α/CCL3, M-CSF/CSF1, KC/CXCL1), 40-50% (MCP1/CCL2, MIP-2/CXCL2, IL-12p70, LIF), and 100% (LIX/CXCL5, GM-CSF, RANTES/CCL5, IP-10/CXCL10). There were also more adhered CD45^+^ leukocytes in *APOE4* brain endothelial cells after LPS treatment (*p* = 0.0278, Figure 9E). These data demonstrate that, as for basal inflammation, after LPS treatment (100 ng/ml) there are higher chemokine/cytokine levels and immune cell adhesion with *APOE4*.

### Rev-Erb Is a Potential Pathway Modulated by *APOE* Genotype

Our final goal was to use pharmacological probes to identify pathways that could contribute to the *APOE4* brain endothelial phenotype. Through the evaluation of our RNA-sequencing data, we identified several signaling-related transcriptomic profiles as candidates, of which the nuclear receptor family was particularly prominent (Supplementary Table 2). There are different subclasses of nuclear receptors that hetero- and homodimerize, and agonists of RXR, LXR and PPAR nuclear receptor families can mitigate some dysfunctional changes found with *APOE4* (Koster et al., 2017; Moutinho et al., 2019). In *APOE4* brain endothelial cells there were lower levels of genes for RAR (e.g., Rarg, Rara) and LXR (Nr1h2) nuclear receptors, as well as nuclear receptor coactivators (e.g., Ncoa2, Ncoa7) and transcripts associated with general nuclear receptor activation (e.g., Klf15 Klf2, Klf4). Although these and other signal molecules warrant follow-up in future studies, we focused on the nuclear receptor Rev-Erb due to several considerations. The first was that transcript levels of both forms of the receptor (Rev-Erbα and β) were lower in *APOE4* brain endothelial cells and to greater extent than other nuclear receptors (Supplementary Table 2). Second is that molecules and transcripts related to Rev-Erb were also lower in *APOE4* brain endothelial cells. Although considered an orphan receptor, heme is an agonist of Rev-Erb which was lower in *APOE4* brain endothelial cells (Figure 5H) and Rev-Erb also regulates circadian genes, some of which were also lower (Per1, Per2, Per3, Supplementary Table 2) with *APOE4*. In addition, Rev-Erb is functionally linked to metabolism and inflammation. We, therefore, determined the effect of SR9009 (Stenabolic), a widely used Rev-Erb agonist (Solt et al., 2012), on select assays that were affected by *APOE4* both under basal conditions (Figure 10) and following stimulation with LPS (Figure 11).

#### SR9009 Treatment Impacts Metabolism and Inflammation Under Basal Conditions in *APOE4* Brain Endothelial Cells

We determined whether SR9009 influenced apoE levels, metabolism (mitochondrial O_2_^–^, cell stiffness) and inflammation (chemokine levels and leukocyte adhesion) under basal conditions. SR9009 treatment (5 μM, 48 h) resulted in lower apoE levels (∼16%) with *APOE3* and higher levels (∼9%) with *APOE4* when assessed by ELISA (Figure 10A). For metabolism, compared to vehicle, SR9009 treatment resulted in ∼18% lower mitochondrial O_2_^–^ levels (*p* = 0.0056, Figure 10B) and ∼44% lower cell stiffness compared to vehicle in *APOE4* brain endothelial cells (*p* = 0.01, Figure 10C). For inflammation, in both *APOE* genotypes SR9009 reduced chemokine levels by ∼50-80% (MCP-1/CCL2, KC/CXCL1, MIP-2/CXCL2, IP-10/CXCL10, RANTES/CCL5) compared to vehicle controls (Figure 10D). By contrast IL-6 levels were markedly increased by SR9009 (∼910% higher in *APOE3*, ∼259% higher in *APOE4*). SR9009 also lowered selectin-mediated binding (∼18%; Figure 10E) and leukocyte adhesion (21%, *p* = 0.0002, Figure 10G) in *APOE4* brain endothelial cells. Thus, SR9009 treatment lowers mitochondrial O_2_^–^, cell stiffness and inflammation in *APOE4* brain endothelial cells in basal conditions.

#### SR9009 Modulates LPS-Induced Effects on TEER and Inflammation in *APOE4* Brain Endothelial Cells

We evaluated whether SR9009 mitigated LPS-induced effects on TEER, cytokine/chemokine levels, cell stiffness, selectin binding and leukocyte adhesion. The extent of LPS-induced TEER disruption was lower with SR9009 treatment (100 μg/ml LPS, 24 h, Figures 11A,B). For both *APOE3* and *APOE4* brain endothelial cells, TEER was higher with SR9009 treatment at 6 h (∼32% higher *APOE3, p* = 0.0016, and ∼25% *APOE4, p* < 0.0001), 12 h (∼33% higher *APOE3 p* = 0.0003 and ∼29% *APOE4, p* < 0.0001), and 24 h (∼10% higher *APOE3, p* < 0.033 and ∼12% *APOE4, p* = 0.0037) compared to vehicle. Consistent with these findings, there were, qualitatively, fewer tight junction protrusions and breaks with SR9009 treatment (Figure 11C). The beneficial effects of SR9009 on TEER occurred without preventing the LPS-induced (100 μg/ml) lowering of apoE levels in brain endothelial cells. In fact, SR9009 resulted in ∼15% lower apoE levels compared to vehicle after LPS treatment (100 μg/ml) for both *APOE3* and *APOE4* (Supplementary Figure 4B). SR9009 treatment also resulted in lower chemokine/cytokine levels after 24 h incubation with 100 ng/ml LPS for both *APOE3* and *APOE4* brain endothelial cells ranging from ∼40-50% (KC/CXCL1, G-CSF, MCP-1/CCL2, MIP-2/CXCL2, IL-13, LIF) to 80-100% (LIX/CXCL5, RANTES/CCL5, IP-10/CXCL10, IL-1α, IL-12p70, M-CSF/CSF1) (Figure 11D). However, in contrast to baseline conditions, SR9009 had no effect on selectin-mediated adhesion, and increased leukocyte adhesion in *APOE4* brain endothelial cells (∼38%, Supplementary Figures 4C-E) after LPS treatment (100 ng/ml). SR9009 treatment also resulted in lower cell stiffness in *APOE4* brain endothelial cells (∼47%, 24 h after 100 ng/ml LPS, *p* < 0.018 Figure 11E). Thus, SR9009 partially mitigates high-dose LPS-induced disruption of TEER and lowers chemokine levels and cell stiffness with a low-dose of LPS in *APOE4* brain endothelial cells.

## Discussion

In this manuscript, we identified that *APOE4* is associated with an altered brain endothelial cell phenotype characterized by differences in apoE levels, metabolism, and inflammation compared to *APOE3*. Further research could provide insight into the contribution of this phenotype to neurovascular dysfunction in aging and neurodegenerative disorders.

### Brain Endothelial Cell *APOE* Genotype and Neurovascular Function

Reports of *APOE4*-associated neurovascular dysfunction in neurodegenerative disorders (reviewed in Zlokovic, 2013; Tai et al., 2016) has led to ongoing research to identify underlying mechanisms. ApoE has been found to impact brain endothelial cells indirectly and directly. Indirectly, *APOE* modifies peripheral inflammation, neuroinflammation, metabolites, and disease-specific proteins (e.g., Aβ), all of which can affect brain endothelial cell function (Bell et al., 2012; Tai et al., 2016; Yamazaki et al., 2020a). ApoE produced by pericytes (Yamazaki et al., 2020b) and astrocytes (Nishitsuji et al., 2011) can directly signal to brain endothelial cells *in vitro* and our data supports autocrine effects of apoE in brain endothelial cells. The complex regulation of brain endothelial cell function by apoE provides an opportunity for context-dependent integration. For example, signals from different cell types, over a range of distances from the brain and periphery, in response to homeostatic and stress/pathological conditions can all be transmitted to brain endothelial cells for the control of neurovascular function. Within this framework, apoE production by brain endothelial cells may be important for local signaling. Brain endothelial cells receive signaling inputs from both the interstitial fluid and blood, and therefore an added advantage of autocrine apoE production is the ability to self-regulate, rather than rely on apoE produced by other cell types.

### Integrated Working Model of the *APOE* Modulated Brain Endothelial Cell Phenotype

**FIGURE 12:**
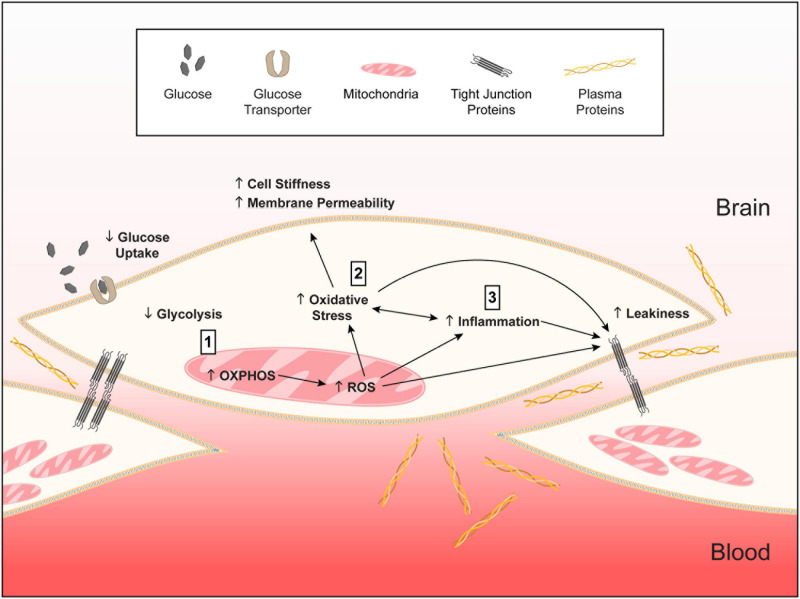
Working model of *APOE*-modulated brain endothelial cell function. **↓** = lower and **↑** = higher for *APOE4* compared to *APOE3*. We propose that **(1)** *APOE4* brain endothelial cells have higher preference for oxidative phosphorylation (OXPHOS) compared to glycolysis. Lower glycolysis is evident in ATP rate assays, lower rate of glucose uptake and higher lactate production. Due to OXPHOS there is higher peroxisome and mitochondrial activity indicated by higher hydrogen peroxide degradation, mitochondrial membrane potential, citrate synthase activity and levels of electron transport chain complexes. Higher mitochondrial activity leads to the generation of reactive oxygen species, particularly superoxide, and lower levels of antioxidant systems, including heme and glutathione. **(2)** Higher reactive oxygen species lead to more oxidative stress to proteins (e.g., carbonylation) that must be cleared by proteasome activity. Oxidative stress to lipids (peroxidation, production of TBARS) alters membrane structures (lower phospholipids, higher ceramides) that manifests as higher cell stiffness and plasma membrane permeability. **(3)** Either in parallel or due to higher mitochondrial activity, inflammation pathways are activated with *APOE4*. *APOE4*-assocaited inflammation is characterized by chemokine production, immune cell adhesion and higher sensitivity of innate receptors to activation (e.g., TLR4). The combination of all these changes leads to higher basal transcellular permeability with *APOE4*. Therefore, autocrine signaling of apoE in brain endothelial cells represents a novel cellular mechanism for how *APOE* regulates neurovascular function. We further propose that this pathway is not necessarily detrimental in basal conditions for *APOE4* and may even be beneficial for responding to infections and other stressors earlier in life. However, chronic changes in metabolic, mitochondrial, or inflammatory pathways in neurodegenerative conditions could lead to brain endothelial cell dysfunction.

Based on our data and findings in other cell types (Mahley et al., 2007; Liu et al., 2013; Dose et al., 2016; Fernandez et al., 2019; Butterfield and Mattson, 2020; Flowers and Rebeck, 2020; Johnson, 2020), we present a working model of how *APOE* genotype could modulate the basal phenotypic state of brain endothelial cells (Figure 12). In this model, we propose that, compared to *APOE3*, *APOE4* brain endothelial cells have higher preference for oxidative phosphorylation over glycolysis, which results in higher mitochondrial activity and generation of mitochondrial reactive oxygen species and lower levels of antioxidants (heme/bilirubin and glutathione). Higher levels of reactive oxygen species with *APOE4* produce oxidative stress to proteins that must be cleared by proteasome activity, and lipids, which results in greater cell stiffness and plasma membrane permeability. In tandem, or due to higher mitochondrial activity, there is higher inflammation with *APOE4* characterized by chemokine production, immune cell adhesion and higher sensitivity of innate receptors to activation (e.g., TLR4). The combination of all these changes leads to higher basal transcellular permeability with *APOE4*.

The *APOE4*-associated brain endothelial cell phenotype is not necessarily detrimental in basal conditions. Indeed, *APOE4* carriers do not have developmental neurovascular malformations, or overt vascular dysfunction early in life. One could even argue that compared to *APOE3*, *APOE4* brain endothelial cells would respond to infections and other stressors earlier in life in a more beneficial manner. However, chronic changes in neurodegenerative conditions could lead to brain endothelial cell dysfunction. For example, risk factors for dementia are associated with metabolic changes, oxidative stress, peripheral inflammation and neuroinflammation, all of which could exacerbate the *APOE4*-associated brain endothelial cell phenotype as found in other endothelial cell contexts (Sena et al., 2018; Urbano et al., 2019).

### Signaling Pathways and *APOE*-Modulated Brain Endothelial Cell Function

There are several potential mechanisms of how *APOE* modulates the brain endothelial cell phenotype. Overall, answers to the question of how a single amino acid difference between apoE3 (cysteine at 112) and apoE4 (arginine at 112) results in functional changes are proving complex, at times enigmatic, and continue to be the focus of several research groups (reviewed in Mahley et al., 2007; Liu et al., 2013; Flowers and Rebeck, 2020). One aspect of this question is whether there are differences in structural properties of apoE. ApoE is post-translationally lipidated prior to secretion, and one suggestion is that apoE4-containing lipoproteins are less lipidated than apoE3-containing lipoproteins, resulting in lower stability and levels. In our study, we found that transcript levels of *APOE4* were lower than *APOE3* (-0.56 log-fold change), as were apoE4 levels. Thus, one explanation for these data is that in brain endothelial cells, the lower stability of apoE4 results in lower levels that in turn further results in a cascade that suppresses the transcription of *APOE*. In tandem, there may also be proteasomal degradation of apoE4, further contributing to modulation of apoE levels. The isoform differences in structure and lipidation are thought to influence a range of fundamental processes even if apoE levels were equivalent. There are many proposed consequences of the lower levels and structural differences of apoE4 including lower ability to maintain cholesterol and lipid homeostasis, less binding to oxidative stress-related products and other substrates, disruption in adaptor molecule function, modulation of intracellular metabolism and organelle dynamics, altered activation and recycling of the apoE receptors and a profound influence on intracellular signaling cascades. It remains plausible that any of these are proximally linked to *APOE*-associated brain endothelial cell phenotypic differences.

Intertwined with the structural and functional differences between the apoE isoforms is cellular signaling. As indicated in our transcriptomics data, several signaling cascades were differentially modulated by *APOE* (Supplementary File 2), many which are related to metabolism and inflammation, providing a link with our proposed working model (Figure 12). Given that multiple aspects of cellular biology were modulated by *APOE4* genotype, there is high likelihood of a complex interaction among the different signaling pathways; it is unlikely that a single signaling pathway is responsible for all the *APOE*-modulated functional differences. However, in general, there is increasing evidence of a connection between *APOE* genotype and nuclear receptors (reviewed in Koster et al., 2017; Moutinho et al., 2019). For example, agonists for PPAR, LXR and RXR have been shown to modulate either levels and lipidation of apoE and/or *APOE*-modulated inflammation, metabolism, neuronal function, and behavior. The precise mechanistic connection between *APOE* and nuclear receptors is unclear but may be related to the *APOE*-associated phenotype of a cell. For example, higher inflammation and fatty acid oxidation with *APOE4* would be associated with a corresponding set of signaling pathways that could include lower activation of nuclear receptors that suppress these functions.

Our data extends the link between *APOE* and nuclear receptors to Rev-Erb since, with *APOE4*, there were lower levels of Rev-Erbα and β transcripts, heme (Rev-Erb agonist) and circadian genes (Per1, Per2, Per3). Furthermore, SR9009 (Rev-Erb agonist) treatment resulted in improved metabolic and inflammatory phenotypes. To date, no direct link between *APOE* genotype and Rev-Erb has been reported, however there is overlap with the reported functions of Rev-Erb and the metabolic and inflammatory aspects of the *APOE4* brain endothelial phenotype (Raspe et al., 2002; Duez and Staels, 2008; Duez et al., 2008; Le Martelot et al., 2009; Yin et al., 2010; Bugge et al., 2012; Cho et al., 2012; Delezie et al., 2012; Gibbs et al., 2012; Woldt et al., 2013; Mayeuf-Louchart et al., 2017; Pariollaud et al., 2018; Wang et al., 2018; Reitz et al., 2019; Cunningham et al., 2020; Wang et al., 2020). In addition beneficial effects have been reported for SR9009 in a variety of *in vivo* models of disease with metabolic (Raspe et al., 2002; Le Martelot et al., 2009; Cho et al., 2012) and inflammatory components (Delezie et al., 2012). However, specifically for dementia, data are conflicted on whether SR9009 is beneficial or detrimental in Alzheimer’s disease-relevant models. On the one hand, the loss of Rev-Erb results in a mania-like phenotype and impaired performance in memory tasks (Mayeuf-Louchart et al., 2017) and, in a model of aging, SR9009 treatment improved and reversed behavioral deficits (Woldt et al., 2013). On the other hand, inhibition of Rev-Erb results in higher synaptic markers in an amyloidosis model (Bugge et al., 2012). In addition, SR9009 can act independently of Rev-erb (Duez et al., 2008), but the mechanisms have not yet been identified. Our own data, albeit *in vitro*, would suggest that, in the context of *APOE4*, agonists of Rev-Erb would result in a beneficial phenotype, particularly for the cerebrovasculature, through modulating metabolism and/or inflammation.

### Limitations and Future Directions

Our data provide important phenotypic information on the role of *APOE* genotype in brain endothelial cell function, however, identification of the underlying mechanistic pathways is important. In addition, the use of double and triple cultures of brain endothelial cells, astrocytes and pericytes could enable functional comparisons of the different sources of apoE. Although when evaluated by western blot analysis our brain endothelial cell cultures are GFAP- and desmin-negative, we recognize that a limitation of primary cell isolation, regardless of the cell type, is the presence of non-target cells and it is rare that any protocol produces completely pure cultures.

From a broader perspective, identifying the extent that brain endothelial cell *APOE* genotype impacts vascular function *in vivo*, as well as interactions with Rev-Erb signaling is important. We and others have demonstrated that markers of cerebrovascular leakiness are higher with *APOE4 in vivo* (Salloway et al., 2002; Zipser et al., 2007; Poels et al., 2010; Halliday et al., 2013; Zlokovic, 2013; Halliday et al., 2015; Tai et al., 2016; Thomas et al., 2016; Marottoli et al., 2017; Tai et al., 2017; Thomas et al., 2017), however the extent that metabolism and inflammatory markers are affected is unknown and are the focus of our ongoing studies. In terms of Rev-Erb, it is interesting to note that under basal conditions we found that SR9009 slightly increased apoE4 levels, which could have contributed to the modulation of select metabolic and inflammatory read-outs. Thus, Rev-ErB may alter levels of genes or proteins involved in apoE metabolism, as found for other nuclear receptor agonists. Alternatively, SR9009 altered cellular metabolism, in turn upregulating genes related to apoE metabolism. We focused on *APOE* functional effects and, therefore, conducting a more detailed study with SR9009 in *APOE4* brain endothelial cells *in vitro* could reveal the underlying mechanism(s) of action. Indeed, a limitation of the current study is a lack of full pharmacological characterization of SR9009, including dose-response evaluation and testing activity in Rev-Erb knock out cells. In addition, and the focus of our ongoing studies, evaluation of SR9009 activity *in vivo* would aid in understanding potential clinical relevance of this class of drugs for *APOE4* associated neurodegeneration. Although we propose that *APOE4* brain endothelial cells are more prone to stress-induced degeneration, beyond select read-outs with LPS, we did not fully explore this concept, which is the focus on our ongoing studies. For example, phenotypic changes we found with *APOE4* in cells from younger mice may be more pronounced in cells isolated from older mice.

The *APOE4* associated brain endothelial cell phenotype may contribute to overall cerebrovascular and neuronal dysfunction in neurodegenerative disorders including dementia. However, it is critical to conduct further research to fully explore this concept. One important aspect is identification of how brain endothelial cell dysfunction impacts neuronal function, and there are multiple potential pathways including altered homeostasis of nutrients, neurotransmitters, metabolites and ions in the interstitial fluid, inflammation, influx of plasma proteins and other peripheral molecules into the brain, as well as modulation of disease specific elements such as clearance of amyloid-β. Related, is evaluation of whether some neuronal populations are more sensitive to the effects of cerebrovascular dysfunction, either due to fundamental differences in neuronal biology or greater disruption of neuronal signaling in specific neurodegenerative conditions. In addition, neurodegenerative disorders involve altered function of multiple cell types including peripheral cells, pericytes, vascular smooth muscle cells, glia as well as neurons. Therefore, full evaluation of how our identified endothelial cell-specific phenotype impacts the function of these other cell types is important, as is the incorporation of *APOE2* genotype, which is protective for neurodegeneration. These types of questions are the focus of our future studies and will be enabled by the development of mouse models to conditionally knock-down *APOE2*, *APOE3* or *APOE4* in endothelial cells.

## Conclusion

The goal of this manuscript was to test the hypothesis that brain endothelial cells produce apoE to modulate their own phenotype. Our *in vitro* data support this hypothesis and therefore autocrine signaling of apoE in brain endothelial cells represents a novel cellular mechanism for how *APOE* regulates neurovascular function. In basal conditions, *APOE4* brain endothelial cells had altered metabolism consistent with greater oxidative phosphorylation and higher inflammation. The basal differences may predispose *APOE4* brain endothelial cells to dysfunction during aging and in different neurodegenerative disorders, especially as *APOE4* is linked to cognitive decline in aging and Alzheimer’s disease, and to poorer outcomes after stroke and brain trauma. Therefore, the autocrine effects of apoE4 on metabolism and inflammation in brain endothelial cells could provide the framework for understanding mechanisms of neurovascular dysfunction in neurodegeneration, and open avenues for the development of therapeutics that target brain endothelial cells.

## Data Availability Statement

The datasets presented in this study can be found in online repositories. The names of the repository/repositories and accession number(s) can be found below: Gene Expression Omnibus (GSE160483).

## Ethics Statement

The animal study was reviewed and approved by the University of Illinois at Chicago Institutional Animal Care and Use Committee.

## Author Contributions

LT and FM conceived the study, performed the experiments, and wrote the manuscript with SL and JL. TT and SL conducted the leukocyte adhesion assays. XG and JL conducted the AFM experiments. ZA, PK, and MM-C conducted the transcriptomics analysis. All authors contributed to the article and approved the submitted version.

## Conflict of Interest

The authors declare that the research was conducted in the absence of any commercial or financial relationships that could be construed as a potential conflict of interest.

## References

[B1] Askarova S. Sun Z. Sun G. Y. Meininger G. A. Lee J. C. (2013). Amyloid-beta peptide on sialyl-Lewis(X)-selectin-mediated membrane tether mechanics at the cerebral endothelial cell surface. *PLoS One* 8:e60972. 10.1371/journal.pone.0060972 23593361 PMC3625223

[B2] Basu S. K. Ho Y. K. Brown M. S. Bilheimer D. W. Anderson R. G. Goldstein J. L. (1982). Biochemical and genetic studies of the apoprotein E secreted by mouse macrophages and human monocytes. *J. Biol. Chem.* 257 9788–9795. 6286633

[B3] Bell R. D. Winkler E. A. Singh I. Sagare A. P. Deane R. Wu Z. (2012). Apolipoprotein E controls cerebrovascular integrity via cyclophilin A. *Nature* 485 512–516. 10.1038/nature11087 22622580 PMC4047116

[B4] Bierhansl L. Conradi L. C. Treps L. Dewerchin M. Carmeliet P. (2017). Central role of metabolism in endothelial cell function and vascular disease. *Physiology (Bethesda)* 32 126–140. 10.1152/physiol.00031.2016 28202623 PMC5337830

[B5] Bugge A. Feng D. Everett L. J. Briggs E. R. Mullican S. E. Wang F. (2012). Rev-erbalpha and Rev-erbbeta coordinately protect the circadian clock and normal metabolic function. *Genes Dev.* 26 657–667. 10.1101/gad.186858.112 22474260 PMC3323877

[B6] Butterfield D. A. Mattson M. P. (2020). Apolipoprotein E and oxidative stress in brain with relevance to Alzheimer’s disease. *Neurobiol. Dis.* 138:104795. 10.1016/j.nbd.2020.104795 32036033 PMC7085980

[B7] Chen Y. Strickland M. R. Soranno A. Holtzman D. M. (2020). Apolipoprotein E: structural insights and links to Alzheimer disease pathogenesis. *Neuron* 109 205–221. 10.1016/j.neuron.2020.10.008 33176118 PMC7931158

[B8] Cho H. Zhao X. Hatori M. Yu R. T. Barish G. D. Lam M. T. (2012). Regulation of circadian behaviour and metabolism by REV-ERB-alpha and REV-ERB-beta. *Nature* 485 123–127. 10.1038/nature11048 22460952 PMC3367514

[B9] Cunningham P. S. Meijer P. Nazgiewicz A. Anderson S. G. Borthwick L. A. Bagnall J. (2020). The circadian clock protein REVERBalpha inhibits pulmonary fibrosis development. *Proc. Natl. Acad. Sci. U. S. A.* 117 1139–1147. 10.1073/pnas.1912109117 31879343 PMC6969503

[B10] Delezie J. Dumont S. Dardente H. Oudart H. Grechez-Cassiau A. Klosen P. (2012). The nuclear receptor REV-ERBalpha is required for the daily balance of carbohydrate and lipid metabolism. *FASEB J.* 26 3321–3335. 10.1096/fj.12-208751 22562834

[B11] Dose J. Nebel A. Piegholdt S. Rimbach G. Huebbe P. (2016). Influence of the APOE genotype on hepatic stress response: Studies in APOE targeted replacement mice and human liver cells. *Free Radic. Biol. Med.* 96 264–272. 10.1016/j.freeradbiomed.2016.04.031 27130033

[B12] Dranka B. P. Hill B. G. Darley-Usmar V. M. (2010). Mitochondrial reserve capacity in endothelial cells: the impact of nitric oxide and reactive oxygen species. *Free Radic. Biol. Med.* 48 905–914. 10.1016/j.freeradbiomed.2010.01.015 20093177 PMC2860730

[B13] Duez H. Staels B. (2008). The nuclear receptors Rev-erbs and RORs integrate circadian rhythms and metabolism. *Diab. Vasc. Dis. Res.* 5 82–88. 10.3132/dvdr.2008.0014 18537094

[B14] Duez H. van der Veen J. N. Duhem C. Pourcet B. Touvier T. Fontaine C. (2008). Regulation of bile acid synthesis by the nuclear receptor Rev-erbalpha. *Gastroenterology* 135 689–698. 10.1053/j.gastro.2008.05.035 18565334

[B15] Fernandez C. G. Hamby M. E. McReynolds M. L. Ray W. J. (2019). The role of APOE4 in disrupting the homeostatic functions of astrocytes and microglia in aging and Alzheimer’s disease. *Front. Aging Neurosci.* 11:14. 10.3389/fnagi.2019.00014 30804776 PMC6378415

[B16] Flowers S. A. Rebeck G. W. (2020). APOE in the normal brain. *Neurobiol. Dis.* 136:104724. 10.1016/j.nbd.2019.104724 31911114 PMC7002287

[B17] Gibbs J. E. Blaikley J. Beesley S. Matthews L. Simpson K. D. Boyce S. H. (2012). The nuclear receptor REV-ERBalpha mediates circadian regulation of innate immunity through selective regulation of inflammatory cytokines. *Proc. Natl. Acad. Sci. U. S. A.* 109 582–587. 10.1073/pnas.1106750109 22184247 PMC3258648

[B18] Halliday M. R. Pomara N. Sagare A. P. Mack W. J. Frangione B. Zlokovic B. V. (2013). Relationship between cyclophilin a levels and matrix metalloproteinase 9 activity in cerebrospinal fluid of cognitively normal apolipoprotein e4 carriers and blood-brain barrier breakdown. *JAMA Neurol.* 70 1198–1200. 10.1001/jamaneurol.2013.3841 24030206 PMC4047029

[B19] Halliday M. R. Rege S. V. Ma Q. Zhao Z. Miller C. A. Winkler E. A. (2015). Accelerated pericyte degeneration and blood-brain barrier breakdown in apolipoprotein E4 carriers with Alzheimer’s disease. *J. Cereb. Blood Flow Metab.* 10.1038/jcbfm.2015.44 25757756 PMC4758554

[B20] Huang Y. (2006). Molecular and cellular mechanisms of apolipoprotein E4 neurotoxicity and potential therapeutic strategies. *Curr. Opin. Drug Discov. Devel.* 9 627–641. 17002223

[B21] Johnson L. A. (2020). APOE and metabolic dysfunction in Alzheimer’s disease. *Int. Rev. Neurobiol.* 154 131–151. 10.1016/bs.irn.2020.02.002 32739002

[B22] Kacimi R. Giffard R. G. Yenari M. A. (2011). Endotoxin-activated microglia injure brain derived endothelial cells via NF-kappaB, JAK-STAT and JNK stress kinase pathways. *J. Inflamm. (Lond.)* 8:7. 10.1186/1476-9255-8-7 21385378 PMC3061894

[B23] Koster K. P. Smith C. Valencia-Olvera A. C. Thatcher G. R. Tai L. M. LaDu M. J. (2017). Rexinoids as therapeutics for Alzheimer’s disease: role of APOE. *Curr. Top. Med. Chem.* 17 708–720. 10.2174/1568026616666160617090227 27320328

[B24] Lanfranco M. F. Ng C. A. Rebeck G. W. (2020). ApoE lipidation as a therapeutic target in Alzheimer’s disease. *Int. J. Mol. Sci.* 21:6336. 10.3390/ijms21176336 32882843 PMC7503657

[B25] Le Martelot G. Claudel T. Gatfield D. Schaad O. Kornmann B. Lo Sasso G. (2009). REV-ERBalpha participates in circadian SREBP signaling and bile acid homeostasis. *PLoS Biol.* 7:e1000181. 10.1371/journal.pbio.1000181 19721697 PMC2726950

[B26] Lewandowski C. T. Maldonado Weng J. LaDu M. J. (2020). Alzheimer’s disease pathology in APOE transgenic mouse models: the who, what, when, where, why, and how. *Neurobiol. Dis.* 139:104811. 10.1016/j.nbd.2020.104811 32087290 PMC7150653

[B27] Li J. Ye L. Wang X. Liu J. Wang Y. Zhou Y. (2012). (-)-Epigallocatechin gallate inhibits endotoxin-induced expression of inflammatory cytokines in human cerebral microvascular endothelial cells. *J. Neuroinflammation* 9:161. 10.1186/1742-2094-9-161 22768975 PMC3408337

[B28] Li Z. Shue F. Zhao N. Shinohara M. Bu G. (2020). APOE2: protective mechanism and therapeutic implications for Alzheimer’s disease. *Mol. Neurodegener.* 15:63. 10.1186/s13024-020-00413-4 33148290 PMC7640652

[B29] Liu C. C. Liu C. C. Kanekiyo T. Xu H. Bu G. (2013). Apolipoprotein E and Alzheimer disease: risk, mechanisms and therapy. *Nat. Rev. Neurol.* 9 106–118. 10.1038/nrneurol.2012.263 23296339 PMC3726719

[B30] Lutz S. E. Smith J. R. Kim D. H. Olson C. V. L. Ellefsen K. Bates J. M. (2017). Caveolin1 is required for Th1 cell infiltration, but not tight junction remodeling, at the blood-brain barrier in autoimmune neuroinflammation. *Cell Rep.* 21 2104–2117. 10.1016/j.celrep.2017.10.094 29166603 PMC5728697

[B31] Mahley R. W. Huang Y. Weisgraber K. H. (2007). Detrimental effects of apolipoprotein E4: potential therapeutic targets in Alzheimer’s disease. *Curr. Alzheimer Res.* 4 537–540. 10.2174/156720507783018334 18220516

[B32] Marottoli F. M. Katsumata Y. Koster K. P. Thomas R. Fardo D. W. Tai L. M. (2017). Peripheral inflammation, apolipoprotein E4, and amyloid-beta interact to induce cognitive and cerebrovascular dysfunction. *ASN Neuro.* 9:1759091417719201. 10.1177/1759091417719201 28707482 PMC5521356

[B33] Mayeuf-Louchart A. Thorel Q. Delhaye S. Beauchamp J. Duhem C. Danckaert A. (2017). Rev-erb-alpha regulates atrophy-related genes to control skeletal muscle mass. *Sci. Rep.* 7:14383. 10.1038/s41598-017-14596-2 29085009 PMC5662766

[B34] Moutinho M. Codocedo J. F. Puntambekar S. S. Landreth G. E. (2019). Nuclear receptors as therapeutic targets for neurodegenerative diseases: lost in translation. *Annu. Rev. Pharmacol. Toxicol.* 59 237–261. 10.1146/annurev-pharmtox-010818-021807 30208281 PMC6636329

[B35] Nagyoszi P. Wilhelm I. Farkas A. E. Fazakas C. Dung N. T. Hasko J. (2010). Expression and regulation of toll-like receptors in cerebral endothelial cells. *Neurochem. Int.* 57 556–564. 10.1016/j.neuint.2010.07.002 20637248

[B36] Najm R. Jones E. A. Huang Y. (2019). Apolipoprotein E4, inhibitory network dysfunction, and Alzheimer’s disease. *Mol. Neurodegener.* 14:24. 10.1186/s13024-019-0324-6 31186040 PMC6558779

[B37] Nishitsuji K. Hosono T. Nakamura T. Bu G. Michikawa M. (2011). Apolipoprotein E regulates the integrity of tight junctions in an isoform-dependent manner in an in vitro blood-brain barrier model. *J. Biol. Chem.* 286 17536–17542. 10.1074/jbc.M111.225532 21471207 PMC3093828

[B38] Pariollaud M. Gibbs J. E. Hopwood T. W. Brown S. Begley N. Vonslow R. (2018). Circadian clock component REV-ERBalpha controls homeostatic regulation of pulmonary inflammation. *J. Clin. Invest.* 128 2281–2296. 10.1172/JCI93910 29533925 PMC5983347

[B39] Pi X. Xie L. Patterson C. (2018). Emerging roles of vascular endothelium in metabolic homeostasis. *Circ. Res.* 123 477–494. 10.1161/CIRCRESAHA.118.313237 30355249 PMC6205216

[B40] Poels M. M. Vernooij M. W. Ikram M. A. Hofman A. Krestin G. P. van der Lugt A. (2010). Prevalence and risk factors of cerebral microbleeds: an update of the Rotterdam scan study. *Stroke* 41(10 Suppl) S103–S106. 10.1161/STROKEAHA.110.595181 20876479

[B41] Qin L. H. Huang W. Mo X. A. Chen Y. L. Wu X. H. (2015). LPS induces occludin dysregulation in cerebral microvascular endothelial cells via MAPK signaling and augmenting MMP-2 levels. *Oxid. Med. Cell Longev.* 2015:120641. 10.1155/2015/120641 26290681 PMC4531183

[B42] Raspe E. Duez H. Mansen A. Fontaine C. Fievet C. Fruchart J. C. (2002). Identification of Rev-erbalpha as a physiological repressor of apoC-III gene transcription. *J. Lipid Res.* 43 2172–2179. 10.1194/jlr.m200386-jlr200 12454280

[B43] Reitz C. J. Alibhai F. J. Khatua T. N. Rasouli M. Bridle B. W. Burris T. P. (2019). SR9009 administered for one day after myocardial ischemia-reperfusion prevents heart failure in mice by targeting the cardiac inflammasome. *Commun. Biol.* 2:353. 10.1038/s42003-019-0595-z 31602405 PMC6776554

[B44] Rieker C. Migliavacca E. Vaucher A. Baud G. Marquis J. Charpagne A. (2019). Apolipoprotein E4 expression causes gain of toxic function in isogenic human induced pluripotent stem cell-derived endothelial cells. *Arterioscler. Thromb. Vasc. Biol.* 39 e195–e207. 10.1161/ATVBAHA.118.312261 31315437

[B45] Salloway S. Gur T. Berzin T. Tavares R. Zipser B. Correia S. (2002). Effect of APOE genotype on microvascular basement membrane in Alzheimer’s disease. *J. Neurol. Sci.* 20 183–187. 10.1016/s0022-510x(02)00288-5 12417381

[B46] Sena C. M. Leandro A. Azul L. Seica R. Perry G. (2018). Vascular oxidative stress: impact and therapeutic approaches. *Front. Physiol.* 9:1668. 10.3389/fphys.2018.01668 30564132 PMC6288353

[B47] Serizawa F. Patterson E. Potter R. F. Fraser D. D. Cepinskas G. (2015). Pretreatment of human cerebrovascular endothelial cells with CO-releasing molecule-3 interferes with JNK/AP-1 signaling and suppresses LPS-induced proadhesive phenotype. *Microcirculation* 22 28–36. 10.1111/micc.12161 25098198

[B48] Solt L. A. Wang Y. Banerjee S. Hughes T. Kojetin D. J. Lundasen T. (2012). Regulation of circadian behaviour and metabolism by synthetic REV-ERB agonists. *Nature* 485 62–68. 10.1038/nature11030 22460951 PMC3343186

[B49] Stolwijk J. A. Matrougui K. Renken C. W. Trebak M. (2015). Impedance analysis of GPCR-mediated changes in endothelial barrier function: overview and fundamental considerations for stable and reproducible measurements. *Pflugers Arch.* 467 2193–2218. 10.1007/s00424-014-1674-0 25537398 PMC4480219

[B50] Tai L. M. Balu D. Avila-Munoz E. Abdullah L. Thomas R. Collins N. (2017). EFAD transgenic mice as a human APOE relevant preclinical model of Alzheimer’s disease. *J. Lipid Res.* 58 1733–1755. 10.1194/jlr.R076315 28389477 PMC5580905

[B51] Tai L. M. Ghura S. Koster K. P. Liakaite V. Maienschein-Cline M. Kanabar P. (2015). APOE-modulated Abeta-induced neuroinflammation in Alzheimer’s disease: current landscape, novel data, and future perspective. *J. Neurochem.* 133 465–488. 10.1111/jnc.13072 25689586 PMC4400246

[B52] Tai L. M. Thomas R. Marottoli F. M. Koster K. P. Kanekiyo T. Morris A. W. (2016). The role of APOE in cerebrovascular dysfunction. *Acta Neuropathol.* 131 709–723. 10.1007/s00401-016-1547-z 26884068 PMC4837016

[B53] Tang X. Luo Y. X. Chen H. Z. Liu D. P. (2014). Mitochondria, endothelial cell function, and vascular diseases. *Front. Physiol.* 5:175. 10.3389/fphys.2014.00175 24834056 PMC4018556

[B54] Thomas R. Morris A. W. J. Tai L. M. (2017). Epidermal growth factor prevents APOE4-induced cognitive and cerebrovascular deficits in female mice. *Heliyon* 3:e00319. 10.1016/j.heliyon.2017.e00319 28626809 PMC5463012

[B55] Thomas R. Zuchowska P. Morris A. W. Marottoli F. M. Sunny S. Deaton R. (2016). Epidermal growth factor prevents APOE4 and amyloid-beta-induced cognitive and cerebrovascular deficits in female mice. *Acta Neuropathol. Commun.* 4:111. 10.1186/s40478-016-0387-3 27788676 PMC5084423

[B56] Urbano R. L. Swaminathan S. Clyne A. M. (2019). Stiff substrates enhance endothelial oxidative stress in response to protein kinase C activation. *Appl. Bionics Biomech.* 2019:6578492. 10.1155/2019/6578492 31110559 PMC6487160

[B57] Wang S. Li F. Lin Y. Wu B. (2020). Targeting REV-ERBalpha for therapeutic purposes: promises and challenges. *Theranostics* 10 4168–4182. 10.7150/thno.43834 32226546 PMC7086371

[B58] Wang S. Lin Y. Yuan X. Li F. Guo L. Wu B. (2018). REV-ERBalpha integrates colon clock with experimental colitis through regulation of NF-kappaB/NLRP3 axis. *Nat. Commun.* 9:4246. 10.1038/s41467-018-06568-5 30315268 PMC6185905

[B59] Werb Z. Chin J. R. Takemura R. Oropeza R. L. Bainton D. F. Stenberg P. (1986). The cell and molecular biology of apolipoprotein E synthesis by macrophages. *Ciba Found. Symp.* 118 155–171. 10.1002/9780470720998.ch11 3525037

[B60] Woldt E. Sebti Y. Solt L. A. Duhem C. Lancel S. Eeckhoute J. (2013). Rev-erb-alpha modulates skeletal muscle oxidative capacity by regulating mitochondrial biogenesis and autophagy. *Nat. Med.* 19 1039–1046. 10.1038/nm.3213 23852339 PMC3737409

[B61] Yamazaki Y. Liu C. C. Yamazaki A. Shue F. Martens Y. A. Chen Y. (2020a). Vascular ApoE4 impairs behavior by modulating gliovascular function. *Neuron* 109 438–447.e6. 10.1016/j.neuron.2020.11.019 33321072 PMC7864888

[B62] Yamazaki Y. Shinohara M. Yamazaki A. Ren Y. Asmann Y. W. Kanekiyo T. (2020b). ApoE (Apolipoprotein E) in brain pericytes regulates endothelial function in an isoform-dependent manner by modulating basement membrane components. *Arterioscler. Thromb. Vasc. Biol.* 40 128–144. 10.1161/ATVBAHA.119.313169 31665905 PMC7007705

[B63] Yamazaki Y. Zhao N. Caulfield T. R. Liu C. C. Bu G. (2019). Apolipoprotein E and Alzheimer disease: pathobiology and targeting strategies. *Nat. Rev. Neurol.* 15 501–518. 10.1038/s41582-019-0228-7 31367008 PMC7055192

[B64] Yin L. Wu N. Lazar M. A. (2010). Nuclear receptor Rev-erbalpha: a heme receptor that coordinates circadian rhythm and metabolism. *Nucl. Recept. Signal.* 8:e001. 10.1621/nrs.08001 20414452 PMC2858265

[B65] Zhao Z. Hu J. Gao X. Liang H. Liu Z. (2014). Activation of AMPK attenuates lipopolysaccharide-impaired integrity and function of blood-brain barrier in human brain microvascular endothelial cells. *Exp. Mol. Pathol.* 97 386–392. 10.1016/j.yexmp.2014.09.006 25220346

[B66] Zipser B. D. Johanson C. E. Gonzalez L. Berzin T. M. Tavares R. Hulette C. M. (2007). Microvascular injury and blood-brain barrier leakage in Alzheimer’s disease. *Neurobiol. Aging* 28 977–986. 10.1016/j.neurobiolaging.2006.05.016 16782234

[B67] Zlokovic B. V. (2013). Cerebrovascular effects of apolipoprotein E: implications for Alzheimer disease. *JAMA Neurol.* 70 440–444. 10.1001/jamaneurol.2013.2152 23400708 PMC4414030

