# Supplementary material for: Autocrine Effects of Brain Endothelial Cell-Produced Human Apolipoprotein E on Metabolism and Inflammation in vitro
Source: Front Cell Dev Biol. 2021 Jun 10;9:668296. doi: 10.3389/fcell.2021.668296 (PMC8225247; doi:10.3389/fcell.2021.668296)
Supplement: Supplementary file 1 [file Data_Sheet_1.PDF]

Supplementary Material

Table of Contents

1 SUPPLEMENTARY MATERIALS AND METHODS 2

1.1 DETAILED BEC ISOLATION PROTOCOL 2
1.2 *IN SITU* HYBRIDIZATION, IMMUNOCYTOCHEMISTRY AND WESTERN BLOT ANALYSIS 10
1.2.1 In situ hybridization 10
1.2.2 Immunocytochemistry 10
1.2.3 Western blot analysis. 10
1.3 RNA SEQUENCING 11
1.4 METABOLOMICS 12
1.5 ATOMIC FORCE MICROSCOPY 14
1.5.1 Bio-functionalization of the AFM cantilevers 14
1.5.2 Measurements of cell stiffness, membrane tethering force and adhesion probability 14
1.6 LEUKOCYTE ADHESION ASSAY 14
SUPPLEMENTARY TABLE 1: DETAILED MATERIALS AND METHODS 15

2 SUPPLEMENTARY RESULTS 27

SUPPLEMENTARY TABLE 2: FUNCTIONAL CLUSTERS OF DIFFERENTIALLY EXPRESSED GENES IN *APOE4* VERSUS *APOE3* BRAIN ENDOTHELIAL CELLS 27
FIGURE S1. BRAIN ENDOTHELIAL CELL-PRODUCED APOE3 AND APOE4 DO NOT MIGRATE DIFFERENTLY IN NATIVE GEL ELECTROPHORESIS. 31
FIGURE S2. HIGHER LEVELS OF MITOCHONDRIAL COMPLEX I MARKER NDUFB IN *APOE4* BRAIN ENDOTHELIAL CELLS. 32
FIGURE S3. TRIGLYCERIDE, BUT NOT CHOLESTEROL, LEVELS ARE LOWER IN MEDIA COLLECTED FROM *APOE4* BRAIN ENDOTHELIAL CELLS. 33
FIGURE S4. APOE LEVELS ARE MODULATED BY LPS. 34
FIGURE S5. FULL WESTERN BLOT IMAGES AND QUANTIFICATION 35

3 SUPPLEMENTARY REFERENCES 36

1 Supplementary Materials and Methods

1.1 Detailed BEC Isolation Protocol

Isolation of Primary BECs from P21-P28 Mice

This protocol was based on modifying previously described methods of brain endothelial cell isolation (Ichikawa et al., 1996; Welser-Alves et al., 2014; Wang et al., 2015).

Materials & Reagents:

Material/Reagent	Company	Catalog #	Storage
Fibronectin	Sigma	F0895	4°C
Collagen from calf skin	Sigma	C8919	4°C
Laminin	Sigma	L2020-1MG	-20°C
Poly-D-lysine hydrobromide	Sigma	P7280	-20°C
HBSS, calcium, magnesium, no phenol red (+/+)	Gibco	14-025-092	RT
Minimum Essential Media Eagle (MEM)	Sigma-Aldrich	M7278	4°C
Penicillin-Streptomycin Solution, 100X	Corning	30-002-CI	-20°C
Papain	Worthington Biochemical	LK003178	4°C
DNase	Worthington Biochemical	LK003172	4°C
Heparin sodium salt from porcine intestinal mucosa	Sigma-Aldrich	H3149	4°C
HBSS, no calcium, no magnesium, no phenol red (-/-)	Gibco	14175-095	RT
HEPES (1 M)	Gibco	15630-080	4 °C
Bovine Serum Albumin (BSA), Fraction V	Gemini	700-100P	4°C & -20°C
EGM-2 MV BulletKit (CC-3156 & CC-4147)	Lonza	CC-3202	4°C & -20°C
Ham's F-12 Nutrient Mix	Gibco	11765-054	4°C
Puromycin	Sigma	P8833	-20°C
100 x 15 mm Tissue Culture Dishes	Corning	351029	RT
60 x 15 mm Tissue Culture Dishes	Corning	353004	RT
19G Precision Glide Needle	BD	305187	RT
21G Precision Glide Needle	BD	305167	RT
1 ml Syringe	BD	309659	RT
5 ml Syringe	BD	309646	RT
10 ml Syringe	BD	302995	RT
20 ml Syringe	BD	309646	RT
pluriStrainer Mini 100 µm Cell Strainer	pluriSelect-USA Inc	43-10100-60	RT
Surgical Scissors - Tungsten Carbide	Fine Science Tools	14502-14	RT
Littauer Bone Cutters	Fine Science Tools	16152-11	RT
Graefe Forceps (2 Pairs)	Fine Science Tools	11051-10	RT
Razor Blades			

Solution Preparation:

10 mg/ml Poly-D-Lysine: *For glass growth surfaces only*. Dilute 1 mg/ml poly-D-lysine stock 1:100 in sterile tissue culture grade water. (Stock solution is also prepared in tissue culture grade water.)

50 µg/ml Fibronectin: Dilute 0.5 ml fibronectin per 9.5 ml of HBSS (+/+) for a final concentration of 50 µg/ml. Make 10 ml at a time and reuse until it runs out.

0.005% Collagen + 1-2 µg/ml Laminin: Dilute 0.5 ml 0.1% collagen per 9.5 ml of HBSS (+/+). Add 12.5 µl of 1-2 mg/ml laminin per 10 ml of 0.005% collagen. Volume can be scaled up to accommodate need.

MEMα: Add 5 ml of 100X Pen/Strep to a 500 ml bottle of MEM.

Papain Solution: Reconstitute one vial of papain in 5 ml of MEMα. Each vial is enough for approximately 6 brains.

DNase Solution: Reconstitute one vial of DNase in 0.5 ml of MEMα. Each vial is enough for approximately 12 mouse brains.

Digestion Buffer: Mix papain solution and DNase solution at a volume ratio of 20:1 papain:DNase (i.e. 2 vials of prepared papain solution:1 vial of prepared DNase solution) in a conical tube. Scale volumes accordingly for the number of brains and/or smaller brain regions. 5 ml of Digestion buffer is sufficient for 6 cortices.

Note: Unused digestion buffer can be stored at 4°C for up to 1 week.

25% BSA: Prepare BSA under sterile conditions (even though powder is not sterile). In the tissue culture hood, add 100g of BSA to approximately 400 ml of HBSS (-/-) containing 1X Pen/Strep in a 2 L bottle with a stir bar. Cap bottle and set on stir plate to dissolve. Once BSA is dissolved, return to tissue culture hood and transfer solution to a graduated cylinder to bring the final volume to 500 ml. Aliquot and store at -20°C. 25% BSA can be freeze/thawed once if a substantial amount remains after first use and sterility is maintained.

10,000 U/ml Heparin: Re-suspend 10,000 units of heparin sodium salt per 1 ml of tissue-culture grade water and pass through a 0.22 µm syringe filter under sterile conditions.

Complete ECM: Thaw supplement kit (CC-4147) and add a full vial of each supplement to 500 ml of endothelial cell medium (ECM; CC-3156). Store at 4°C.

Basal ECM: *For experiments where serum-free and growth factor-free conditions are required.* Thaw supplement kit (CC-4147) and add a full vial of each supplement ***EXCEPT growth factors and FBS*** to 500 ml of endothelial cell medium (CC-3156). Store at 4°C.

Complete ECM+Heparin: Add 27.5 µl of 10,000 U/ml to 50 ml of Complete ECM. Excess can be stored at 4°C for future use. If you are isolating in high volume, you can prepare an entire bottle of Complete ECM+Heparin by adding 275 µl of 10,000 U/ml to 500 ml of Complete ECM and store at 4°C.

Setup:

<u>In Advance:</u>

1. Dilute 100X Pen/Strep to 1X in all HBSS and F12 solutions for use with primary cell culture.
2. Prepare 25% BSA.
3. Prepare 10,000U/ml heparin.
4. Prepare Complete ECM if needed.
5. Prepare 8 mg/ml (1000x) puromycin stock.
6. Label 3 sets of conical tubes per isolation with the isolation number; label two 50 ml and one 15 ml tube for >3 brains (i.e., T25 flask and larger), otherwise label only 15 ml tubes. The third tube will always be a 15 ml tube.

Note: It is important to use tubes that are made from a low binding material with no ridges on the interior as this will compromise the yield and ultimately the quality of the isolation.

7. In the tissue culture hood, label one small Petri dish per isolation with the isolation number, genotype, sex, and number of brains. Dishes can remain in hood until isolation if labeled in advance.
8. Coat surfaces with poly-D-lysine <u>if isolating onto a glass growth surface</u>:
    a. Add 10 µg/ml poly-D-lysine for 30-60 minutes at 37°C.
    b. Wash 5x with sterile Milli-Q water.

    Note: We recommend coating in poly-D-lysine the night before and leaving the slides at 4°C in water until the isolation.

<u>Day of</u>:

Cells are seeded at a ratio of approximately 1 cortex per 3.466 cm^2 (see **Table 1**).

1. Coat growth surfaces with 50 µg/ml fibronectin:
    a. Add fibronectin to growth surface, ensuring that it covers entire bottom.
    b. Immediately remove fibronectin (save to reuse) and allow the surface to dry inside the tissue culture hood (no cap/lid).

    IMPORTANT: For isolation onto 96w10idf plates for TEER, do not let the fibronectin fully dry as it may damage the gold electrode. Instead, remove fibronectin and move on to collagen/laminin coating within 5 min.

    c. Repeat steps 1a and 1b if isolating onto glass surfaces.

2. Coat growing surfaces with 0.005% collagen + 1-2 µg/ml laminin mixture at 37°C for at least one hour. Wash 2x with HBSS (+/+) containing 1X Pen/Strep.

3. For TEER experiments:
   a. Remove HBSS (+/+) washes and flood wells with 10 mM L-cysteine for at least 15 min to 1 hr at RT.
   b. Wash 1x with HBSS (+/+) and replace with Complete ECM+Heparin until it is time for plating.

Table 1: Cell Plating and Media Volumes

Type	Manufacturer	Catalog Number	Growth Area Per Well (cm^2)	Brains Per Well	Wells Per Brain	Plating Volume Per Well (ml)	Maintenance Volume Per Well (ml)
T75 Flask	TPP	90076	75	21		15	37.5
T25 Flask	TPP	90026	25	7		5	12.5
6 Well	TPP	92006	9.026	4		2	5
12 Well	TPP	92012	3.466	1	1	1	2
24 Well	TPP	92024	1.864		2	0.5	1
96 Well	Greiner Bio-One	655090	0.34		6	0.1	0.2
96w10idf	Applied Biophysics	96w10idf	0.34		6	0.1	0.2
12 Well Chamber Slide	ibidi	81201	0.56		4	0.15	0.3
Ibidi Culture Insert 4-Well µ-Dish	Ibidi	80466	0.35		6	0.1	0.15
Seahorse XF96 FluxPak	Agilent	102416-100	0.1099		6	0.08	0.08

4. Prepare Papain Solution, DNase Solution, Digestion Buffer and ECM+Heparin as needed.
5. Place 25% BSA and ECM+Heparin in 37 °C water bath.

   IMPORTANT: For isolation onto 96w10idf plates for TEER, once media is warmed, remove it from the water bath and allow it to come to RT for plating.

6. Fill the prelabeled 60 mm Petri dishes with cold 5 ml of MEMα for each isolation and place on ice inside the hood.
7. Fill assembled syringes (19G needle) with ~700 µl of Digest Buffer per cortex the morning of the isolation and leave at RT (see **Table 2**).

Table 2: Digestion Setup

Number of Brains	Syringe Size	Digestion Volume (ml)
1	1 ml	0.8*
3	5 ml	2.4
7	10 ml	5.6
21	20 ml	16.8

* Fill 1 ml syringe to 0.75 as the void volume is about .05 ml.

8. Remove tools from autoclave pouch, submerge them in 70% ethanol and place in tissue culture hood.
9. Liberally spray paper towels with 70% ethanol and allow them to dry inside the tissue culture hood.
10. When ready to begin dissecting, drench a flat ice pack with 70% ethanol and place in the hood. Cover with a paper towel.

<u>On day after endothelial cell isolation</u>:

1. Prepare Complete ECM containing 8 µg/ml puromycin from stock solution and warm to 37°C.

Isolation Procedure:

1. Place mice in CO_2 chamber and administer CO_2 at a displacement rate of 30% air volume per minute according to the meter attached to the regulator. Maintain the gas flow for at least 1 minute after the animals succumb to respiratory arrest.
2. Decapitate mice outside of the hood and place heads in a petri dish.
3. Spray heads liberally with 70% ethanol and move them into the tissue culture hood.

Note: Perform the dissections on a sterile paper towel atop a flat ice pack. Replace the paper towel between isolations of separate sex and genotype, or as needed.

4. For each mouse, liberate the brain from the skull, roll the brain around on ethanol-sterilized paper towel to remove meninges, and dissect out the cortex (discard the hippocampus, striatum, midbrain, and cerebellum unless isolating cells from these regions) and place in prelabeled Petri dish(es) containing cold MEMα. Maintain Petri dishes on ice until you are ready to mince the tissue.
5. Mince cortices in the MEMα using a sterile razor blade (one razor blade per Petri dish).
6. Move the tissue to one side of the dish and transfer the brain fragments and accompanying MEMα to a 15 ml conical tube using a trimmed P1000 pipet tip.

Note: For large isolation numbers, move to 4°C or keep on ice until all are complete, if necessary.

7. Centrifuge at 1000 × *g* for 5 min at 4°C.
8. Carefully discard supernatant and use a 19G needle to add the appropriate amount of Digestion Buffer (700 µl per cortex; see **Solution Preparation**) to the pellet. Pass the tissue through the needle once; twice if needed to completely pass all the tissue through the syringe.

IMPORTANT: Leave a gap between homogenate and plunger so as not to lose any tissue to void.

9. Digest tissue for 15 min at 37°C.

IMPORTANT: Long digestion times will decrease yield and quality of cultures. It is best to start a timer and when the first sample is resuspended in Digestion Buffer to standardize the amount of time each isolation is exposed to enzymatic digestion (i.e., start the timer once the first sample is processed, proceed with remaining isolations, move the tubes to the water bath, then remove all the tubes and proceed to the next step once the timer goes off). Samples should be at 37°C by the time 2.5 min has elapsed.

10. Pass digested tissue through a 21G needle as before (Step 8).

IMPORTANT: Leave a gap between homogenate and plunger so as not to lose any tissue to void.

11. Add 2 ml of 25% BSA per cortex and vortex briefly.

IMPORTANT: Since BSA will settle following initial preparation is critical that the 25% BSA be thoroughly mixed prior to being added to the digested tissue. Once the 25% BSA has thawed, allow it to mix on a shaker until needed. Once in the hood, mix the 25% BSA by inverting the tube several times to ensure there are no visible swirls from areas of concentrated BSA.

12. Centrifuge at 4000 × *g* for 5 min at 4°C.
13. Carefully rotate the tube at an angle to dislodge the myelin plug, then swiftly invert and pour the supernatant and plug into a fresh tube and set aside until Step 15. Leave the tube containing the pellet inverted on a clean paper towel until ready to proceed to the next step.

Note: It is important to do this quickly, as prolonged exposure to BSA can be toxic.

14. Re-suspend the pellet in a total volume of 1 ml of Complete ECM+Heparin and pass through 100 µm cell strainer into the final tube.

Note: Set aside the tube with the cell strainer until Step 17 as the pellet from the second BSA spin will be added to this.

15. Vortex the BSA mixture again and centrifuge at 4000 × *g* for 5 min at 4°C.
16. Carefully dislodge the myelin plug as in Step 13 and discard the BSA supernatant. Leave the tube containing the pellet inverted on a clean paper towel until ready to proceed to the next step.
17. Re-suspend the pellet from the second centrifugation in a total volume of 1 ml of Complete ECM+Heparin and pass through the 100 µm cell strainer to combine it with the already resuspended pellet from the first BSA spin (Step 14).
18. Flush cell strainer with 2 ml of ECM+Heparin.
19. Centrifuge at 1000 × *g* for 5 min at 4°C.
20. Discard the supernatant and invert the tube on a clean paper towel.
21. Resuspend the pellet in the appropriate volume of ECM+Heparin (See ***Table 1***), plate cells and transfer to 37°C.

IMPORTANT: When plating in 6 and 12 well plates, it is important to evenly distribute the cells over the growth area as they tend to pool in the center. Leave the cells at RT for 15 min. Gently shake the plate ever 2-3 minutes left to right and back and forth being careful to **avoid a swirling motion**.

IMPORTANT: For isolation onto 96w10idf plates for TEER, allow plated cells to remain at RT for 20-30 min before transferring to 37°C.

22. CRITICAL STEP. 4-6 h after plating, gently wash cells with warm F12 with just enough agitation to remove red blood cells and large debris (See ***Table 3***). Replace F12 with a maintenance volume of fresh ECM+Heparin (See ***Table 1***).

IMPORTANT: Washing the cells is one of the most critical steps in the protocol and over washing will greatly impact the yield. It is better to slightly under wash the cells on the day of the isolation than to over wash; this step is to prevent debris and red blood cells from burying the endothelial cells.

Table 3: Procedure for Washing Away Debris from Isolation.

Type	Manufacturer	Catalog Number	Washing Procedure
T75 Flask	TPP	90076	Using a serological pipet for flasks or a P1000 for plates, gently replace media with F12. Allow media to flow over the growth surface and carry away the debris until the red haze of red blood cells is no longer visible. Check under a microscope to make sure there is no large debris. If there is, wash once or twice more. Stubborn debris can be cleared the following morning during the second wash.
T25 Flask	TPP	90026	
6 Well	TPP	92006	
12 Well	TPP	92012	
24 Well	TPP	92024	
96 Well	Greiner Bio-One	655090	Using a multichannel or P200 pipet, bring the well volume to 0.3 ml by adding F12 **very** slowly to the center of the well. Very gently pipet up and down 4-5 times around the edges of the well to dislodge any debris. It is important to do this slowly as too much force will dislodge the endothelial cells too.
96w10idf	Applied Biophysics	96w10idf	
12 Well Chamber Slide	ibidi	81201	
Ibidi Culture Insert 4-Well µ-Dish	ibidi	80466	
Seahorse XF96 FluxPak	Agilent	102416-100	

23. The next morning when brain endothelial cells are more firmly adhered, wash growth surfaces a second time with slightly more turbulence than the night before, still being careful not to over wash. Check under microscope to confirm that brain endothelial cells are still firmly attached (you should not lose any attached cells from this wash – only debris).

Note: Debris on top of brain endothelial cells will look as if none of the cells survived. Once you have washed a second time, you will see brain endothelial cells that have attached and spread as single cells and small colonies.

24. Once cells are thoroughly washed, add the appropriate volume of Complete ECM containing 8 µg/ml puromycin (See ***Table 1***) for 48 h to negatively select for endothelial cells.
25. After 48 h, remove media containing puromycin and wash the cells with HBSS (+/+) using more force than the previous 2 washes. Check under the microscope to gauge that you are using enough force to clear the debris without disturbing the endothelial cells beneath. Replace with a maintenance volume of Complete ECM (See ***Table 1***).
26. Change media every other day until cells completely cover growing surface. Cells should be confluent and ready for experiments by 7 days *in vitro*.

1.2 *In situ* hybridization, immunocytochemistry and western blot analysis

1.2.1 *In situ* hybridization

APOE mRNA was detected *in situ* with BaseScope™ Detection Reagent Kit v2 – RED (ACD, 323900). Succinctly, cells were rinsed twice with PBS and fixed in 10% Neutral Buffered Formalin for 30 min at 4°C. Cell were washed once more with PBS and then pretreated with RNAscope® hydrogen peroxide for 10 min at room temperature followed by 3 washes with PBS. Cells were then treated with a 1:15 dilution of RNAscope® Protease III solution in PBS for 10 min at room temperature then washed twice in PBS. Probe hybridization and amplification was performed as directed by the manufacturer protocol (323900-USM) with the exception that washes with distilled water were substituted with PBS washes. Following the signal detection steps, cells were probed for ZO-1 by immunocytochemistry and counterstained with DAPI (see below).

1.2.2 Immunocytochemistry

Cells were rinsed in PBS and then fixed in either 70% ethanol at room temperature for 5 min or in 3% PFA in PBS overnight at 4°C, washed twice with PBS, and permeabilized with 0.1% Tween-20 in PBS (0.1% PBS-T) for 5 min at room temperature. Cells fixed in 3% PFA were then quenched in 0.05M NH_4Cl for 10 min at room temperature. Samples were blocked in 10% FBS in 0.05% PBS-T for 30 min at room temperature then washed once with 0.05% PBS-T. Primary antibodies were diluted 1:100 in 3% FBS in 0.05% PBS-T and incubated overnight at 4°C. Following incubation in primary antibody, cells were washed 3 times in 0.05% PBS-T (list of antibodies and dilutions provided in **Supplementary Table 1**). Secondary antibodies were diluted 1:200 in 3% FBS in 0.05% PBS-T and incubated for 1 h at room temperature. Cells were washed 3 times in 0.05% PBS-T, then once in PBS, and followed by DAPI (Thermo, 62248) diluted 1:1000 in PBS for 10 min at room temperature. Cells were washed twice more in PBS and coverslipped using Ecomount mounting medium.

1.2.3 Western blot analysis.

Cell-associated protein levels were measured in brain endothelial cell lysates by western blot total analysis as described in (Marottoli et al., 2017). Brain endothelial cells were lysed in RIPA buffer, sonicated on ice (20% amplification for 2 cycles of 15 s) and centrifuged at 14,000 × *g* for 15 min at 4°C. Total protein in SDS extracts was quantified using the Pierce™ BCA Protein Assay Kit (Thermo Fisher, 23225). 15 µg of protein were separated under denaturing conditions on 4-12% Bis-Tris gels (Invitrogen, NP0322BOX) and transferred in Towbin buffer with 10% methanol to PVDF membrane at 0.45A for 2.5 h at 4°C. For native gels, 17.5 ml of culture medium conditioned for 48h by confluent brain endothelial cells was separated on 4-20% Tris-Glycine gels (Invitrogen, XP04205BOX) according to the manufacturer protocol (MAN0014610) and transferred to PVDF as described for cell lysates but with the omission of methanol. Membranes were washed once in TBS and blocked for 1 h at room temperature in 5% non-fat milk (w/v) in TBS. Membranes were then rinsed 3 times in 0.1% Tween-20 in TBS (0.1% TBS-T) and incubated in primary antibodies in 1% BSA (w/v) with 0.02% sodium azide (w/v) in TBS, pH 7.4-7.6 (list of antibodies, incubation conditions and dilutions are provided in **Supplementary Table 1**). Following incubation with primary antibodies, membranes were washed 3 times in 0.1% TBS-T and incubated for 45 min in the appropriate secondary antibody in 1% non-fat milk (w/v) in 0.1% TBS-T with 0.01% SDS, pH 7.4-7.6. Membranes were then washed three times in 0.1% TBS-T followed by one wash in TBS and imaged using a Li-Cor Odessy® Fc Imaging System. Optical density was quantified with Image Studio Lite v5.2.

1.3 RNA Sequencing

Total RNA was extracted with the miRNeasy Mini Kit (Qiagen, 217004) and used for sequencing library construction. RNA samples were quantified using NanoDrop™ One Spectrophotometer (Thermo Scientific) and analyzed for integrity using 4200 TapeStation (Agilent). Levels of remaining DNA were checked using Qubit fluorometer (Invitrogen). DNA amounts did not exceed 10% of the total amount of NA.

Sequencing libraries for Illumina sequencing were prepared using 100 ng of total RNA per sample. Library prep was carried with the Universal Plus mRNA-Seq kit (NuGen, 0520-A01), as written in the product manual (NuGen, M01485 v5). In brief, RNA underwent poly-A selection, enzymatic fragmentation, and generation of double-stranded cDNA using a mixture of oligo (dT) and random priming. The cDNA underwent end repair, ligation of dual-index adaptors, strand selection, and 17 cycles of PCR amplification. The number of cycles was determined by qPCR on a small aliquot of the un-amplified libraries. All intermediate purification steps, and final library purification was carried out using Agencourt AMPure XP Beads (Beckman Coulter A63881).

Final amplified libraries were measured with the Qubit 1X dsDNA HS Assay Kit (Invitrogen, Q33231), and fragment size distribution was confirmed using the D5000 ScreenTape assay (Agilent, 5067-5588, 5067-5589). Concentration of the final library pool was confirmed by qPCR, and subjected to test sequencing in order to check sequencing efficiencies and adjust accordingly proportions of individual libraries. The pool was purified with the Agencourt AMPure XP Beads (Beckman Coulter A63881), quantified by qPCR using KAPA Library Quantification Kit and run on a NovaSeq6000 SP flow cell, 2x50 nt, two lanes of , at the University of Illinois Roy J. Carver Biotechnology Center High-Throughput Sequencing and Genotyping Unit.

Raw reads were aligned to custom reference consisting of mm10 genome and hg38 human APOE Gene (ENST00000485628.2, ENST00000425718.1, ENST00000434152.5, ENST00000252486.8, ENST00000446996.5) using STAR (Dobin et al., 2013) (Dobin et al., 2013). ENSEMBL genes were quantified using FeatureCounts (Liao et al., 2014). Differential expression statistics were computed using edgeR (McCarthy et al., 2012) on raw expression counts with the exactTest function. P-values were adjusted for multiple testing using the false discovery rate (FDR) correction of (Benjamini and Hochberg, 1995)

1.4 Metabolomics

Sample Collection: Media was removed, aliquoted and stored at -80°C. Cells were briefly rinsed in HBSS (-/-). Cells were then incubated in 1 ml HBSS (-/-) for 10 min at 37°C followed by a 10 min in 1 ml 0.25 % trypsin-EDTA (25200-072, Invitrogen) at 37°C. Trypsin was neutralized with 2 ml of complete media and cells were pelleted at 1000 × *g* for 3 min. All but 0.75 ml of the supernatant was discarded. Cells were resuspended in remaining supernatant and then pelleted a second time at 1000 × *g* for 3 min. Supernatant was carefully removed and samples were flash frozen and stored at -80°C until shipment.

Note: Samples were processed by Metabolon.

Sample Accessioning: Samples were inventoried and immediately stored at -80°C until processed. Each sample received was accessioned into the Metabolon Laboratory Information Management System (LIMS) system and was assigned by the LIMS a unique identifier that was associated with the original source identifier only. This identifier was used to track all sample handling, tasks, results, etc. The samples (and all derived aliquots) were tracked by the LIMS system. All portions of any sample were automatically assigned their own unique identifiers by the LIMS when a new task was created; the relationship of these samples was also tracked.

Sample Preparation: Samples were prepared using the automated MicroLab STAR® system from Hamilton Company. Several recovery standards were added prior to the first step in the extraction process for QC purposes. To remove protein, dissociate small molecules bound to protein or trapped in the precipitated protein matrix, and to recover chemically diverse metabolites, proteins were precipitated with methanol under vigorous shaking for 2 min (Glen Mills GenoGrinder 2000) followed by centrifugation. The resulting extract was divided into five fractions: two for analysis by two separate reverse phases (RP)/UPLC-MS/MS methods with positive ion mode electrospray ionization (ESI), one for analysis by RP/UPLC-MS/MS with negative ion mode ESI, one for analysis by HILIC/UPLC-MS/MS with negative ion mode ESI, and one sample was reserved for backup. Samples were placed briefly on a TurboVap® (Zymark) to remove the organic solvent. The sample extracts were stored overnight under nitrogen before preparation for analysis.

QA/QC: Several types of controls were analyzed in concert with the experimental samples: a pooled matrix sample generated by taking a small volume of each experimental sample (or alternatively, use of a pool of well-characterized human plasma) served as a technical replicate throughout the data set; extracted water samples served as process blanks; and a cocktail of QC standards that were carefully chosen not to interfere with the measurement of endogenous compounds were spiked into every analyzed sample, allowed instrument performance monitoring and aided chromatographic alignment. Instrument variability was determined by calculating the median relative standard deviation (RSD) for the standards that were added to each sample prior to injection into the mass spectrometers. Overall process variability was determined by calculating the median RSD for all endogenous metabolites (i.e., non-instrument standards) present in 100% of the pooled matrix samples. Experimental samples were randomized across the platform run with QC samples spaced evenly among the injections.

Ultrahigh Performance Liquid Chromatography-Tandem Mass Spectroscopy (UPLC-MS/MS): All methods utilized a Waters ACQUITY ultra-performance liquid chromatography (UPLC) and a Thermo Scientific Q-Exactive high resolution/accurate mass spectrometer interfaced with a heated electrospray ionization (HESI-II) source and Orbitrap mass analyzer operated at 35,000 mass resolution. The sample extract was dried then reconstituted in solvents compatible to each of the four methods. Each reconstitution solvent contained a series of standards at fixed concentrations to ensure injection and chromatographic consistency. One aliquot was analyzed using acidic positive ion conditions, chromatographically optimized for more hydrophilic compounds.

In this method, the extract was gradient eluted from a C18 column (Waters UPLC BEH C18-2.1x100 mm, 1.7 µm) using water and methanol, containing 0.05% perfluoropentanoic acid (PFPA) and 0.1% formic acid (FA). Another aliquot was also analyzed using acidic positive ion conditions; however, it was chromatographically optimized for more hydrophobic compounds. In this method, the extract was gradient eluted from the same afore mentioned C18 column using methanol, acetonitrile, water, 0.05% PFPA and 0.01% FA and was operated at an overall higher organic content. Another aliquot was analyzed using basic negative ion optimized conditions using a separate dedicated C18 column. The basic extracts were gradient eluted from the column using methanol and water, however with 6.5mM Ammonium Bicarbonate at pH 8. The fourth aliquot was analyzed via negative ionization following elution from a HILIC column (Waters UPLC BEH Amide 2.1x150 mm, 1.7 µm) using a gradient consisting of water and acetonitrile with 10mM Ammonium Formate, pH 10.8. The MS analysis alternated between MS and data-dependent MS^n scans using dynamic exclusion. The scan range varied slighted between methods but covered 70-1000 m/z. Raw data files are archived and extracted.

Data Extraction and Compound Identification: Raw data was extracted, peak-identified and QC processed using Metabolon's hardware and software. These systems are built on a web-service platform utilizing Microsoft's .NET technologies, which run on high-performance application servers and fiber-channel storage arrays in clusters to provide active failover and load-balancing. Compounds were identified by comparison to library entries of purified standards or recurrent unknown entities. Metabolon maintains a library based on authenticated standards that contains the retention time/index (RI), mass to charge ratio (*m/z)*, and chromatographic data (including MS/MS spectral data) on all molecules present in the library. Furthermore, biochemical identifications are based on three criteria: retention index within a narrow RI window of the proposed identification, accurate mass match to the library +/- 10 ppm, and the MS/MS forward and reverse scores between the experimental data and authentic standards. The MS/MS scores are based on a comparison of the ions present in the experimental spectrum to the ions present in the library spectrum. While there may be similarities between these molecules based on one of these factors, the use of all three data points can be utilized to distinguish and differentiate biochemicals. More than 3300 commercially available purified standard compounds have been acquired and registered into LIMS for analysis on all platforms for determination of their analytical characteristics. Additional mass spectral entries have been created for structurally unnamed biochemicals, which have been identified by virtue of their recurrent nature (both chromatographic and mass spectral). These compounds have the potential to be identified by future acquisition of a matching purified standard or by classical structural analysis.

Curation: A variety of curation procedures were carried out to ensure that a high-quality data set was made available for statistical analysis and data interpretation. The QC and curation processes were designed to ensure accurate and consistent identification of true chemical entities, and to remove those representing system artifacts, mis-assignments, and background noise. Metabolon data analysts use proprietary visualization and interpretation software to confirm the consistency of peak identification among the various samples. Library matches for each compound were checked for each sample and corrected if necessary.

Metabolite Quantification and Data Normalization: Peaks were quantified using area-under-the-curve. For studies spanning multiple days, a data normalization step was performed to correct variation resulting from instrument inter-day tuning differences. Essentially, each compound was corrected in run-day blocks by registering the medians to equal one (1.00) and normalizing each data point proportionately (termed the "block correction"). For studies that did not require more than one day of analysis, no normalization is necessary, other than for purposes of data visualization. Metabolite levels were normalized to total protein as determined by Bradford assay.

Statistical Calculations: Welch's two-sample *t*-test was performed in ArrayStudio on log transformed data.

1.5 Atomic Force Microscopy

1.5.1 Bio-functionalization of the AFM cantilevers

AFM cantilevers (Spring constant ~ 0.02 N/m; OMCL-TR400PB, Olympus/Asylum Research) were biofunctionalized by sialylic-Lewis^x (sLe^x) as previously described (Micic et al., 1999; Askarova et al., 2013). Briefly, cantilevers were cleaned by immersion in acetone (Sigma) for 1 min, followed by 15 min of ultraviolet irradiation and then incubated in 0.5 mg/ml biotin-BSA (Sigma) at 37°C overnight. The biotin-BSA cantilevers were rinsed several times with PBS then fixed in 20% glutaraldehyde (Sigma) for 30 min followed by treatment with 0.5 mg/ml avidin (Sigma) for 5 min. The cantilevers were washed again several times and incubated with 0.5 mg/ml biotin-sLe^x (Glycotech, Gaithersburg, MD) for 5 min.

1.5.2 Measurements of cell stiffness, membrane tethering force and adhesion probability

MFP-3D-BIO AFM (Oxford Instruments Asylum Research, Santa Barbara, CA) was employed. In AFM force mode, the piezotranducer (PZT) is set to drive the cantilever to approach, touch, make an indentation of the cell, and retract from the cell over a predefined distance in the optical axis perpendicular to the cell surface, which can be identified from the force curve. The force curve is generated from the recorded vertical-axis movement of the PZT and the deflection of the cantilever with a known dimension and a spring constant. The sudden release of force occurred at the rupture of a membrane tether is used as a measure of membrane tethering force (F_{mtf}) through the bonding between selectins and sLe^x calculated by multiplying the spring constant of the cantilever with the deflection height associated with a membrane tether rupture. However, adhesion events cannot be obtained from all force curves. Therefore, adhesion probability is calculated by dividing the number of curves with adhesion events by the total number of curves. To measure the stiffness of the cell, the part of the force curve representing cell indentation is fitted by the Hertz model to calculate the Young's modulus (i.e., the stiffness) of the cell. At least 50 curves were analyzed per n and averaged for statistical analysis by paired *t*-test due to nature of the technique.

1.6 Leukocyte Adhesion Assay

Leukocytes were isolated as previously described (Lutz et al., 2017). Briefly, spleens from 3-week-old male *APOE3*-targeted replacement mice were harvested and passed through a 40 µm filter. Leukocytes were treated with Ammonium-Chloride-Potassium buffer (150 mM NH_4Cl, 10 mM $KHCO_3$, 0.1 mM EDTA, pH 7.2-7.4) to remove red blood cells and then resuspended in imaging buffer (HBSS, 4.2 mM $NaCO_3$, 10 mM HEPES) supplemented with 1% FBS. 2 x 10^5 Leukocytes were incubated with brain endothelial cells for 1 h at 37°C. Cells were then washed 3 times in imaging buffer, fixed with 4% PFA and immunostained for CD45 (1:500) and ZO-1 (1:250). Images were captured with a Leica DMI8 epifluorescence microscope at 20X magnification, and CD45$^+$ cells were quantified using Particle Analysis in Fiji.

Supplementary Table 1: Detailed materials and methods

Figure Panel	Assay Kit/Antibodies	Vendor	Catalog Number	Cell Seeding: Cortices per Well	Cell Seeding: Growth Surface	Sample Preparation, Experimental Specifications and/or Deviations from Manufacturer Protocols
1A & 1E	TEER	-	-	$^{1}/_{6}$	96w10idf	Electrical cell-substrate impedance sensing was performed on an ECIS® ZΘ (Applied Biophysics). Resistance and capacitance were monitored across measuring frequencies of 1000, 2000, 4000 and 64000 Hz starting at the time of puromycin addition. For TEER, background resistance values (i.e., measurements from empty wells) were subtracted.
1B	DAPI	Thermo	62248	$^{1}/_{6}$	96 Well Plate	Cells fixed at 100% confluence were labeled with DAPI and imaged at 10X magnification on an ImageXpress Micro (Molecular Devices). Total cell count per well was obtained with the Count Nuclei module in MetaXpress software (Molecular Devices).
1C	5’-Bromo-2’-deoxyuridine	Roche Diagnostics	10280879001	$^{1}/_{6}$	96 Well Plate	A 10 mM stock of BrdU in water was diluted to 10 µM in complete culture medium and used to replace the existing culture medium when the cells reached confluence. After 24 h of BrdU labeling, cells were washed 5 times in PBS, fixed in 3% PFA and permeabilized according to the ICC protocol detailed above in Supplementary Materials and Methods. Following permeabilization cells were washed 3 times in PBS then incubated in 1 M HCl for 10 min at room temperature followed by a 30 min incubation in 0.1 M sodium tetraborate, pH 8.5 at room temperature. Following 3 washes with PBS, cells were labeled immunocytochemically following the remainder of the protocol detailed above. Following immunocytochemical labeling, the cells were imaged at 10X magnification on an ImageXpress Micro (Molecular Devices). The percentage of $BrdU^{+}$ cells was determined using the Cell Scoring module in MetaXpress (Molecular Devices).
	BrdU Monoclonal Antibody (MoBU-1)	Invitrogen	B35128			
	Donkey anti-Mouse IgG (H+L) Highly Cross-Adsorbed Secondary Antibody, Alexa Fluor 488	Invitrogen	A21202			
	DAPI	Thermo	62248			
1D	Anti-Claudin 5, C-Terminal antibody	Sigma-Aldrich	SAB4502981	$^{1}/_{4}$	12 Well Chamber Slide	Cells were fixed with 70% ethanol and probed as detailed above in Supplementary Materials and Methods.
	Occludin Monoclonal Antibody (OC-3F10)	Invitrogen	33-1500			
	DAPI	Thermo	62248			
	Donkey anti-Rabbit IgG (H+L) Highly Cross-Adsorbed Secondary Antibody, Alexa Fluor 488	Invitrogen	A21206			
	Donkey anti-Mouse IgG (H+L) Highly Cross-Adsorbed Secondary Antibody, Alexa Fluor 594	Invitrogen	A21203			

2A	BaseScope™ Reagent Kit v2 - RED	Advanced Cell Diagnostics	323900	$^{1}/_{4}$	12 Well Chamber Slide	Cells were prepared and pretreated according to technical note MK-50 010. Assay was performed immediately following fixation according to Chapter 4 of the user manual 323900-USM. The following modifications were made:<br>Dehydration/rehydration steps were omitted as assay was performed immediately after fixation.<br>Protease III was diluted 1:15 in PBS and incubated for 10 min.<br>Human *APOE* BaseScope Probe: BA-Hs-APOE-No-XMm-1zz-st.<br>PBS was substituted for all wash steps calling for distilled water.<br>Immunocytochemistry for ZO-1 and DAPI was performed following signal detection, and confocal images were captured as Z-stacks on a Zeiss LSM710 at 40X magnification with an optical zoom of 1.3X.
	Anti-ZO-1 Polyclonal Antibody	Invitrogen	61-7300			
	Donkey anti-Rabbit IgG (H+L) Highly Cross-Adsorbed Secondary Antibody, Alexa Fluor 488	Invitrogen	A21206			
	DAPI	Thermo	62248			
2B	Anti-Apolipoprotein E Antibody	EMD Millipore	AB947	$^{1}/_{4}$	12 Well Chamber Slide	Cells were fixed overnight with 3% PFA and probed as detailed above in Supplementary Materials and Methods.
	Anti-ZO-1 Polyclonal Antibody	Invitrogen	61-7300			
	DAPI	Thermo	62248			
	Donkey anti-Goat IgG (H+L) Cross-Adsorbed Secondary Antibody, Alexa Fluor 594	Invitrogen	A11058			
	Donkey anti-Rabbit IgG (H+L) Highly Cross-Adsorbed Secondary Antibody, Alexa Fluor 488	Invitrogen	A21206			
2C	Anti-Apolipoprotein E Antibody	EMD Millipore	AB947	7	T25 Flask	Lysates were prepared and analyzed by western blot as described above in Supplementary Materials and Methods. Primary antibody incubations were as follows:<br><br>ApoE: 1:1000, overnight at 4°C<br>Actin: 1:50,000, 2 h at room temperature<br>Secondary antibodies: 1:10,000, 45 min at room temperature<br>ApoE was normalized to β-Actin.
	β-Actin (8H10D10) Mouse mAb #3700	Cell Signaling	3700S			
	IRDye® 800CW Donkey anti-Rabbit IgG Secondary Antibody	LI-COR Biosciences	926-32213			
	IRDye® 680RD Goat anti-Mouse IgG Secondary Antibody	LI-COR Biosciences	926-68070			
2D	Anti-Apolipoprotein E Antibody	EMD Millipore	AB947	7	T25 Flask	Media was collected, prepared, and analyzed by western blot as described above in Supplementary Materials and Methods. Primary antibody incubations were as follows:<br><br>ApoE: 1:1000, overnight at 4°C<br>Secondary antibodies: 1:10,000, 45 min at room temperature<br>ApoE was normalized to cell count.
	β-Actin (8H10D10) Mouse mAb #3700	Cell Signaling	3700S			
	IRDye® 800CW Donkey anti-Rabbit IgG Secondary Antibody	LI-COR Biosciences	926-32213			
	IRDye® 680RD Goat anti-Mouse IgG Secondary Antibody	LI-COR Biosciences	926-68070			

2E	Anti-Apolipoprotein E Antibody (Capture)	EMD Millipore	AB947	$^1/_6$	96 Well Plate	ApoE was measured in media diluted 1:16 by ELISA as previously described in (Tai et al., 2014; Marottoli et al., 2017). ApoE levels were normalized to cell count.
	Anti-Apolipoprotein E Antibody (Detection)	Meridian Life Science	K74180B			
	Streptavidin Poly-HRP40 Conjugate (Secondary)	Fitzgerald Industries	65R-S104PHRP			
3A-B	miRNeasy Mini Kit	Qiagen	217004	1	12 Well Plate	No modifications to manufacturer protocol.
4A	CellTiter®-Glo 2.0 Cell Viability Assay	Promega	G9241	$^1/_6$	96 Well Plate	No modifications were made to manufacturer protocol.
4B-C	Seahorse XF Real-Time ATP Rate Assay Kit	Agilent	103591-100	$^1/_6$	Seahorse XF96 V3 PS Cell Culture Microplate	Assay was performed according to the manufacturer's protocol with the following modifications:<br><br>Brain endothelial cells were isolated directly onto Seahorse XF96 V3 PS Microplates and assayed at passage 0.<br>Assay Medium: Seahorse XF RPMI Medium, pH 7.4 (Agilent, 103576-100) supplemented with 5.5 mM Seahorse XF Glucose (Agilent, 103577-100), 1.0 mM XF Seahorse Pyruvate (Agilent, 103578-100) and 10 mM XF Seahorse Glutamine (Agilent, 103579-100).
4D	Glucose Uptake-Glo	Promega	J1341	$^1/_6$	96 Well Plate	No modifications were made to the manufacturer's protocol. One technical replicate was used for a surrogate cell count to normalize data.
4E	Lactate-Glo	Promega	J5021	$^1/_6$	96 Well Plate	Assay performed according to the manufacturer's protocol with media (1:80). A technical replicate was used for surrogate cell count.
4F	TMRE-Mitochondrial Membrane Potential Assay Kit	abcam	ab113852	$^1/_6$	96 Well Plate	Assay was performed according to the manufacturer's protocol with the following modifications to maintain the conditioned extracellular environment throughout the duration of the experiment:<br>2.5 µM TMRE (10X) was prepared in fresh complete culture media.<br>TMRE was diluted individually to a final working concentration of 250 nM by adding 12 µl of the 10X stock 108 ml of media from each well in a separate dilution plate.<br>The remaining media was replaced with 100 µl of the 250 nM TMRE such that each well received its own media for the assay.<br>Nuclei were stained by spiking 4 µl of Hoechst33342 into each well. (Invitrogen, R37605).<br>Incubation time was 30 min at 37°C.<br>Fluorescence (ex/em = 549/575 nm) was measured in fresh 0.2% BSA in PBS with a SpectraMax i3x multi-mode plate reader (Molecular Devices). Cell count was obtained with the Count Nuclei module MetaXpress software (Molecular Devices) for normalization.

4G	JC-1 Mitochondrial Membrane Potential Assay Kit	Cayman Chemical	10009172	$^{1}/_{6}$	96 Well Plate	No modifications were made to the manufacturer's protocol. Fluorescence (Aggregates: ex/em = 535/595 nm; Monomers: ex/em = 485/535 nm) with a SpectraMax i3x multi-mode plate reader (Molecular Devices).
4H	MitoCheck® Citrate Synthase Activity Assay Kit	Cayman Chemical	701040	4	6 Well Plate	Cell culture lysates were prepared from 6 well plates. Cells were washed with 1 ml of PBS twice. One well at a time, cells were scraped in 110 µl of the supplied assay buffer. Samples were sonicated on ice for two 15 s pulses at 20% amplitude then centrifuged at 13,000 × *g* for 15 min at 4°C and the supernatant was transferred to a new tube.<br><br>Assay was performed in the supplied 96 well plate format according to the manufacturer's protocol without modification. Citrate synthase activity was normalized to protein obtained with a Pierce™ BCA Protein Assay Kit (Thermo Scientific, 23225).
4I	Total OXPHOS Rodent WB Antibody Cocktail	abcam	45-8099	7	T25	Lysates were analyzed by western blot as described for Figure 2C.
4J	NAD/NADH-Glo™ Assay	Promega	G9071	$^{1}/_{6}$	96 Well Plate	Assay was performed for measuring NAD^+ and NADH individually according to the manufacturer's technical manual with the following modifications:<br><br>Cells were lysed in 0.2N NaOH + 2% DTAB<br>Plate was incubated at room temperature for 60 min before reading luminescence.
5A	DCFDA/H2DCFDA - Cellular ROS Assay	abcam	ab113851	$^{1}/_{6}$	96 Well Plate	Assay was performed according to manufacturer's protocol with 25 µM DCFDA and fluorescence (ex/em = 485/535 nm) was measured with a SpectraMax i3x multi-mode plate reader (Molecular Devices). Following assay, cells were fixed and stained with DAPI to normalize results to cell count.
5B	ROS-Glo™ H_2O_2 Assay	Promega	G8820	$^{1}/_{6}$	96 Well Plate	No modifications were made to the manufacturer's protocol. One technical replicate was used for a surrogate cell count to normalize data.
5C	DCFDA/H2DCFDA - Cellular ROS Assay	abcam	ab113851	$^{1}/_{6}$	96 Well Plate	Assay was performed as described for Figure 5A with the exception that cells were treated with H_2O_2 following the removal of the DCFDA.
5D	Peroxynitrite Assay Kit (Cell-based)	abcam	ab233468	$^{1}/_{6}$	96 Well Plate	No modifications were made to the manufacturer's protocol. Fluorescence (ex/em = 490/530 nm) was measured with a SpectraMax i3x multi-mode plate reader (Molecular Devices). Following assay, cells were fixed and stained with DAPI to normalize results to cell count.

5E	Cellular Superoxide Detection Assay Kit	abcam	ab139477	$^{1}/_{6}$	Ibidi Culture Insert 4-Well µ-Dish	Assay was performed according to the manufacturer's protocol except the cells were washed and counterstained with Hoechst33342 as described for Figure 4F. Images were captured as Z-stacks on a Zeiss LSM710 at 63X magnification with an optical zoom of 1.4X. Integrated density was measured with ImageJ as follows: Individual Z-plane images were auto local thresholded using the Phansalkar setting. Z-projections were created with the "Sum Slices" setting. Z-projections were then thresholded with the Minimum setting and quantified by the "Analyze Particles" feature with particle size limits set from 0-infinity. Integrated densities were normalized to cell number.
5F	MitoSOX™ Red Mitochondrial Superoxide Indicator	Invitrogen	M36008	$^{1}/_{6}$	Ibidi Culture Insert 4-Well µ-Dish	Manufacturer's protocol MP 36008 (Revised: 03-March-2005) was followed except the cells were washed and counterstained with Hoechst33342 as described for Figure 4F. Images were captured as Z-stacks on a Zeiss LSM710 at 63X magnification with an optical zoom of 1.6X. Integrated density was measured with as follows: Individual Z-plane images were auto local thresholded using the Otsu setting. Z-projections were created with the "Sum Slices" setting. Z-projections were then thresholded with the RenyiEntropy setting and quantified by the "Analyze Particles" feature with particle size limits set from 0-3. Integrated densities were normalized to cell number.
5G	Mitochondrial Hydroxyl Radical Detection Kit	abcam	ab219931	$^{1}/_{6}$	Ibidi Culture Insert 4-Well µ-Dish	Manufacturer's protocol (Version 1a; Last updated 6 December 2018) was followed with the exception that the cells were counterstained with Hoechst33342 as described for Figure 4F. Images were captured as Z-stacks on a Zeiss LSM710 at 63X magnification with an optical zoom of 1.4X. Integrated density was measured as described for Figure 5F.
5H	Heme	Sigma-Aldrich	MAK316	4	6 Well Plate	Cell culture lysates were prepared from 6 well plates. Cells were washed with 1 ml of PBS twice. One well at a time, cells were scraped in 110 µl of 2 mM EDTA in PBS. Samples were sonicated on ice for two 15 s pulses at 20% amplitude then centrifuged at 13,000 × *g* for 15 min at 4°C and the supernatant was transferred to a new tube.<br>Assay was performed in 96 well plate format according to the manufacturer's technical bulletin without modification. Heme concentration was normalized to protein obtained with a Pierce™ BCA Protein Assay Kit (Thermo Scientific, 23225).<br>Remaining lysate sample volume was used to measure bilirubin (Figure 5I). Media collected from this experiment was used to measure LDH (Figure 7F).
5I	Bilirubin	Novus Biologicals	NBP2-69939	4	6 Well Plate	Sample lysates were prepared as in Figure 5H. No modifications were made to the manufacturer's protocol. Unknown bilirubin sample concentrations were calculated from a four-parameter logistic curve on a log-log graph using GraphPad Prism 9.

5J	GSH/GSSG-Glo™ Assay	Promega	V6611	$^{1}/_{6}$	96 Well Plate	No modifications were made the manufacturer protocol.
6A	8-oxo-dG	Trevigen	4354-MC-050	$^{1}/_{6}$	96 Well Plate	Immunocytochemistry was performed according to product datasheet IFU0146 Rev 3 with the exception that non-specific binding was blocked with 4% BSA in PBS instead of 5% normal goat serum. Pseudo Z-stack were captured at 10X magnification on an ImageXpress Micro (Molecular Devices) and the integrated density of 8-oxo-dG signal was quantified with the Cell Scoring module in MetaXpress software (Molecular Devices) and normalized to cell count.
6B	Anti-phospho-histone H2A.X (SER139)<br><br>DAPI	Sigma-Aldrich<br><br>Thermo	ZRB05636<br><br>62248	$^{1}/_{6}$	96 Well Plate	Cells were fixed overnight with 3% PFA and probed as detailed above in Supplementary Materials and Methods. Following immunocytochemical labeling, the cells were imaged at 10X magnification on an ImageXpress Micro (Molecular Devices). The percentage of γH2A.X^{+} cells was determined using the Cell Scoring module in MetaXpress software (Molecular Devices).
6C	S-Glutathionylated Protein Detection Kit	Cayman Chemical	10010721	$^{1}/_{6}$	96 Well Plate	Assay was performed to the manufacturer’s protocol and fluorescence (ex/em = 488/535 nm) with a SpectraMax i3x multi-mode plate reader (Molecular Devices). Following the assay, immunocytochemistry was performed for both ZO-1 and DAPI and results were normalized to cell count.
6D	Protein Carbonylation Colorimetric Assay Kit	Cayman Chemical	10005020	4	6 Well Plate	Cell culture lysates were prepared from 6 well plates. Cells were washed once with 1 ml of PBS, pH 5.95. One well at a time, cells were scraped in 110 µl of 2 mM EDTA in PBS, pH 5.95. Samples were sonicated on ice for two 15 s pulses at 20% amplitude then centrifuged at 13,000 × *g* for 15 min at 4 °C and the supernatant was transferred to a new tube. 10% streptazocin in ultrapure water was added for a final concentration of 1% streptazocin and samples were incubated for 15 min at room temperature. Lysates were centrifuged at 13,000 × *g* for 15 min at 4°C. Following sample preparation, the following modifications were made to the manufacturer’s protocol:<br>All centrifuge steps were performed at 13,000 × *g* at 4°C.<br>One volume (equal to recovered sample volume) of DNPH per sample.<br>One volume (recovered sample volume plus DNPH) of 20% TCA solution was added per sample.<br>Ethanol/Ethyl Acetate washes were performed in a volume of 0.5 ml.<br>Pellet was resuspended in 110 µl of guanidine HCl after final wash.<br>90 µl of sample supernatant was transferred to the assay plate and measured at 375 nm.<br>The remaining sample supernatant was used for a BCA protein assay.

6E	Premo™ Autophagy Tandem Sensor RFP-GFP-LC3B Kit	Molecular Probes	P36239	$^{1}/_{6}$	Ibidi Culture Insert 4-Well µ-Dish	Assay was performed according to the manufacturer's protocol. Cells were loaded with 32 PPC LC3B reagent and incubated for 20 h.<br><br>Cells were counterstained with Hoechst33342 as described for Figure 4F and imaged in complete phenol-free complete media. Images were captured as Z-stacks on a Zeiss LSM710 at 63X magnification with an optical zoom of 1.4X. Z-projections were created with the "Sum Slices" setting. Z-projections were then auto local thresholded using the Phansalkar setting. LC3B-GFP- and LC3B-RFP-positive autophagosomes were quantified by the "Analyze Particles" feature with particle size limits set from 0.1-3. LC3B-GFP- and LC3B-RFP-positive autophagosome counts were normalized to cell number.
6F	Proteosome-Glo	Promega	G1180	$^{1}/_{6}$	96 Well Plate	No modifications were made to the manufacturer's protocol. One technical replicate was used for a surrogate cell count to normalize data.
6G	Image-iT™ Lipid Peroxidation Kit BODIPY	Invitrogen	C10445	$^{1}/_{6}$	Ibidi Culture Insert 4-Well µ-Dish	Manufacturer's protocol MAN0006855 was followed with the exception that all wash steps were performed as follows:<br>Cells were washed twice with HBSS (with Ca^{2+} and Mg^{2+}) followed by a final wash in Live Cell Imaging Solution (Invitrogen, A14291DJ).<br>Nuclei were stained with Hoechst33342 (Invitrogen, R37605).<br>The final wash was replaced with fresh Live Cell Imaging Solution for imaging.<br>Images were captured as Z-stacks on a Zeiss LSM710 at 63X magnification with an optical zoom of 1.4X. Integrated density was measured with ImageJ for both red and green channels with as follows: Individual Z-plane images were auto local thresholded using the Bernsen setting. Z-projections were created with the "Sum Slices" setting. Z-projections were then thresholded with the RenyiEntropy setting and quantified by the "Analyze Particles" feature with particle size limits set from 0-infinity. The ratio of integrated densities at 590 nm to 510 nm were then calculated.

6H	TBARS Assay Kit	Cayman Chemical	10009055	4	6 Well	Cell culture lysates were prepared from 6 well plates. Cells were washed twice with PBS. One well at a time, cells were scraped in 110 µl RIPA buffer. Samples were sonicated on ice for two 15 s pulses at 20% amplitude then centrifuged at 13,000 × *g* for 15 min at 4 °C and the supernatant was transferred to a new tube.<br>50 µl of sample was assayed neat and normalized to protein. The following modifications were made to the manufacturer's protocol for colorimetric detection:<br>50 µl of neat sample was assayed.<br>Volumes for all reagents throughout protocol were reduced by half to accommodate 50 ml sample volume.<br>Assay results were normalized to protein obtained with a Pierce™ BCA Protein Assay Kit (Thermo Scientific, 23225). The remaining sample was assayed for 4-HNE (Figure 6I).
6I	Lipid Peroxidation (4-HNE) Assay Kit	abcam	ab238538	4	6 Well	50 µl of sample (preparation described above for Figure 6H) was assayed neat according to the manufacturer's protocol and normalized to protein.
7B	Phosphatidylcholine Assay Kit	Sigma-Aldrich	MAK049	4	6 Well Plate	Cell culture lysates were prepared from 6 well plates. Cells were washed twice with PBS. One well at a time, cells were scraped in 110 µl PC Assay Buffer. Samples were sonicated on ice for two 15 s pulses at 20% amplitude then centrifuged at 13,000 × *g* for 15 min at 4 °C and the supernatant was transferred to a new tube. Samples were diluted 1:2 and assayed according to the manufacturer's bulletin with colorimetric detection. Assay results were normalized to protein obtained with a Pierce™ BCA Protein Assay Kit (Thermo Scientific, 23225).
7C	Cholesterol-Glo	Promega	J3190	$^{1}/_{6}$	96 Well Plate	Assay was performed according to the manufacturer's protocol for both cell lysates and neat media. One technical replicate was used for a surrogate cell count to normalize data.
7D	Triglyceride-Glo	Promega	J3160	$^{1}/_{6}$	96 Well Plate	Assay was performed according to the manufacturer's protocol for both cell lysates and neat media. One technical replicate was used for a surrogate cell count to normalize data.
7E	AFM Cell Stiffness	-	-	$^{1}/_{4}$	12 Well Chamber Slide	Protocol is detailed above in Supplementary Materials and Methods.
7F	LDH-Glo™ Cytotoxicity Assay	Promega	J2380	4	6 Well Plate	Assay was performed according to the manufacturer's protocol with media diluted 1:50.

8A	MILLIPLEX MAP Mouse Cytokine/Chemokine Magnetic Bead Panel - Immunology Multiplex Assay	EMD Millipore	MCYTOMAG-70K	$^{1}/_{6}$	96 Well Plate	Media was assayed neat according to the manufacturer's protocol. Samples were incubated overnight at 4°C plus and additional hour at room temperature.
8B	AFM Adhesion Probability	-	-	$^{1}/_{4}$	12 Well Chamber Slide	Protocol is detailed above in Supplementary Materials and Methods.
8C	AFM Membrane Tethering Force	-	-	$^{1}/_{4}$	12 Well Chamber Slide	Protocol is detailed above in Supplementary Materials and Methods.
8D	ZO-1 Monoclonal Antibody (ZO1-1A12)	Invitrogen	33-9100	$^{1}/_{4}$	12 Well Chamber Slide	Protocol is detailed above in Supplementary Materials and Methods.
	Anti-CD45 Antibody, clone IBL-5/25	EMD Millipore	05-1416			
	Donkey anti-Mouse IgG (H+L) Highly Cross-Adsorbed Secondary Antibody, Alexa Fluor 647	Invitrogen	A31571			
	Donkey anti-Rat IgG (H+L) Highly Cross-Adsorbed Secondary Antibody, Alexa Fluor 594	Invitrogen	A21209			
9A-B	LPS from E. coli O8:K27 (S-form)	Innaxon Biosciences	IAX-100-006-5001	$^{1}/_{6}$	96w10idf	LPS was spiked into the media on day 6 post isolation, resulting in final concentrations of 0, 0.1, 1, 10 or 100 µg/ml. TEER was measured as described for Figure 1A and 1E for the subsequent 24 h following addition of LPS. TEER was normalized to t = 0 h values (i.e., before addition of LPS).
9C	LPS from E. coli O8:K27 (S-form)	Innaxon Biosciences	IAX-100-006-5001	$^{1}/_{4}$	12 Well Chamber Slide	LPS was spiked into the media on day 6 resulting in final concentrations of 0, 1, 10 or 100 µg/ml. Immunocytochemistry was performed as in Figure 1D.
	Anti-Claudin 5, C-Terminal antibody	Sigma-Aldrich	SAB4502981			
	Occludin Monoclonal Antibody (OC-3F10)	Invitrogen	33-1500			
	DAPI	Thermo	62248			
	Donkey anti-Rabbit IgG (H+L) Highly Cross-Adsorbed Secondary Antibody, Alexa Fluor 488	Invitrogen	A21206			
	Donkey anti-Mouse IgG (H+L) Highly Cross-Adsorbed Secondary Antibody, Alexa Fluor 594	Invitrogen	A21203			

9D	LPS from E. coli O8:K27 (S-form)	Innaxon Biosciences	IAX-100-006-5001	$^{1}/_{6}$	96 Well Plate	LPS was spiked into media as described in Figure 8A to a final concentration of 100 ng/ml 24 h prior to assay. Assay was performed as described for Figure 8A.
	MILLIPLEX MAP Mouse Cytokine/Chemokine Magnetic Bead Panel - Immunology Multiplex Assay	EMD Millipore	MCYTOMAG-70K			
9E	LPS from E. coli O8:K27 (S-form)	Innaxon Biosciences	IAX-100-006-5001	$^{1}/_{4}$	12 Well Chamber Slide	LPS was spiked into media as described in Figure 8B to a final concentration of 100 ng/ml 24 h prior to assay. Assay was performed as described for Figure 8D.
	ZO-1 Monoclonal Antibody (ZO1-1A12)	Invitrogen	33-9100			
	Anti-CD45 Antibody, clone IBL-5/25	EMD Millipore	05-1416			
	Donkey anti-Mouse IgG (H+L) Highly Cross-Adsorbed Secondary Antibody, Alexa Fluor 647	Invitrogen	A31571			
	Donkey anti-Rat IgG (H+L) Highly Cross-Adsorbed Secondary Antibody, Alexa Fluor 594	Invitrogen	A21209			
10A	SR 9009 (Rev-Erbα/β agonist)	Tocris Bioscience	5855	$^{1}/_{6}$	96 Well Plate	Cells were incubated with 5 µM SR9009 for 48 h leading up to the assay. Assay was performed as described for Figure 2E.
	Anti-Apolipoprotein E Antibody (Capture)	EMD Millipore	AB947			
	Anti-Apolipoprotein E Antibody (Detection)	Meridian Life Science	K74180B			
	Streptavidin Poly-HRP40 Conjugate (Secondary)	Fitzgerald Industries	65R-S104PHRP			
10B	SR 9009 (Rev-Erbα/β agonist)	Tocris Bioscience	5855	$^{1}/_{6}$	Ibidi Culture Insert 4-Well m-Dish	Cells were incubated with 5 µM SR9009 for 48 h leading up to the assay. Assay was performed as described for Figure 5F.
	MitoSOX	Invitrogen	M36008			
10C	SR 9009 (Rev-Erbα/β agonist)	Tocris Bioscience	5855	$^{1}/_{4}$	12 Well Chamber Slide	Cells were incubated with 5 µM SR9009 for 48 h leading up to the assay. Assay was performed as described for Figure 7E.
10D	SR 9009 (Rev-Erbα/β agonist)	Tocris Bioscience	5855	$^{1}/_{6}$	96 Well Plate	Cells were incubated with 5 µM SR9009 for 48 h leading up to the assay. Assay was performed as described for Figure 8A.
	MILLIPLEX MAP Mouse Cytokine/Chemokine Magnetic Bead Panel - Immunology Multiplex Assay	EMD Millipore	MCYTOMAG-70K			

10E	SR 9009 (Rev-Erbα/β agonist)	Tocris Bioscience	5855	1/4	12 Well Chamber Slide	Cells were incubated with 5 µM SR9009 for 48 h leading up to the assay. Assay was performed as described for Figure 8B.
10F	SR 9009 (Rev-Erbα/β agonist)	Tocris Bioscience	5855	1/4	12 Well Chamber Slide	Cells were incubated with 5 µM SR9009 for 48 h leading up to the assay. Assay was performed as described for Figure 8C.
10G	SR 9009 (Rev-Erbα/β agonist)	Tocris Bioscience	5855	1/4	12 Well Chamber Slide	Cells were incubated with 5 µM SR9009 for 48 h leading up to the assay. Assay was performed as described for Figure 8D.
	ZO-1 Monoclonal Antibody (ZO1-1A12)	Invitrogen	33-9100			
	Anti-CD45 Antibody, clone IBL-5/25	EMD Millipore	05-1416			
	Donkey anti-Mouse IgG (H+L) Highly Cross-Adsorbed Secondary Antibody, Alexa Fluor 647	Invitrogen	A31571			
	Donkey anti-Rat IgG (H+L) Highly Cross-Adsorbed Secondary Antibody, Alexa Fluor 594	Invitrogen	A21209			
11A-B	SR 9009 (Rev-Erbα/β agonist)	Tocris Bioscience	5855	1/6	96w10idf	Cells were incubated with 5 µM SR9009 for 48 h leading up to the assay. LPS was spiked into media as described in Figure 9B resulting in final concentration 100 µg/ml. TEER was measured as described for Figure 1A and 1E for the subsequent 24 h following addition of LPS.
	LPS from E. coli O8:K27 (S-form)	Innaxon Biosciences	IAX-100-006-5001			
11C	SR 9009 (Rev-Erbα/β agonist)	Tocris Bioscience	5855	1/4	12 Well Chamber Slide	Cells were incubated with 5 µM SR9009 for 48 h leading up to the assay. LPS was spiked into media as described in Figure 9B resulting in final concentrations of 100 µg/ml. Immunocytochemistry was performed as in Figure 1D.
	LPS from E. coli O8:K27 (S-form)	Innaxon Biosciences	IAX-100-006-5001			
	Anti-Claudin 5, C-Terminal antibody	Sigma-Aldrich	SAB4502981			
	Occludin Monoclonal Antibody (OC-3F10)	Invitrogen	33-1500			
	DAPI	Thermo	62248			
	Donkey anti-Rabbit IgG (H+L) Highly Cross-Adsorbed Secondary Antibody, Alexa Fluor 488	Invitrogen	A21206			
	Donkey anti-Mouse IgG (H+L) Highly Cross-Adsorbed Secondary Antibody, Alexa Fluor 594	Invitrogen	A21203			

11D	SR 9009 (Rev-Erbα/β agonist)	Tocris Bioscience	5855	$^{1}/_{6}$	96 Well Plate	Cells were incubated with 5 µM SR9009 for 48 h leading up to the assay. LPS was spiked into media to a final concentration of 100 ng/ml 24 h prior to assay. Assay was performed as described for Figure 8A.
	LPS from E. coli O8:K27 (S-form)	Innaxon Biosciences	IAX-100-006-5001			
	MILLIPLEX MAP Mouse Cytokine/Chemokine Magnetic Bead Panel - Immunology Multiplex Assay	EMD Millipore	MCYTOMAG-70K			
11E	SR 9009 (Rev-Erbα/β agonist)	Tocris Bioscience	5855	$^{1}/_{4}$	12 Well Chamber Slide	Cells were incubated with 5 µM SR9009 for 48 h leading up to the assay. LPS was spiked into media to a final concentration of 100 ng/ml 24 h prior to assay. Assay was performed as described for Figure 7E.
	LPS from E. coli O8:K27 (S-form)	Innaxon Biosciences	IAX-100-006-5001			

2 Supplementary Results

Supplementary Table 2: Functional clusters of differentially expressed genes in *APOE4* versus *APOE3* brain endothelial cells

**Metabolism**	
Lipoprotein	
Up: Clu, Pon3, Apod, Abcg1, Ldlr, Lmf2	*Down:* Gpihbp1, APOE, Pltp, Lrp4, Gpr146, Sphk2
Oxidative Stress	
Up: Erfe, Lcn2, Gstt3, Hmgb2, Prdx4, Gpx7, Hmgb3, Gstm2, Glrx, Alas1, Cp, Nqo2, Psip1, Sh3bgrl3	*Down*: Blvrb, Ager, Slc22a17, Gstp1, Txnip, Gclc, Txnrd3, Secisbp2, Slc25a38, Gstm1, Ppox, Gstt2, Hmox1, Mafg, Slc40a1
Mitochondrial	
Up: Selenoh, Mtfr2, AW112010, Rad51c, Ifi27, Cep89, mt-Nd2, Cmc2, mt-Nd5, Ethe1, Mrps12, Mrpl51, Slc25a24, mt-Nd4, mt-Co1, mt-Cytb, mt-Nd1, Bak1, Slc29a1, Yme1l1, Slc25a4	*Down:* Rsad1, Osgin1, Mettl17, Pusl1, Lars2, Slc25a27, Klc4, Aars2, Ucp2, Ngrn, Phykpl, Tk2, Atp5sl, Ndufa6, Pink1, Pdk2, Polrmt, Mtfr1l, Timm44
Plasma Membrane/vesicle trafficking	
Up: Enpp6, Kif20a, Kif20b, Kifc5b, Sgip1, Kif15, Kif23, Mboat1, Ston1, Plscr1, Plscr1, Exoc3l4, Cpm	*Down:* Kifc2, Snca, Kif26a, Gabarapl1, Dennd6b, Tbc1d17, Rab6b, Vamp2, Atp9a, Galnt15, Fcho2, Sh3bp4, Vps11, Cog8, Tpst2, Dennd3, Use1, Unc13b, Dopey2, Chka, Rabac1, Clip1, Palm, Napa
Glucose	
Up: Dok1, Egln3, Htra1, Nuak2, Pask, Grb14, Igf2r, Tbc1d1, Fuca2, C1qtnf6, Slc37a3, Tulp4, Hif1a	*Down:* Igfbp5, Pygm, Irx3, Igfbp4, Gigyf1, Slc45a4, Tbc1d8, Isyna1, Hif3a, Snrk, Preb, Igf1r, Srsf5, Slc16a1, Slc50a1, Sorbs1, Aldoa
Fatty Acid	
Up: Scd1, Hacd4, Fads2, Cpt1c, Cyp39a1, Scd2, Soat1, Acadsb, Acaca, Acot9	*Down:* Hmgcs2, Cyp2d22, Pnpla7, Lipe, Acot2, Slc16a6, Hmgcl, Acaa2, Eci1, Zfp69, Arfrp1, Acaa1a, Pank4, Nceh1, Pnpla6, Atg2a, MARCH6
Amino Acid	
Up: Ahcy, Oat, Dhfr, Sat1, Oxct1	*Down:* Slc7a8, Lao1, Hhipl1, Slc7a5, Slc3a2, Mccc1, Aldh6a1, Sfxn3, Gatsl3, Impact, Mat2a
Unfolded Protein Response/Endoplasmic Reticulum	
Up: Fkbp11, Hspb6, Arl6ip1, Psmb9, H6pd, Sdf2l1, Plp2, Tmtc3, Hsp90b1, Tmem50b, Dnajc10, Selenof, Pdia3, Srp72, Stt3a, Eogt, Ppic	*Down:* Hspa1b, Tmc8, Ankzf1, Crebrf, Josd2, Cdip1, Hspbp1, Dnajb2, Dnaja4, Stt3b, Atf4, Herpud1, Ddrgk1, Ubc, Ubxn6, Ergic3
Autophagy/Lysosome	
Up: Dram1, Ifi47, Arsb, Laptm5, Scpep1, Wwp1, Def8, Snap29, Atp6v0e, Dap	*Down:* Arsg, Tecpr1, Bbc3, Clcn6, Bloc1s3, Pdcd4, Cd63, Dapk1, Lmbrd1, Maf1, Sbf2, Ctsl, Plekhm1, Usp36
Other	
Up: Acat3, Ampd3, Fggy, Slc43a3, Suv39h1, Rnpep, Qsox1, Eif3h	*Down:* Aoc2, Slc22a8, Cyp4b1, Rnf167, Ephx1, Ppdpf, Ubxn7, Pex6, Osbpl7, Kdm4b, Nol6
**Inflammation**	
MHC, Inflammasome, Viral Response, Leukocyte Adhesion, Coagulation	
Up: Selp, H2-Q6, H2-Q7, Oas1a, F5, Nlrc5, 9930111J21Rik2, C3, Naip2, Ifitm1, Tgtp1, Ikbke, Ulbp1, Vcam1, Raet1d, B2m, H2-Q4, Ly75, Gbp5, Tmem173, Samd9l, Irgm1, B3gnt3, Ddx58	*Down*: Adgre5, Rfxank, Fcgrt, Otud5
Cytokines, Chemokines, Arachidonic Acid and Toll-Like Receptor Signaling	
Up: Serpina3n, Trim30d, Cxcl2, Cxcl1, Acod1, Alox12, Ccl2, Pla2r1, Cd14, Cxcl16, Pla2g4a, Tmem106a, Tgfb2, Tlr2, Irak3, Csf1, Il1r1, Ptgfrn, Litaf, Lmo7, Trim30a	*Down:* Tgfbr3l, Msmp Slco2a1, Abcc10, Tnfrsf8, Cish, Plpp6, Tsc22d3, Zfp36, Slco2b1, Mfhas1, Jmjd8, Lrrfip2, Nsmaf, Sash1, Rnf216
Blood Flow-Related	
Up: Traf3ip2, Ace, Ddah1, C1qtnf1, Agtrap, Ece1, Ankrd28	*Down*: Pcsk6, Npr3, Ednrb, Ramp3, Nos2, Spsb1, Nos3, Sik1, Ramp2, Npr1

**Signaling**	
NR	
Up: Aldh1a1, Bhlhe41, Ezh2, Abca4, Ncoa7, Rdh11, Kank2	*Down*: Nr1d2, Nr1d1, Klf15, Per3, Dhrs3, Klf4, Fam57b, Rdh13, Lpin1, Rara, Klf2, Rarg, Per2, Per1, Nrip1, Irf2bp2, Klf3, Zfp467, Klf9, Ncoa3, Cipc, Klf13, Nr1h2, Ncoa2
cAMP	
Up: Prkar2b, Pde3b, Adcy3, Pkia, Rab32, Pde5a	*Down*: Pde4c, Rasgrp2, Abcc4, Pde2a, Rapgef4, Sipa1l3, Rapgef5, Pde7b, Akap1, Rgl2, Rapgef3, Adcy4, Akap8
AMPK	
Up: C330027C09Rik, Impad1	*Down*: Deptor, Ppp2r5a, Ddit4, Sema6d, Flcn, Ppp2r3d, Prkag2, Stk11ip
MAPK	
Up: Pbk, Mapk8ip1	*Down*: Dusp4, Map3k6, Map3k1, Gpr182, Mapk8ip3, Map3k2, Dstyk, Rps6ka1, Dok4, Map2k7
Rho	
Up: Depdc1b, Ect2, Arhgef39, Racgap1, Iqgap3, Arhgap19, Plekhg3, Arhgef17, Rnd1, Dock5, Fam49a, Rock2, Fgd1, Rhog	*Down*: Casz1, Stard13, Arhgef3, Fam65b, Dock6, Mcf2l, Pkp4, Araf; Arhgef1, Arhgef12, Aamp
PI-Related	
Up: Pik3cd, Dapp1, Plcg2, Sdcbp2, Plekha2, Pxdc1	*Down*: Fgfbp1, Pik3r2, Tmem150a, Pik3ip1, Dgkz, Plcg1, Mtmr3, Prkch
Wnt	
Up: Wnt5b, Reck	*Down*: Lmbr1l, Dvl2, Tnik, Nkd1, Dvl1
Thyroid	
Up: Trip13, Hmgn3	*Down*: Slco1c1, Slco1a4, Zfp764, Thra
LPA:	
Up: Lpar4, Plpp2, Pld1, Lypla1	*Down*: Acp6, Enpp2
Notch	
Up: Twsg1, Kdelc2	*Down*: Maml3, Mib2, Rfng, Jag2, Dtx3, Mfng
ADAM	
Up: Adam12, Adam9, Adam17	*Down*: Adamts10
AHR	
	Down: Ahr, Ivns1abp, Calcoco1
Other	
Up: Alpl, Hmmr, Sgpp2, Susd2, P2ry1, Rhbdl2, Shcbp1, Tmeff1, Nlrc3, Lrg1, Arhgap33, Hn1, Dhh, Enpp5, Arl4c, Skap2, Ptprj, Gbp3, Eif2ak2, Hdgf, Ywhah, Aida, Arf4, Dcbld2,	*Down*: Wnk4, Srms, Gnal, Arl4d, Ret, Gipc3, Dmpk, Map3k14, Acvr2b, Sned1, Rsrp1, Spry1, Ptpn14, Sema4c, Fam212b, Gtpbp6, Tsc22d1, Pim3, Ip6k1, Efna1, Stat6, Trib3, Tec, Tgfbr3, Shkbp1, Stk17b, Ephb4, Clk2, Hdac5, Stk40, Tspan13, Efnb1, Ppfibp1, Bri3, Egfl7, Mark3, Sufu, Ric8a
**Cell cycle, cell division and cell polarization**	
Mitosis	
Up: Cenpe, Cep55, Cenpf, Knstrn, Knl1, Plk1, Ccsap, Aurka, Kif18a, Nuf2, Bub1, Kif11, Kif18b, Tpx2, Cenpa, Cenpq, 2700099C18Ri,Ska3, Cenpn, Tacc3, Cenpi, Spdl1, Ercc6l, Spc25, Oip5, Cenpm, Ndc80, Incenp, Cenph, Gsg2, Spc24, Nsl1, Cep128, Plk4, Bora, Cenpl, Cep112, Cdk5rap2, Pmf1, Cenpc1, Ska2, Hjurp, Chaf1a, Cenpj, Cntrob, Mis12, Cep295, Ccp110, Wdr62, Cep192, Nde1, Bub3, Sugt1, Nin	*Down:* Akna
DNA Replication/Damage/Repair	
Up: Ddias, Parpbp, Gen1, Pif1, Kif4, Neil3, Arhgap11a, Top2a, Fignl1, Pclaf, Fanca, Brip1, Ankle1, Polq, Aunip, Apitd1, Rad51, Eme1, Fancd2, Bard1, BC030867, Tk1, Brca2, Fancb, Pole, Cdc45, Dna2, Exo1, Polh, Tyms, Rad54l, Zranb3, Rad18, Mms22l, Lig1, Hat1, Rpa2m, Fen1, Blm, Rhno1, Mcm4, Rad50, Rfwd3, Pcna, Topbp1, Nasp, Usp1, Smc6, Rad21, Dtx3l, Rexo2, Swi5	*Down:* Mettl22, Msh2, Faap100, Smarca2, Epc2, Samhd1, Ddx54, Ddx5

General Proliferation	
Up: Aspm, Cdkn2a, Kif2cm Prr11, Cdca2, Spag5, Tmem45a, Cdkn3, H1fx, Stil, Psrc1, Ube2c, Nusap1, Fam83d, Sgol1, Sgol2a, Mki67, Prc1, Cdca3, Gas2l3, Mybl1, Ncapg, Fam64a, Ckap2l, Mastl, Zwilch, Birc5, Kifc1, Troap, Kif14, Kif22, Espl1, Ttk, Cdca8, Cks2, Cdc25c, Cdca5, Sapcd2, Melk, Aurkb, Mns1, Cit, Gpsm2, Ncapg2, Kntc1, Rrm2, Brca1, Mcm10, Ncaph, Smc2,Tmem184c, Phf19, Cks1b, Lmnb1, Asf1b, Ncapd2, Reep4, Prps2, Dbf4, Gins2, Dtl, Hells, Ube2s, Cdca7l, E2f2, Pola1, Smc4, Ccdc34, Klhl13, Rnf157, H2afx, Mcm3, H2afz, Rbl1, Pinx1, Vbp1, Rassf2, G2e3, Mcm6, Nucks1, Nudt5, Lmnb2, Ncapd3, Cse1l, Pcbp4, Dek, Paics, Tmem123	*Down: Pbld1, Bmf, Arid3a, Cgref1, Stard9, Sema4b, Cdk20, Nxpe4, Bcl2l11, Mllt6, Gltscr1, Gse1, Fam84b, Dhx34, Klhdc8b, Tnfrsf25, Cbx7, Arrdc2, Ccnj, Rbak, Pcgf2,Zer1, Ezh1, Mau2, Heca, Smyd2, Auts2, Impdh1, Mxd4, Kmt2d, Trim39, Jmjd6, Dot1l, Ubald1, Kifc3*
Cell Cycle/EMT	
Up: Ccnb2, Depdc1a, Esco2, Dlgap5, Ccnb1, Cdc20, Nek2, Ccna2, Chek1, Cdk1, Bub1b, Cdc25b, Ccnf, Foxm1, Orc1, Gtse1, Mad2l1, Fstl1, Cdkn2c, Ccne2, Gmnn, Mlf1, Clspn, Ticrr, Ndrg4, Nabp1, Rrm1, Chtf18, Lin9, Mybl2, Cdkn2d, Dck, Uhrf1, Fbxo5, Pkmyt1, Gcnt2, Cdt1, Skp2, Cdc6, Tcf19, Mcm5, Prim2, Tfdp1, Plagl1, Cnp, Fam111a, Cdc27, Anp32b	*Down:* Megf6, Neurl2, Akap8l, Ypel3, Slfn5, Fbxl12, Hdac4, Brwd1, Deaf1, Ciz1, Cdk2ap2, Dcaf8
**Adhesion**	
Extracellular Matrix	
Up: Fbln1, Spp1, Col8a1, Ptn, Ism2, Thbs1, Lamc2, Col5a2, Fn1, P4ha2, Nid1, Timp2, Mxra8, Col18a1, Col4a1, Colgalt1, Tram2, Emcn	*Down:* Col13a1, Efemp1, Mmrn1, Mmp15, Timp3, Chst15, Mmp28, Sparcl1, Hyal2
Matrix Binding/General	
Up: Lrrn3, Sulf1, Fermt3, Bst1, Antxr1, Lgals1, Itga2, Limd2, Lgals3bp, Ntn4, Ptprs, Itgav, Ext1, Itgb1, Itga1	*Down:* Adam11, Sema5a, Itga2b, Mcam, Adgrl3, Dab2
Cell-Cell Adhesion	
Up: Plekha7, Gja5, Pcdh19	*Down*: Cadm1, Cnst
**Cytoskeleton**	
Actin	
Up: Trim59, Diaph3, Fblim1, Anln, Coro2a, Iqgap2, Palld, Tln2, Cotl1, Myo5a, Gm9844, Lcp1, Tmod2, Aif1l, Sorbs2, Csrp2, Sept7, Lima1, Tes, Twf1, Tpm4, Syne2, Lasp1, Sept8, Tmsb4x, Rdx,	*Down:* Parvg, Flnc, Kank3, Cttnbp2, Parvb, Rcsd1, Epb41, Capg, Abi3, Ppp1r12c
Microtubule and Other	
Up: Ckap2, Stmn1, 4930427A07Rik, Ckap5, Tuba1b, Ckap4, Marcksl1, Tuba1c, Filip1l, Sept10, Sept11, Myl12b, Lpp, Vim	*Down:* Mpp3, Dcdc2b, Unc45b, Ushbp1, Map7, Sh2d3c, Mtss1, Camsap2, Shroom2
**Ions**	
Calcium	
Up: Mgp, Nrgn, Cald1, Mcub, Nptxr, Rcan2, S100a11, Calm2, Calm3, Capn1, Calu, Cpne2, 1810037I17Rik	*Down:* Cacna1e, Cpne7, Ss18l1, Camk2a, Camk2n1, Lmcd1, Slc25a23, Doc2b, Nfatc1, Cracr2b, Tprgl, Cabin1
Osmotic/pH/General	
Up: Scn3b, Kcnab2, Cftr, Slc4a8, Dlg4, Mt2, Mt1, Lynx1, Slc9a5, Mmgt2, Slc9a3r1, Clic6, Trpm6, Lrrc8c, Mtf1	*Down: Cnih2, Tmem100, Slc30a1, Aqp11, Tmc4, Kctd12, Gabbr1, Slc9a3r2, Slc39a10, Cnnm3 Slc22a23*

**Other**	
Gene Transcription/Translation	
Up: Scml2, Mis18bp1, Zdhhc2, Snai2, Mxd3, E2f8, E2f7, Gli3, Prim1, Hmgn2, Lmo1, Paip2b, Eif4e3, Rangap1, Zmynd15, Ell2, Dnmt1, Tceal8, Lhx6, Zfp367, Tarsl2, Arid5b, Pcbp3, Tsen34, Hnrnpa1, Tmpo, Pbx3, Zfp948, Rars, Pabpc1, Zc3h15, Cggbp1	*Down:* Zfp14, AY036118, Hopx, Tox, Tfap4, Sox5, Tbx6, Dbp, Ebf4, Tef, Trdmt1, Zfp692, Prdm1, Kdm6b, Ets2, Swsap1, Adarb1, Zfp369, Hdac10, E4f1, Cirbp, Foxl2os, Firre, Zfp691, Foxl2, Zfp74, Tet3, Zfp703, Zbtb39, Cbfa2t3, Sertad1, Kmt5b, Zfp511, Med13l, Tshz2, Mrgbp, Polr3e, Zfp839, Zfp236, Prpf40b, Phf1, Six5, Ccnl2, Endov, Exosc5, Rbm20, Ldb2, Zbtb46, Zic2, Zfp219, Atad2b, Scand1, Pan2, Taf8, Plekhn1, Zfp628, Zfp704, Chd3, Sox12, Foxf1, Kat6b, Cnot10, Ier2, 2810403A07Rik, Cars, Foxj2, Pcif1, Ldb1, Setd1b, Gata2, Arid1b, Rps27, Zfp318, Clasrp, Bcorl1, Rbm33, Zfp598, Phf12, Ell, Luc7l, Kat5, Srrm2, Sfswap, Nxf1, Adat3, Zcchc14, Cxxc1
Miscellaneous	
Up: Aipl1, Sdk1, Art3, Xkr5, Lockd, Mirt1, Glipr2, Slfn9, Kpna2, Hyls1, Bex3, Kif24, Iqcg, Dnah8, St8sia6, Kirrel, Snx10, Zdhhc15, Cand2, B3galnt1, Prss23, Mal, Atad2, Hn1l, Tmem138, Tmem176a, Bbs7, Klhl6, 0610007P14Rik, Ttc9c, Abcb1b, Pnp, Ier3, Fam114a1, Ostm1, Cyb5a, Senp1	*Down:* Sspo, N4bp2os, Islr2, Dnah10, 1810011O10Rik, Acrbp, Fam20a, Dhrs11, Hecw2, 4930578C19Rik, Tcp11l2, Spag1, Cutal, Crybb3, Vgll4, Abtb1, Leng8, Nbeal2, Ccdc84, Pcdhb17, Ica1l, Tmco6, Vsig2, Rnf144a, Ogt, Rnf215, Peg13, Kmt2c, Kmt2b, Lemd2
Unknown	
Up: 9530077C05Rik, 9330182L06Rik, Gm4316, Ccdc18, Ggn, 9930111J21Rik1, Lonrf2, Gm8430, Gm42031, Gm11223, Gm14221, 4930579G24Rik, Gm4070, Tceal9, 4933404O12Rik BC055324, Cystm1, Gm8995, Gm8822, Gm26782, 4930486L24Rik, BC034090, 2810025M15Rik, Ammecr1, Hrct1, Tmem176b, Tmem252, Olfr1372-ps1, 1110008P14Rik, Fam13b, 4930402H24Rik	*Down*: Gm26883, Adamtsl1, C030034I22Rik, Gm26762, F730016J06Rik, Unc45bos, 9330151L19Rik, Gm17491, Gm42418, Gm16701, A430110L20Rik, A430105J06Rik, Zrsr1, 2810410L24Rik, Gm20033, A330023F24Rik, 3000002C10Rik, Gm26532, RP23-162P10.8, Fam124a, Gm21781, 2900052L18Rik, A530084C06Rik, 2700081O15Rik, Tmem86a, 4430402I18Rik, 2810029C07Rik, Gm12258, AI480526, Wscd1, Gm15270, Odf3b, D930048N14Rik, Gm5617, 1700109H08Rik, Atxn7l2, Gm45755

Notes. Gene symbols for each category are listed in order from greatest to least magnitude of change in gene transcription in *APOE4* brain endothelial cells compared to *APOE3* brain endothelial cells.. Abbreviation Key: **NR**, nuclear receptor; **cAMP**, cyclic adenosine 3,5-monophosphate; **AMPK**, 5' adenosine monophosphate-activated protein kinase; **MAPK**, mitogen-activated protein kinase; **PI**, phosphatidylinositol; **AHR**, aryl hydrocarbon receptor; **LPA**, lysophosphatidic acid; **ADAM**, a disintegrin and metalloproteinase.

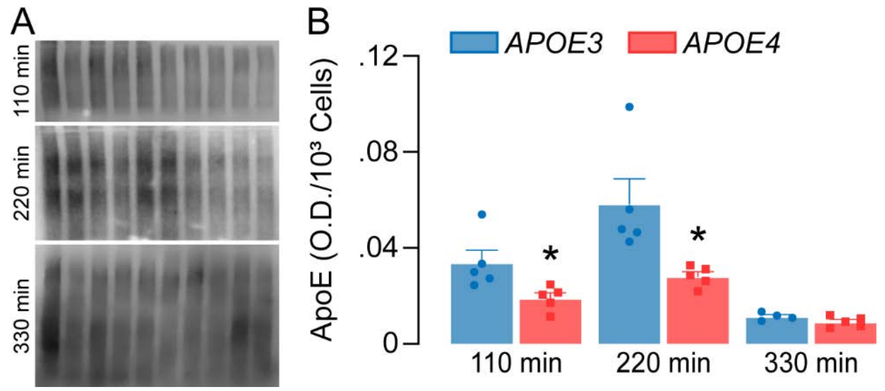

Figure S1. Brain endothelial cell-produced apoE3 and apoE4 do not migrate differently in native gel electrophoresis. Brain endothelial cell-conditioned media was separated by native gel electrophoresis on 4-20% Tris-glycine gels and assessed by western blot as an indicator of apoE lipidation. (**A**) ApoE in conditioned media separated for 110 min (top), 220 min (middle) or 330 min (bottom) migrate to a similar degree with both *APOE3* and *APOE4*. Left 5 lanes are *APOE3*, right 5 lanes are *APOE4*. (**B**) When assessed by western blot, native apoE levels are lower with *APOE4*. However, this difference is not detectable after a run time of 330 min as the signal is too diluted. Data is expressed as mean ± S.E.M. * $p < 0.05$ by Student's t-test with n = 5.

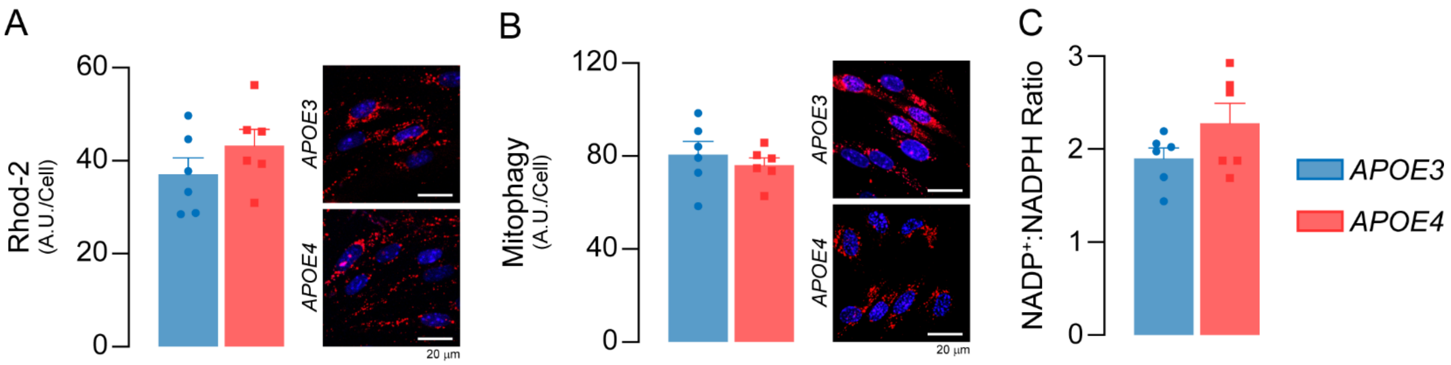

Figure S2. *APOE* genotype modulates mitochondrial function. (**A**) no differences in mitochondrial calcium levels (Rhod-2) or (**B)** mitophagy between *APOE3* and *APOE4* brain endothelial cells (Dojindo, MD01-10). (**C**) Nicotinamide adenine dinucleotide phosphate (NADP) is strongly linked to metabolism and oxidative stress and is found in oxidized (NADP+) and reduced (NADPH) forms. NADPH is used in anabolism (cholesterol, fatty acid, nucleotides) and to reduce GSSG to form GSH, thereby promoting the antioxidant glutathione pathway. Although there are lower NADPH levels in *APOE4* brain endothelial cells (**Supplementary File 2**), the NADP+: NADPH ratio did not reach significance due to high variability in NADP+ levels. Data is expressed as mean ± S.E.M. * $p < 0.05$ by Student's t-test with n = 6.

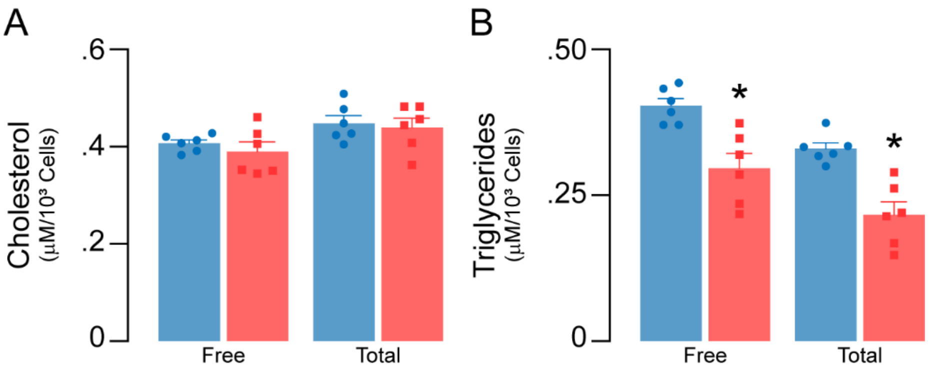

Figure S3. Triglyceride, but not cholesterol, levels are lower in media collected from *APOE4* brain endothelial cells. (**A**) Although there are no differences in cholesterol levels, (**B**) triglyceride levels are lower in media collected from *APOE4* brain endothelial cells. Blue is *APOE3*, red is *APOE4*. Data is expressed as mean ± S.E.M. *$p < 0.05$ by Student's t-test with n = 6.

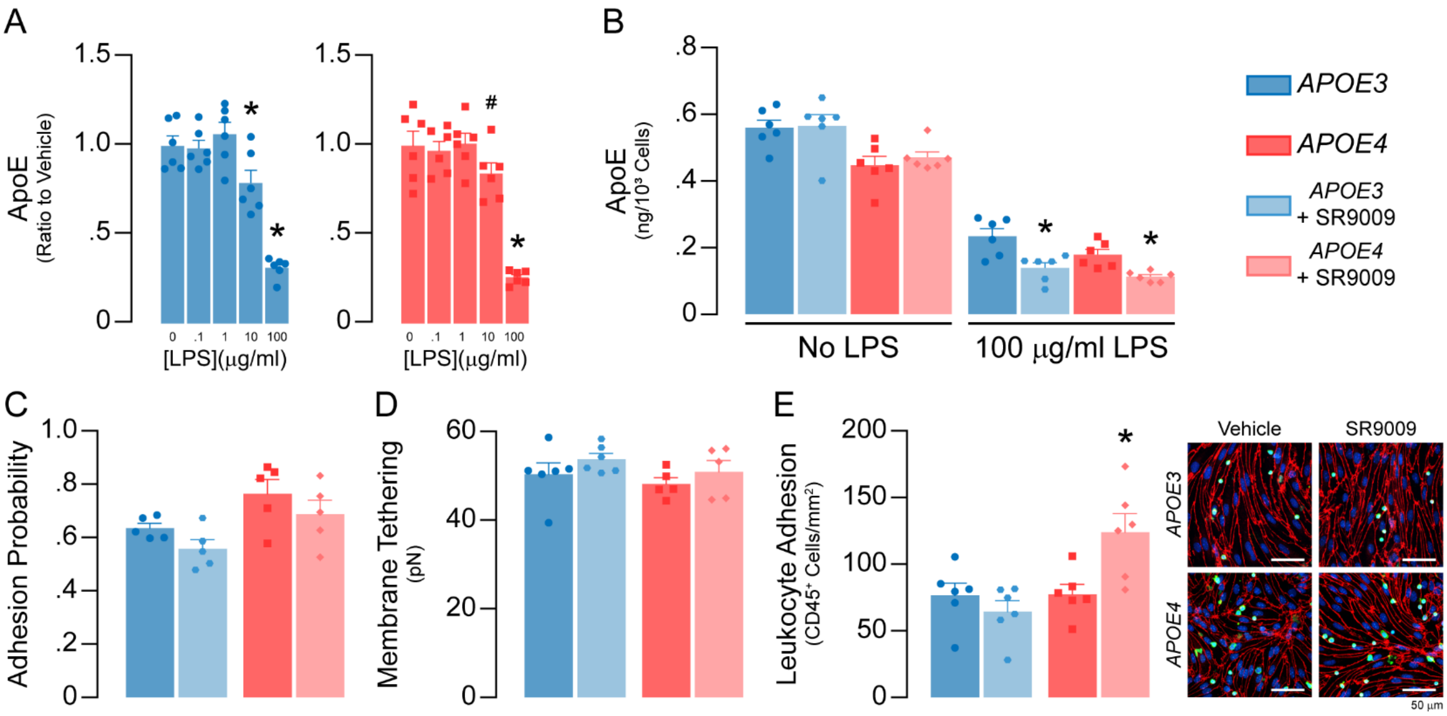

Figure S4. ApoE levels are modulated by LPS. (**A**) Both *APOE3* and *APOE4* brain endothelial cells treated with 10 and 100 µg/ml LPS for 24 h had lower levels of apoE compared to controls when measured by ELISA. (**B**) Pretreatment with 5 µM SR9009 was unable to prevent lower apoE levels that resulted from treatment with 100 µg/ml LPS. SR9009 also had no effect on (**C**) adhesion probability or (**D**) membrane tethering force (analyzed by paired *t*-test) and (**E**) increased leukocyte adhesion to *APOE4* brain endothelial cells. Data is expressed as mean ± S.E.M. * $p < 0.05$ by two-tailed Student's *t*-test and #$p < 0.05$ by one-tailed Student's *t*-test with n = 6.

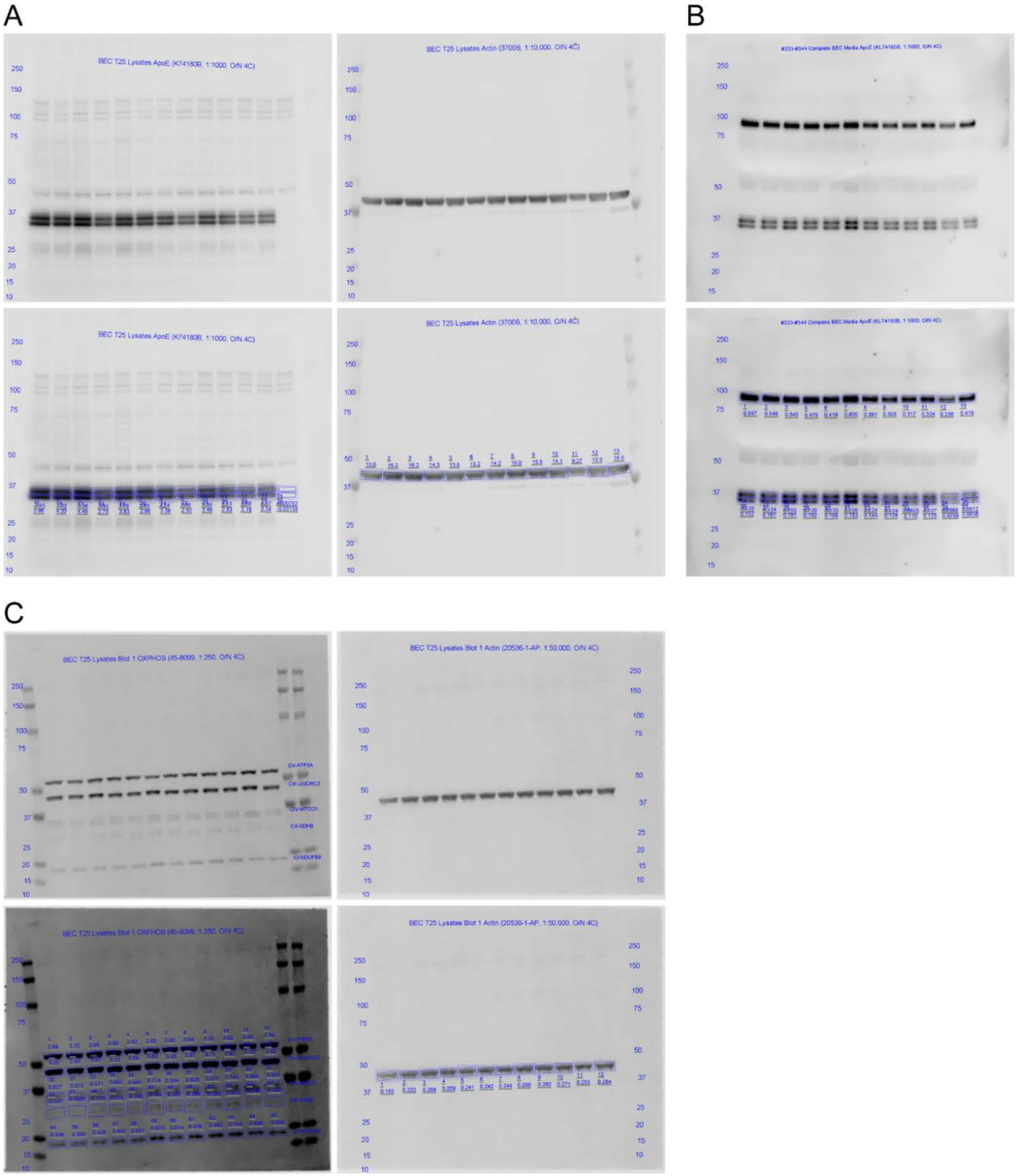

Figure S5. Full western blot images and quantification. Full western blot images and quantification of (**A**) apoE (left) and actin (right) in brain endothelial cell lysates, (**B**) apoE in media collected from brain endothelial cell cultures, and (**C**) OXPHOS antibody cocktail (left) and actin (right). Note that the OXPHOS sampler image was darkened in Image Studio Lite software to accurately trace the bands. This in no way impacts the quantification/data. Left 6 lanes are *APOE3*, right 6 lanes are *APOE4*.

3 Supplementary References

Askarova, S., Sun, Z., Sun, G.Y., Meininger, G.A., and Lee, J.C. (2013). Amyloid-beta peptide on sialyl-Lewis(X)-selectin-mediated membrane tether mechanics at the cerebral endothelial cell surface. PLoS One 8(4), e60972. doi: 10.1371/journal.pone.0060972.

Benjamini, Y., and Hochberg, Y. (1995). Controlling the False Discovery Rate: A Practical and Powerful Approach to Multiple Testing. *Journal of the Royal Statistical Society: Series B (Methodological)* 57(1)**,** 289-300. doi: 10.1111/j.2517-6161.1995.tb02031.x.

Dobin, A., Davis, C.A., Schlesinger, F., Drenkow, J., Zaleski, C., Jha, S., et al. (2013). STAR: ultrafast universal RNA-seq aligner. *Bioinformatics* 29(1)**,** 15-21. doi: 10.1093/bioinformatics/bts635.

Ichikawa, N., Naora, K., Hirano, H., Hashimoto, M., Masumura, S., and Iwamoto, K. (1996). Isolation and primary culture of rat cerebral microvascular endothelial cells for studying drug transport in vitro. *J Pharmacol Toxicol Methods* 36(1)**,** 45-52. doi: 10.1016/1056-8719(96)00072-x.

Liao, Y., Smyth, G.K., and Shi, W. (2014). featureCounts: an efficient general purpose program for assigning sequence reads to genomic features. *Bioinformatics* 30(7)**,** 923-930. doi: 10.1093/bioinformatics/btt656.

Lutz, S.E., Smith, J.R., Kim, D.H., Olson, C.V.L., Ellefsen, K., Bates, J.M., et al. (2017). Caveolin1 Is Required for Th1 Cell Infiltration, but Not Tight Junction Remodeling, at the Blood-Brain Barrier in Autoimmune Neuroinflammation. *Cell Rep* 21(8)**,** 2104-2117. doi: 10.1016/j.celrep.2017.10.094.

Marottoli, F.M., Katsumata, Y., Koster, K.P., Thomas, R., Fardo, D.W., and Tai, L.M. (2017). Peripheral Inflammation, Apolipoprotein E4, and Amyloid-beta Interact to Induce Cognitive and Cerebrovascular Dysfunction. *ASN Neuro* 9(4)**,** 1759091417719201. doi: 10.1177/1759091417719201.

McCarthy, D.J., Chen, Y., and Smyth, G.K. (2012). Differential expression analysis of multifactor RNA-Seq experiments with respect to biological variation. *Nucleic Acids Res* 40(10)**,** 4288-4297. doi: 10.1093/nar/gks042.

Micic, M., Chen, A., Leblanc, R.M., and Moy, V.T. (1999). Scanning electron microscopy studies of protein-functionalized atomic force microscopy cantilever tips. *Scanning* 21(6)**,** 394-397.

Tai, L.M., Mehra, S., Shete, V., Estus, S., Rebeck, G.W., Bu, G., et al. (2014). Soluble apoE/Abeta complex: mechanism and therapeutic target for APOE4-induced AD risk. *Mol Neurodegener* 9**,** 2. doi: 10.1186/1750-1326-9-2.

Wang, Y., Wang, N., Cai, B., Wang, G.Y., Li, J., and Piao, X.X. (2015). In vitro model of the blood-brain barrier established by co-culture of primary cerebral microvascular endothelial and astrocyte cells. *Neural Regen Res* 10(12)**,** 2011-2017. doi: 10.4103/1673-5374.172320.

Welser-Alves, J.V., Boroujerdi, A., and Milner, R. (2014). Isolation and culture of primary mouse brain endothelial cells. *Methods Mol Biol* 1135**,** 345-356. doi: 10.1007/978-1-4939-0320-7_28.
